# Supplementary material for: System‐wide optimization of an orthogonal translation system with enhanced biological tolerance
Source: Mol Syst Biol. 2023 Jul 21;19(8):e10591. doi: 10.15252/msb.202110591 (PMC10407733; doi:10.15252/msb.202110591)
Supplement: Supplementary file 1 — Appendix [file MSB-19-e10591-s002.pdf]

System-wide Optimization of an Orthogonal Translation System with Enhanced Biological Tolerance

Kyle Mohler[1,2], Jack M. Moen[3,4], Svetlana Rogulina[1,2], and Jesse Rinehart[1,2]

[1]Department of Cellular & Molecular Physiology, Yale School of Medicine, New Haven, CT 06520, USA

[2]Systems Biology Institute, Yale University, New Haven, CT 06516, USA

[3]Quantitative Biosciences Institute (QBI), University of California, San Francisco; San Francisco, CA 94158, USA. 2QBI Coronavirus Research Group (QCRG); San Francisco, CA 94158, USA.

[4]Department of Cellular and Molecular Pharmacology, University of California, San Francisco, San Francisco, CA 94158, USA.

Appendix Figures and Tables

Correspondence should be addressed to jesse.rinehart@yale.edu

Appendix Contents **Page Number**

Appendix Figure S1 3

Appendix Table S1 4

Appendix Figure S2 5

Appendix Figure S3 6

Appendix Figure S4 7

Appendix Figure S5 8

Appendix Figure S6 9

Appendix Figure S7 10

Appendix Figure S8 11

Appendix Table S2 12

Appendix Figure S9 13

Appendix Table S3 14

Appendix References 15

Appendix Figure S1:

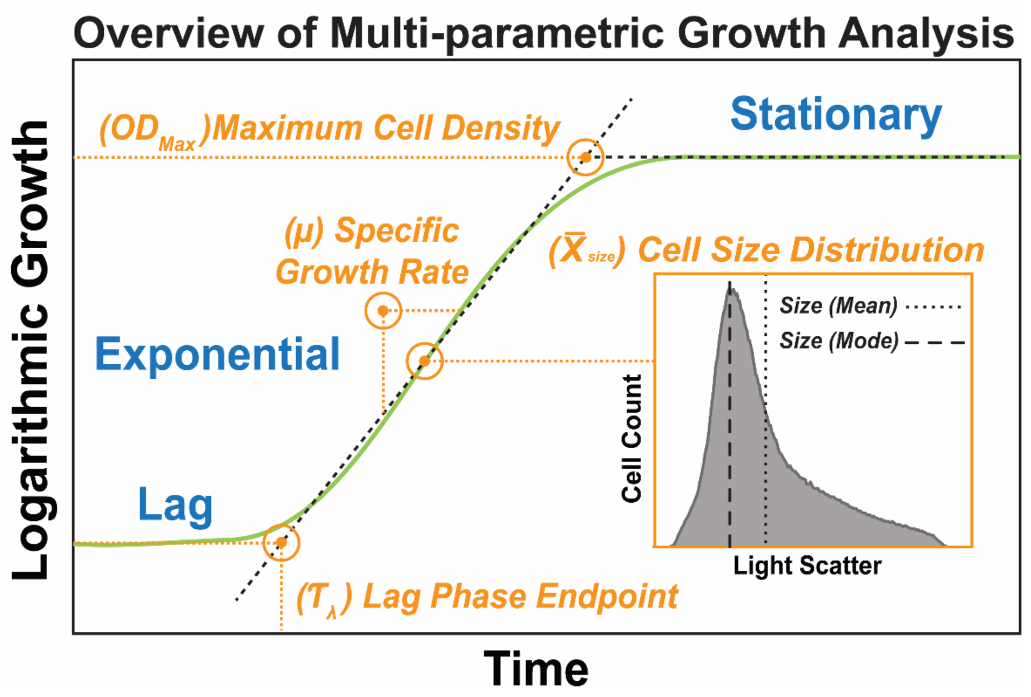

Appendix Figure S1: Graphical overview of growth analysis parameters. Cellular growth phenotypes were assessed by temporal analysis of population growth and cell size properties. Cellular growth was monitored through kinetic measurement of optical density (absorbance at 600 nm) and divided into three discrete parameters - lag phase endpoint ($\boldsymbol{T_\lambda}$), specific growth rate ($\boldsymbol{\mu}$), and growth efficiency (maximum cell density, $\boldsymbol{OD_{Max}}$) – using PreCog analysis software. Cell size was measured by flow cytometry using forward scatter (FSC) as a cell-size proxy. The population cell size distribution ($\boldsymbol{\bar{X}_{size}}$) was analyzed using statistical parameters (mean and modal cell size) determined using FlowJo analysis software.

Appendix Table S1:

Appendix Table S1: Cellular growth parameters used to assess growth phenotypes and relative fitness

Host Cell	Ori. of Rep.	tRNA Copy Number	Growth Rate (μ)	*S.D.*	Lag Time (min., T_{λ})	*S.D.*	Max Cell Density ($O.D._{Max}$)	*S.D.*	Cell Size (Mean, $\bar{X}$)	Cell Size (Mode)	*C.V.*	Relative Fitness
rEcoli^{XpS}	-	-	0.040	0.007	71	10	3.58	0.47	93155	62464	48.2	1.00
rEcoli^{XpS}	pSerOTSλ	5x tRNA	0.015	0.002	210	6	1.06	0.02	31255	20480	49.2	0.34
rEcoli^{XpS}	p15a	1x tRNA	0.028	0.007	74	10	2.75	0.04	28456	19456	67.5	0.73
rEcoli^{XpS}	p15a	2x tRNA	0.022	0.001	86	7	2.49	0.03	46596	24576	71.3	0.64
rEcoli^{XpS}	p15a	4x tRNA	0.019	0.002	81	6	2.56	0.05	37565	21504	70.7	0.62
rEcoli^{XpS}	p15a	6x tRNA	0.015	0.002	90	1	2.71	0.12	34593	20480	61	0.57
rEcoli^{XpS}	ColE1 + Rop	1x tRNA	0.026	0.004	86	11	1.31	0.04	43429	25600	55	0.58
rEcoli^{XpS}	ColE1 + Rop	2x tRNA	0.026	0.004	81	3	1.26	0.02	42267	27648	55.3	0.59
rEcoli^{XpS}	ColE1 + Rop	4x tRNA	0.021	0.001	111	18	1.26	0.04	45235	30720	56.1	0.50
rEcoli^{XpS}	ColE1 + Rop	6x tRNA	0.028	0.003	85	8	1.29	0.07	60398	45056	49.1	0.64
rEcoli^{XpS}	ColE1	1x tRNA	0.039	0.001	72	5	2.80	0.01	63108	41984	70.1	0.85
rEcoli^{XpS}	ColE1	2x tRNA	0.022	0.001	106	1	2.42	0.01	35997	15360	105	0.57
rEcoli^{XpS}	ColE1	4x tRNA	0.014	0.001	132	9	2.46	0.07	36570	21504	63.1	0.49
rEcoli^{XpS}	ColE1	6x tRNA	0.004	0.001	242	40	1.51	0.03	81828	41984	64.4	0.40

Host Cell	Ori. of Rep.	Isolated Component	Growth Rate (μ)	*S.D.*	Lag Time (T_{λ})	*S.D.*	Max Cell Density ($O.D._{Max}$)	*S.D.*	Cell Size (Mean, $\bar{X}$)	Cell Size (Mode)	*C.V.*	Relative Fitness
rEcoli^{XpS}	ColE1	glnS*-pSerRS	0.021	0.002	79	3	1.63	0.03	52645	38912	49	0.61
rEcoli^{XpS}	ColE1 + Rop	TRC*-pSerRS	0.012	0.001	279	97	1.13	0.02	36565	22528	59.2	0.32
rEcoli^{XpS}	ColE1	2x tRNA Only	0.025	0.001	113	18	1.39	0.05	*N.D.*	*N.D.*	-	0.55
rEcoli^{XpS}	ColE1	4x tRNA Only	0.020	0.003	121	16	1.19	0.01	*N.D.*	*N.D.*	-	0.48
rEcoli^{XpS}	ColE1	6x tRNA Only	0.018	0.005	140	24	1.04	0.01	*N.D.*	*N.D.*	-	0.42
rEcoli^{XpS}	ColE1 + Rop	λ tRNA Only	0.020	0.002	96	14	1.06	0.02	*N.D.*	*N.D.*	-	0.51

Host Cell	Ori. of Rep.	tRNA Copy Number	Growth Rate (μ)	*S.D.*	Lag Time (T_{λ})	*S.D.*	Max Cell Density ($O.D._{Max}$)	*S.D.*	Cell Size (Mean, $\bar{X}$)	Cell Size (Mode)	*C.V.*	Relative Fitness
BL21	-	-	0.057	0.003	72	12	3.42	0.23	*N.D.*	*N.D.*	-	1.00
BL21	p15a	1x tRNA	0.033	0.002	73	4	1.16	0.03	*N.D.*	*N.D.*	-	0.63
BL21	p15a	2x tRNA	0.048	0.004	79	4	2.76	0.47	*N.D.*	*N.D.*	-	0.85
BL21	p15a	4x tRNA	0.041	0.001	85	1	2.48	0.05	*N.D.*	*N.D.*	-	0.76
BL21	p15a	6x tRNA	0.039	0.002	84	1	2.50	0.11	*N.D.*	*N.D.*	-	0.76
BL21	ColE1 + Rop	1x tRNA	0.027	0.001	70	0	1.32	0.03	*N.D.*	*N.D.*	-	0.63
BL21	ColE1 + Rop	2x tRNA	0.022	0.001	73	4	1.16	0.03	*N.D.*	*N.D.*	-	0.57
BL21	ColE1 + Rop	4x tRNA	*N.V.*	*N.V.*	*N.V.*	*N.V.*	*N.V.*	*N.V.*	*N.V.*	*N.V.*	*N.V.*	*N.V.*
BL21	ColE1 + Rop	6x tRNA	*N.V.*	*N.V.*	*N.V.*	*N.V.*	*N.V.*	*N.V.*	*N.V.*	*N.V.*	*N.V.*	*N.V.*
BL21	ColE1	1x tRNA	0.025	0.001	79	2	1.19	0.03	*N.D.*	*N.D.*	-	0.57
BL21	ColE1	2x tRNA	0.030	0.001	119	3	2.17	0.06	*N.D.*	*N.D.*	-	0.59
BL21	ColE1	4x tRNA	*N.V.*	*N.V.*	*N.V.*	*N.V.*	*N.V.*	*N.V.*	*N.V.*	*N.V.*	*N.V.*	*N.V.*
BL21	ColE1	6x tRNA	*N.V.*	*N.V.*	*N.V.*	*N.V.*	*N.V.*	*N.V.*	*N.V.*	*N.V.*	*N.V.*	*N.V.*

N.D. **(Not Determined); N.V. (Not Viable)**

Appendix Figure S2:

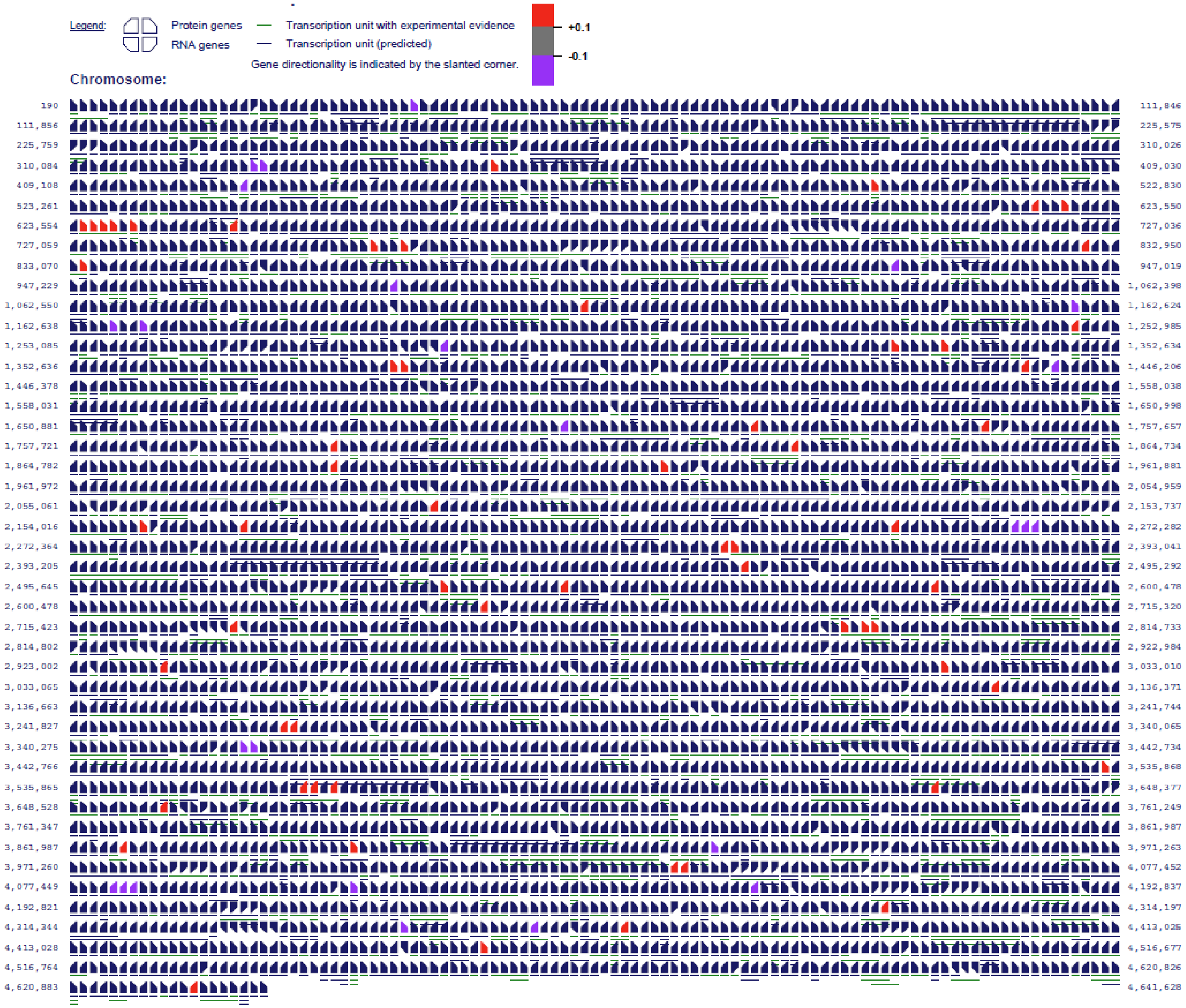

Appendix Figure S2: Alterations to proteome composition mediated by low-toxicity [C1]. Proteomic analysis of rEcoli^{XpS} cells with and without expression from **[C1]** was conducted using Perseus. Proteins with statistically significant up-regulation (red) or down-regulation (purple) were matched to their corresponding gene in relation to the complete *E. coli* genome (navy) using Pathway Tools. Proteomes were obtained in triplicate and statistical significance was determined by t-test with $p<0.05$.

Appendix Figure S3:

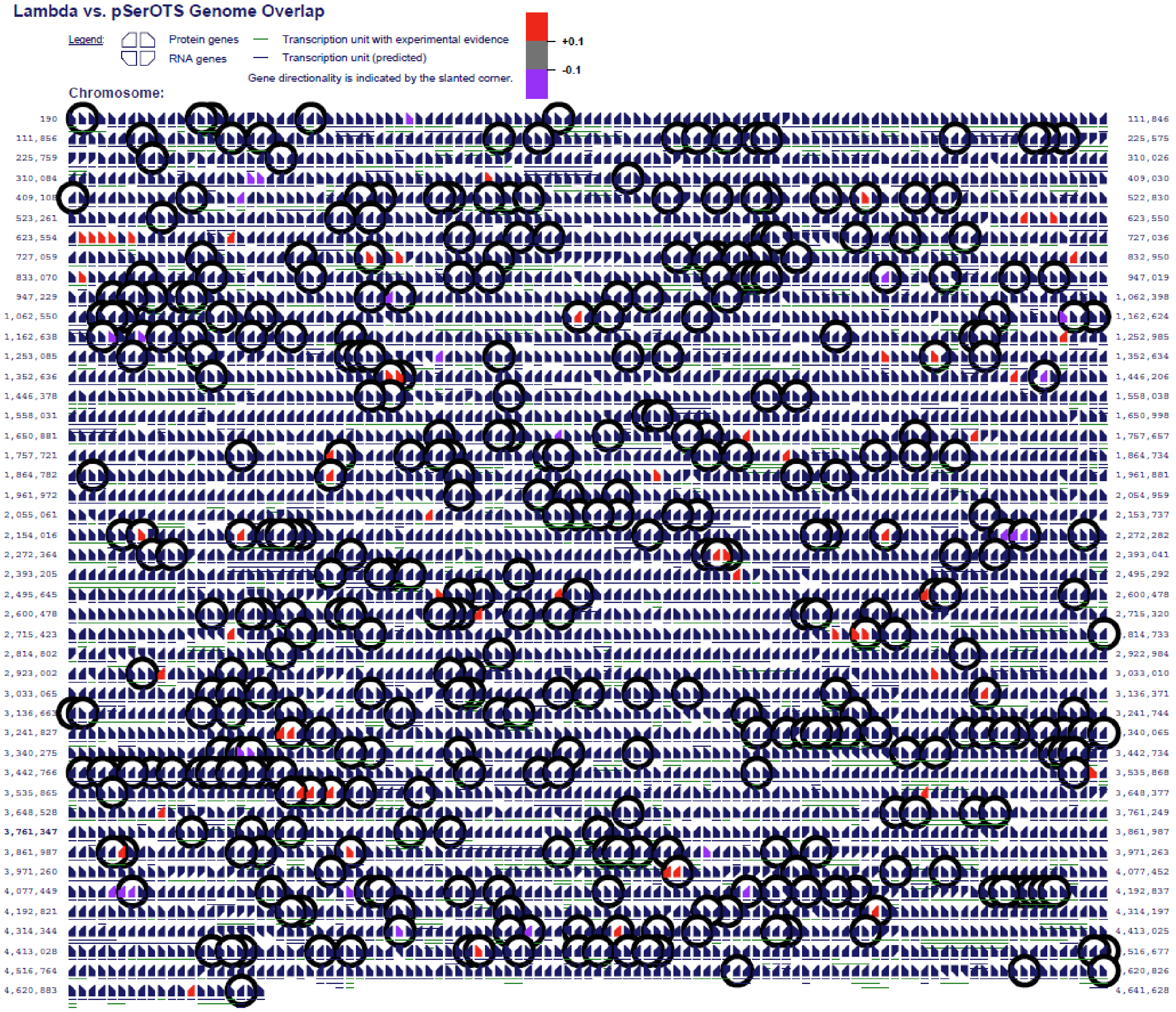

Appendix Figure S3: Alterations to proteome composition mediated by high-toxicity [λ]. Proteomic analysis of rEcoli^{XpS} cells with and without expression from **[λ]** was conducted using Perseus. Proteins with statistically significant up- or down-regulation in the presence of **[λ]** are indicated with black circles, and for **[C1]** up-regulation (red) or down-regulation (purple). All proteins were matched to their corresponding gene in relation to the complete *E. coli* genome (navy) using Pathway Tools. Proteomes were obtained in triplicate and statistical significance was determined by t-test with $p<0.05$.

Appendix Figure S4:

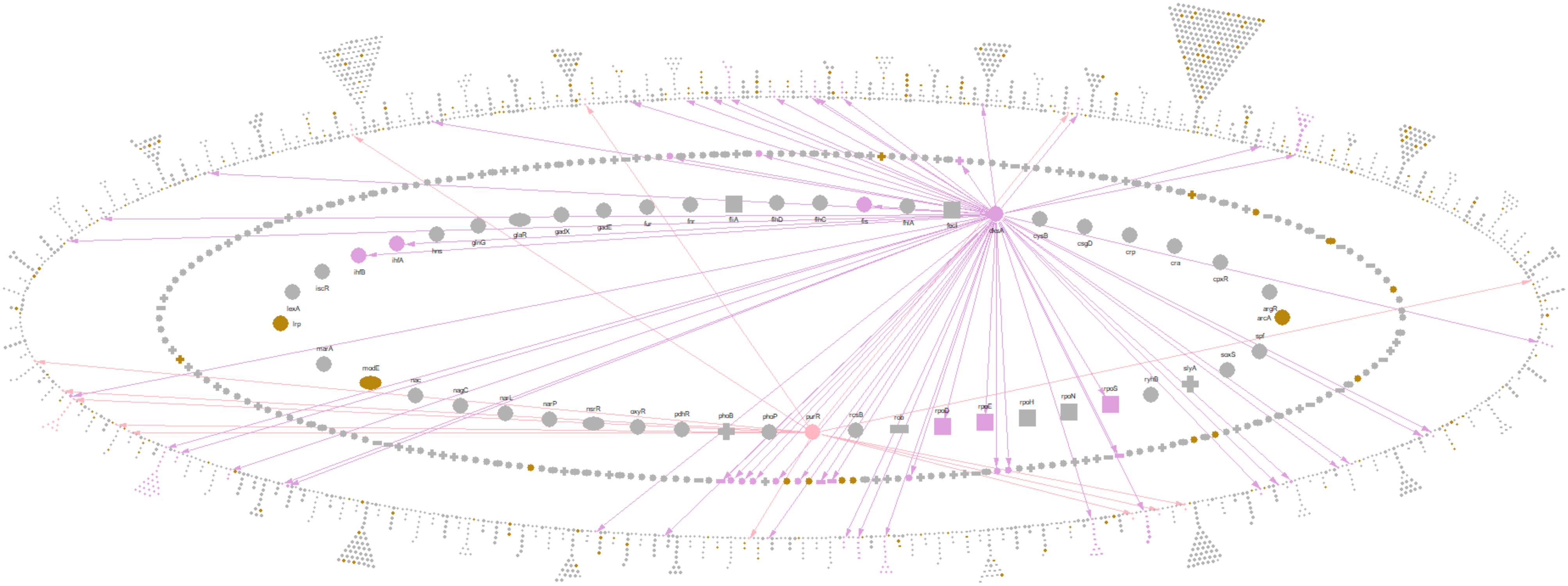

Appendix Figure S4: Expression from high-toxicity [λ] OTS perturbs transcriptional regulation in genomically recoded cells. Proteome dysregulation in cells expressing **[λ]** was mapped to the *E. coli* transcriptional regulatory network using Pathway Tools. Two of the most affected regulons (controlled by DskA and PurR) are highlighted in purple and pink, respectively. The corresponding color-coded arrows and nodes constitute dysregulated interactions across respective regulons. Nodes colored burnt yellow indicate statistically significant change in the protein abundance for that specific transcriptional regulator within cells expressing **[λ]**, relative to to WT. Protein abundance was assessed by mass spectrometry from at least three independent biological replicates and analyzed using MaxQuant. Statistical analysis was performed in Perseus software using an FDR of 0.01 and $p < 0.05$.

Appendix Figure S5:

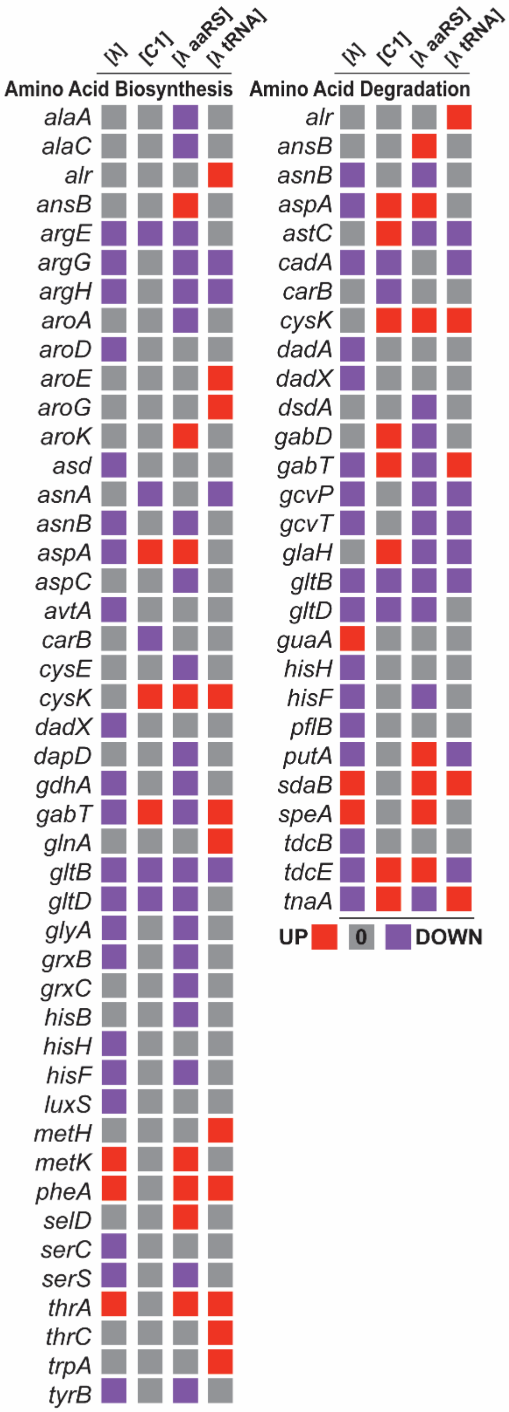

Appendix Figure S5: Isolated o-tRNA expression from [λ tRNA] displays characteristics of stringent response activation. Cells expressing only o-tRNA from **[λ tRNA]** were subjected to proteomic analysis and compared to cells expressing full (**[λ]** and **[C1]**) and partial (**[λ aaRS]**) OTS variants. Pathway enrichment analysis was conducted for strains expressing OTS variants using Pathway Tools. Dysregulated pathway components are highlighted for specific genes with the enriched pathway, with up-regulated proteins in red, down-regulated proteins in purple, and proteins with no change compared to WT host cells in grey. Enrichment cutoffs were set to a statistically significant differential expression score of 0.1. All proteomes were quantified in triplicate and analyzed in Perseus using t-test and volcano plot functions to obtain statistically significant proteomic deviations.

Appendix Figure S6:

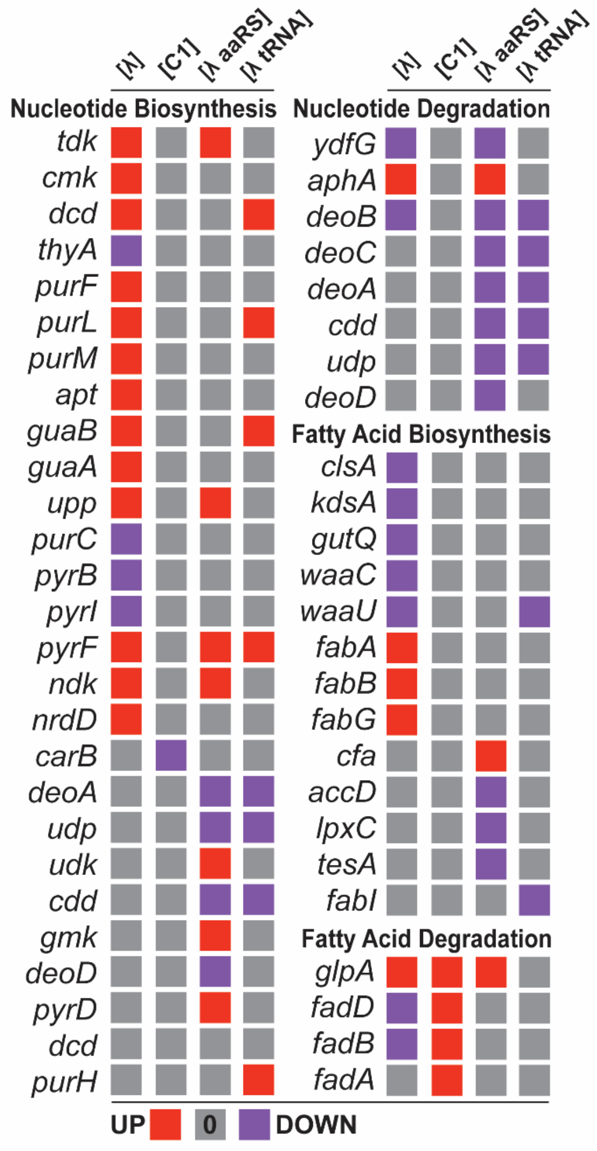

Appendix Figure S6: o-aaRS overexpression mediated dysregulation of nucleotide biosynthesis. Proteomic dysregulation from cells expressing full (**[λ]** and **[C1]**) and partial (**[λ tRNA]** and **[λ aaRS]**) OTS variants was assessed by mass spectrometry. Pathway enrichment analysis was conducted for strains expressing OTS variants using Pathway Tools. Dysregulated pathway components are highlighted for specific genes with the enriched pathway, with up-regulated proteins in red, down-regulated proteins in purple, and proteins with no change compared to WT host cells in grey. Enrichment cutoffs were set to a statistically significant differential expression score of 0.1. All proteomes were quantified in triplicate and analyzed in Perseus using t-test and volcano plot functions to obtain statistically significant proteomic deviations.

Appendix Figure S7:

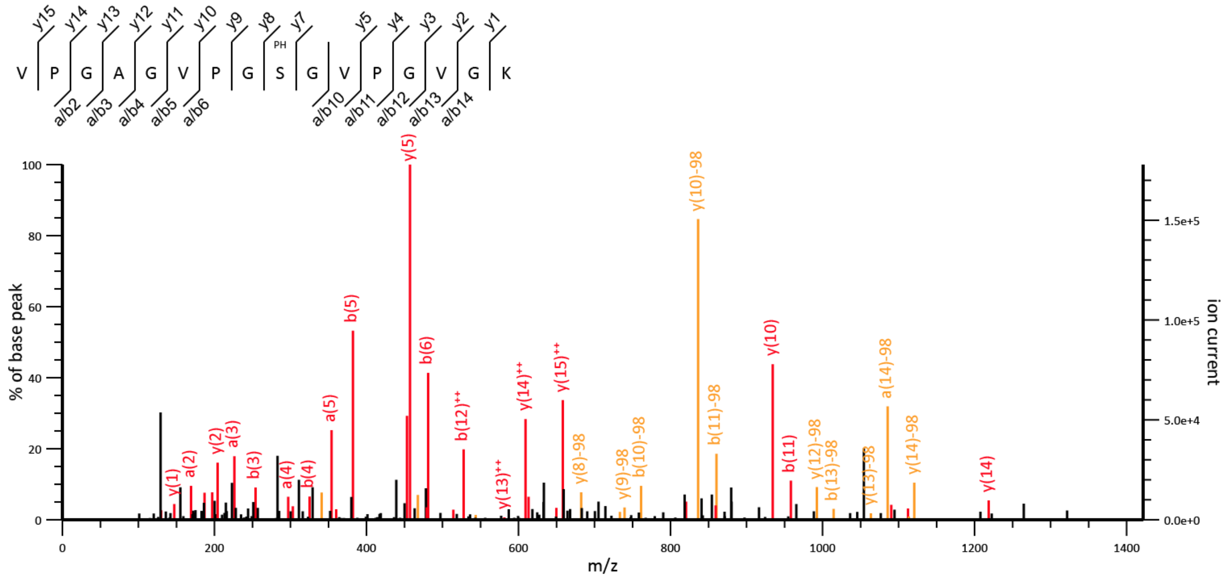

Appendix Figure S7: MS2 ion spectrum and peptide sequence confirming pSer misincorporation. MS-READ reporter proteins with a central Gly at the guest position were purified from rEcoli^{XpS} host cells expressing TRC*-pSerRS alone. Raw mass spectrometry data was processed and searched using Mascot. High confidence identification and peptide sequencing for the Gly-MS-READ reporter peptide (precursor m/z 707.858 M^{++}) identified pSer misincorporation at the Gly codon, indicated by “PH” above Ser between y7 and y8.

Appendix Figure S8:

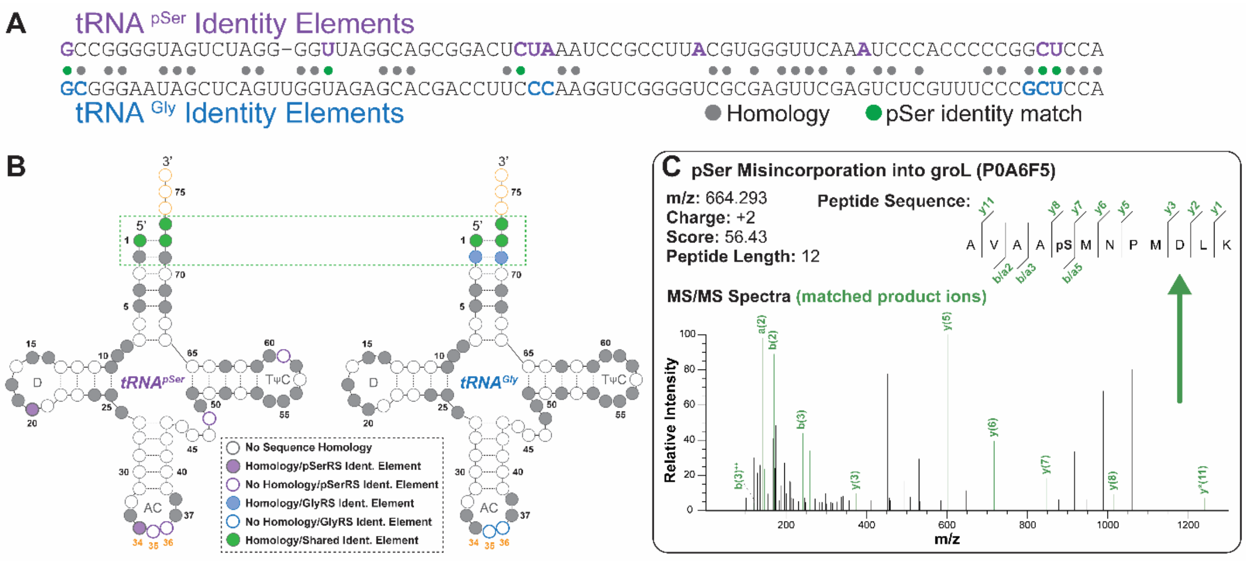

Appendix Figure S8: Proteome damage mediated by misidentification of tRNA^{pSer} by host aaRSs. pSerRS is able to recognize and productively attach pSer onto *E. coli* tRNA^{Gly} causing mistranslation of Gly codons and proteome damage. Misrecognition occurs due to overlapping tRNA identity elements. *E. coli* tRNA^{Gly} (blue) and tRNA^{pSer} (purple) were aligned to identify primary sequence homology (grey circles) and pSerRS recognition elements common to both tRNAs (green circles) (**A**) and mapped to respective tRNA secondary structures to highlight the canonical identity elements and visualize overlap (**B**). The proteomes of cells expressing **[λ]** and **[λ aaRS]** were analyzed for pSer misincorporation using custom modification parameters in Mascot software. The MS2 spectrum and sequence for example groL peptide (m/z 664.293 M^{++}) found in all samples is presented in (**C**).

Appendix Table S2:

Appendix Table S2: Peptides with Gly → pSer substitutions in the E. coli proteome

Sequence	Length	Protein	Gene	Score	Intensity	Missed Cleavages	MS/MS m/z	Charge	m/z	Retention Time
LNEL**G**LQFMQ**G**ARFWHVLDASA**G**K	24	P76329	yedP	52.541	1.33E+08	1	991.7761841	3	991.1053	93.45
DFKLKG**G**VLP**G**EQEIDTVR	19	Q46915	gudX	44.616	13255000	2	1193.029419	2	1192.533	101.89
**G**TL**G**QDVIDIRTLGSKGVFTFDP**G**FTSTASCESK	34	P0ABH7	gltA	42.241	6189200	2	1292.577148	3	1292.236	96.084
MT**G**IVKTFD**G**K	11	P0A976	cspF	52.482	3.22E+08	1	692.8035889	2	692.8028	71.94
VISEAVAAA**GG**KLQ	14	Q46800	xdhB	40.994	50758000	1	752.4145508	2	751.8488	79.704
IATLLLP**G**I**G**TIHDLK	16	P51020	mhpE	48.981	36999000	0	932.0004883	2	931.9933	106.06
T**G**L**G**RRIALILVK	13	P39414	ttdT	47.261	19178000	2	799.453186	2	799.4514	91.432
**G**ISLQVNAGEHIAIL**G**R	17	P23886	cydC	68.536	29870000	0	968.9760742	2	968.4763	86.927
IETLCRLT**G**K	10	P0A738	moaC	121.99	2.4E+08	1	642.8224487	2	642.8227	102.2
LTRPRT**G**NGPR	11	P0A8J8	rhlB	50.607		2	659.8416138	2	659.8406	68.065
LYSMYNSAFLDDLTKAM**G**R	19	P75801	yliF	40.962	33665000	1	765.0105591	3	764.3423	81.847
NL**G**QENFDAAEK	12	P25552	gppA	44.753	1.72E+08	0	716.7801514	2	716.7794	64.83
NQVLEKL**G**LNSEEQK	15	P18390	yjjA	48.741		1	913.4230957	2	913.427	65.93
NVELLT**G**FSNR	11	P76236	yeaI	47.869	30157000	0	672.8151855	2	672.8134	71.466
EPIKNEANN**G**LKNTR	15	P23869	ppiB	63.624	36029000	2	897.4296875	2	896.9331	84.654
AS**G**IPALPWEDCQ	13	P69330	citD	46.001	41994000	0	1537.645996	1	1537.639	98.059
M**G**KGKGNVEYWVALIQPGK	19	P0ADY7	rplP	52.693		2	1085.545166	2	1085.542	112.21
QVKTQSCVVA**G**KK	13	P39346	idnD	45.137		2	764.3868408	2	764.3837	68.082
AVAA**G**MNPMDLK	12	P0A6F5	groL	56.432	3005300	0	664.2930298	2	664.2929	91.465
ELESRQP**G**VR	10	P29131	ftsN	69.628	13653000	1	633.296814	2	633.2979	83.445
GETFA**G**FKQSR	11	P39452	nrdE	49.418		1	661.3015137	2	661.3005	78.103
IISPMT**G**YVSR	11	P27303	emrA	70.919	23921000	0	667.817749	2	667.3147	64.414
IV**G**YDEIFGRK	11	P30128	greB	41.029	17380000	1	695.8461304	2	695.842	87.754
QH**G**LQSMPRLRVMLNEK	17	P0AAK1	hycB	46.319	15848000	2	711.6933594	3	711.355	106.3
**G**MGESNPVTGNTCDNVK	17	P0A910	ompA	53.034	4607400	0	945.3768311	2	945.3739	73.828
IDLVQE**G**LR	9	P0AF06	motB	44.767	31797000	0	569.2811279	2	569.2812	65.859
INGATVDVRL**G**NKFR	15	P28248	dcd	48.44	18301000	2	878.9468384	2	878.4431	101.71
NTLEIVQE**G**VEAR	13	P08201	nirB	64.04	22226000	0	776.3782959	2	776.3744	83.343
SFEPL**G**LKK	9	P37197	yhjA	40.103	33542000	1	556.791687	2	556.7912	77.455
YR**G**VFGQRDVVFMSAK	16	P77561	ydeP	46.352	5.36E+08	2	986.4676514	2	985.9633	73.381
DEVILPYWRQLID**G**IK	16	P0AB89	purB	49.35	3072700	1	1027.5271	2	1027.024	114.44
IAENIKF**G**AAQ	11	P0AFX9	rseB	40.045	38493000	1	629.291626	2	629.2918	73.661
YL**G**RMHQTGR	10	P0C0K3	rdoA	53.17	3868400	1	657.7987061	2	657.2947	84.611

pSer substitution position in annotated as "**G**" in peptide sequence. Peptide in blue featured in Appendix Figure S8C.

Appendix Figure S9:

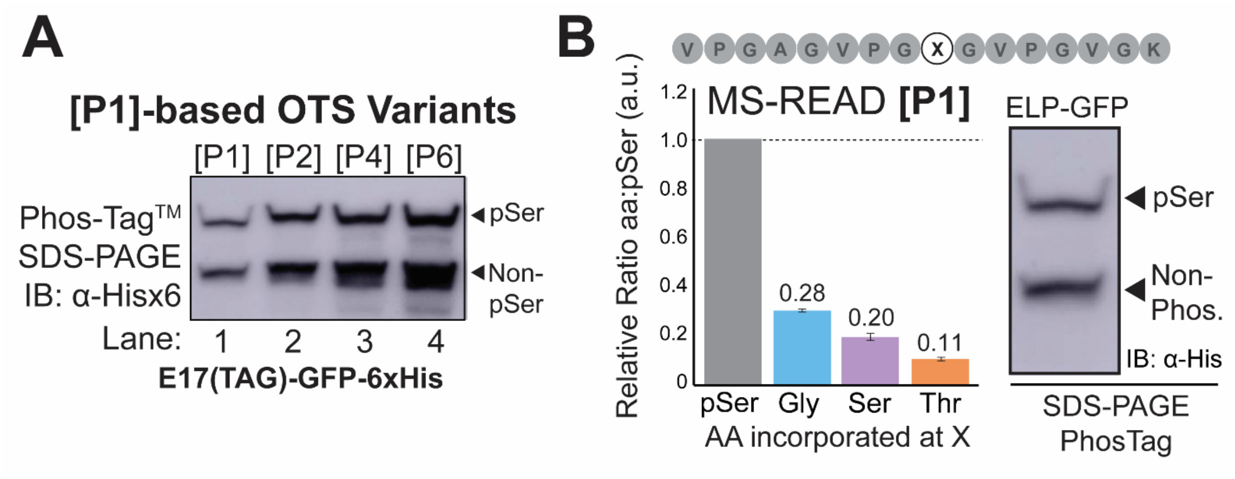

Appendix Figure S9: Reduced orthogonality of o-tRNA decreases fidelity in [P1]-based OTSs. The effect of plasmid copy number variation on OTS fidelity was assessed from cells expressing a UAG-containing reporter protein and an OTS variant (**[P1]**, **[P2]**, **[P4]**, **[P6]**) following separation by Phos-tag™ gel and immunoblot analysis; pSer-reporter protein (upper band), non-pSer-reporter protein (lower band) (**A**). Amino acid misincorporation at in cells expressing **[P1]** was assessed by mass spectrometry analysis of a UAG-containing MS-READ reporter. Amino acid incorporation events from three independent samples were quantified using Skyline and graphed relative to the level of pSer incorporation within the same sample (alongside enlargement of S9A, Lane 1 reproduced for reference as representative qualitative analysis sample) (**B**).

Appendix Table S3:

Appendix Table S3: E. coli strains and plasmids used in this study

Strains	Name	Growth Requirements	Reference
	BL21 (DE3)	No AB required	(Studier & Moffatt, 1986)
	BL21 (DE3) ΔserB	No AB required	(Steinfeld et al, 2014)
	BL21 (DE3) B-95	No AB required	(Mukai et al, 2015)
	BL21 (DE3) B-95 ΔserB	No AB required	(Zhu et al, 2019)
	C321.ΔA (C321 mutS+, λ-, Δ(ybhB-bioAB)::zeoR, ΔprfA)	Requires d-biotin	(Lajoie et al, 2013)
	rEcoli^{XpS} (C321 mutS+, λ-, Δ(ybhB-bioAB)::zeoR, ΔprfA, ΔserB)	Requires d-biotin	This Study
**Plasmids**	**Name**	**Growth Requirements**	**Reference**
E29	pSerOTSλ	Kanamycin, 37 ˚C	(Pirman et al, 2015)
G30	supD tRNA^{Ser} Suppressor	Kanamycin, 37 ˚C	(Pirman et al, 2015)
	pSerOTSc		
Q73	ColE1, proK-1x tRNA, glnS*-pSerRS9-EF-pSer21	Kanamycin, 37 ˚C	This Study
	pSerOTSc - tRNA Variants		
P73	ColE1, proK-2x tRNA	Kanamycin, 37 ˚C	This Study
P74	ColE1, proK-4x tRNA	Kanamycin, 37 ˚C	This Study
P75	ColE1, proK-6x tRNA	Kanamycin, 37 ˚C	This Study
	pSerOTSc - ColE1+ Rop tRNA Variants		
R20	ColE1 + Rop, proK-1x tRNA	Kanamycin, 37 ˚C	This Study
Q58	ColE1 + Rop, proK-2x tRNA	Kanamycin, 37 ˚C	This Study
Q59	ColE1 + Rop, proK-4x tRNA	Kanamycin, 37 ˚C	This Study
Q60	ColE1 + Rop, proK-6x tRNA	Kanamycin, 37 ˚C	This Study
	pSerOTSc - p15a tRNA Variants		
R19	p15a, proK-1x tRNA	Kanamycin, 37 ˚C	This Study
P5	p15a, proK-2x tRNA	Kanamycin, 37 ˚C	This Study
P32	p15a, proK-4x tRNA	Kanamycin, 37 ˚C	This Study
P33	p15a, proK-6x tRNA	Kanamycin, 37 ˚C	This Study
	pSerOTSc - Modified tRNA		
V70	ColE1, G2:C71 1x tRNA	Kanamycin, 37 ˚C	This Study
	OTS Components		
R4	pSerOTSλ TRC*-pSerRS only	Kanamycin, 37 ˚C	This Study
Q81	pSerOTSc glnS*-pSerRS only	Kanamycin, 37 ˚C	This Study
R9	pSerOTSλ lpp-5x tRNA only	Kanamycin, 37 ˚C	This Study
V60	ColE1, G2:C71 1x tRNA only	Kanamycin, 37 ˚C	This Study
R11	ColE1, proK-2x tRNA only	Kanamycin, 37 ˚C	This Study
R12	ColE1, proK-4x tRNA only	Kanamycin, 37 ˚C	This Study
R3	ColE1, proK-6x tRNA only	Kanamycin, 37 ˚C	This Study
	Recombinant Reporter Expression		
C9	E(17)TAG-GFP, aTc Inducible, RSF1030	Chloramphenicol, 37˚C	(Pirman et al, 2015)
E52	TAG-MS-READ mass spectrometry reporter, aTc Inducible, p15a	Ampicillin, 37 ˚C	This Study

aTc = anhydrotetracycline   IPTG= Isopropyl β- d-1-thiogalactopyranoside   Ara= Arabinose

Supplemental References

Lajoie MJ, Rovner AJ, Goodman DB, Aerni HR, Haimovich AD, Kuznetsov G, Mercer JA, Wang HH, Carr PA, Mosberg JA et al (2013) Genomically recoded organisms expand biological functions. *Science* **342:** 357-360

Mukai T, Hoshi H, Ohtake K, Takahashi M, Yamaguchi A, Hayashi A, Yokoyama S, Sakamoto K (2015) Highly reproductive Escherichia coli cells with no specific assignment to the UAG codon. *Scientific reports* **5:** 9699

Pirman NL, Barber KW, Aerni HR, Ma NJ, Haimovich AD, Rogulina S, Isaacs FJ, Rinehart J (2015) A flexible codon in genomically recoded Escherichia coli permits programmable protein phosphorylation. *Nature communications* **6:** 8130

Steinfeld JB, Aerni HR, Rogulina S, Liu Y, Rinehart J (2014) Expanded cellular amino acid pools containing phosphoserine, phosphothreonine, and phosphotyrosine. *ACS Chem Biol* **9:** 1104-1112

Studier FW, Moffatt BA (1986) Use of bacteriophage T7 RNA polymerase to direct selective high-level expression of cloned genes. *J Mol Biol* **189:** 113-130

Zhu P, Gafken PR, Mehl RA, Cooley RB (2019) A Highly Versatile Expression System for the Production of Multiply Phosphorylated Proteins. *ACS chemical biology* **14:** 1564-1572
