# Supplementary material for: System‐wide optimization of an orthogonal translation system with enhanced biological tolerance
Source: Mol Syst Biol. 2023 Jul 21;19(8):e10591. doi: 10.15252/msb.202110591 (PMC10407733; doi:10.15252/msb.202110591)
Supplement: Supplementary file 2 — Expanded View Figures PDF [file MSB-19-e10591-s006.pdf]

Expanded View Figures

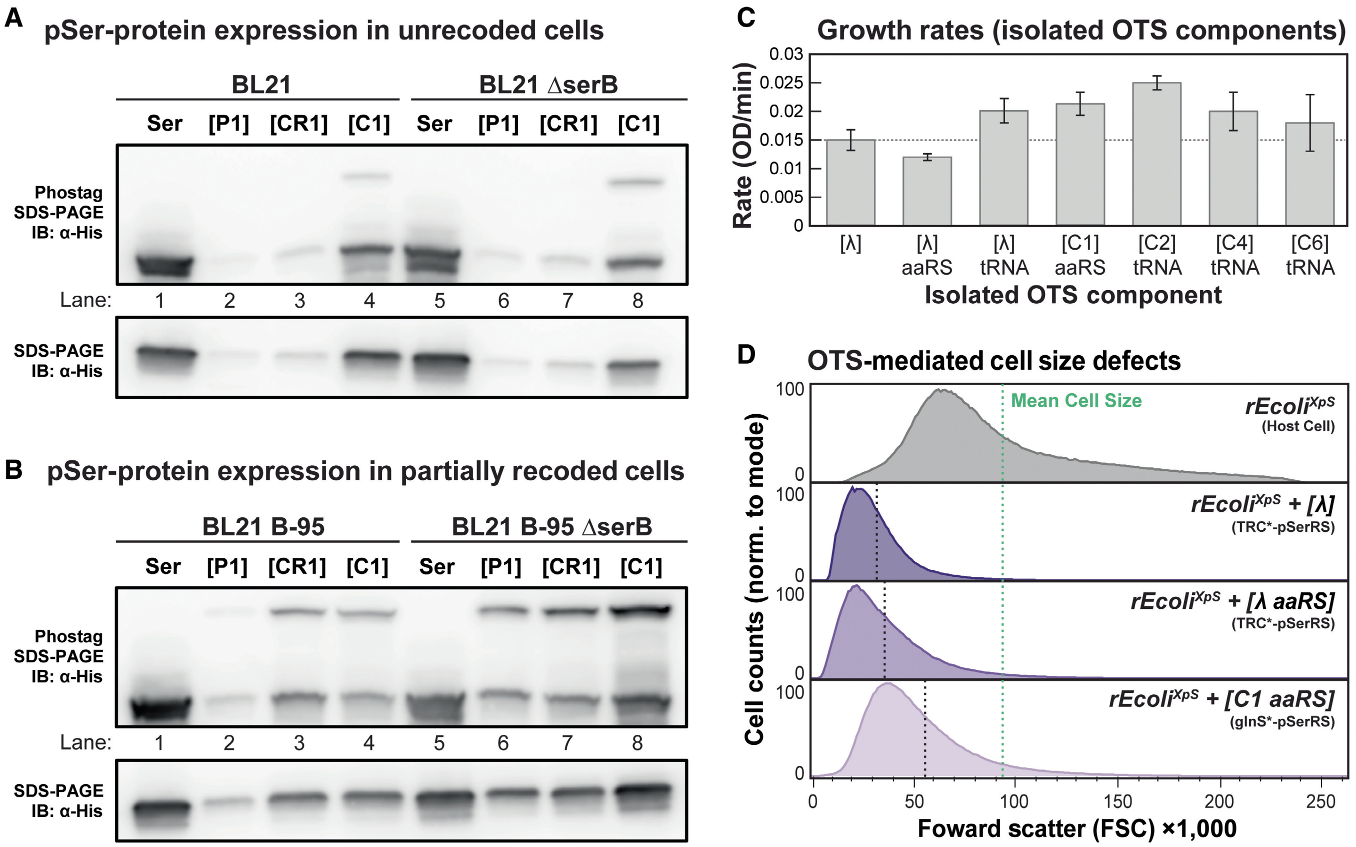

Figure EV1. Genomic recoding status and individual OTS component expression alter OTS performance and cellular fitness.

A–D The phosphoprotein yield and fidelity from non-recoded cells expressing 1× tRNA OTS variants as a function of plasmid copy number (**[P1]**, **[CR1]**, and **[C1]**) were assessed qualitatively by immunoblot analysis of an E(17)TAG-GFP-6×His reporter protein. Crude lysate was separated by SDS–PAGE with and without Phos–tag™ reagent (for phosphoprotein separation) and visualized by immunoblot against the 6xHis epitope tag. Expression and fidelity were assessed in BL21 and BL21 ΔserB (A) and in partially recoded BL21 B-95 and BL21 B-95 ΔserB (B). Each lane was loaded with 7.5 μl of O.D.-normalized cell lysate. Growth rate analysis for host cells (rEcoli^{XpS}) expressing **[λ]** or individual OTS component, as indicated. The growth rate was calculated from continuous monitoring of the optical density (absorbance at 600 nm) as a function of time and averaged across three biological replicates; error bars represent 1 SD, and the horizontal dashed line indicates the average growth rate of cells expressing **[λ]** (C). *E. coli* cell size was measured by flow cytometry with forward light scattering (FSC) used as a proxy for cell size. The size of host rEcoli^{XpS} cells and cells expressing **[λ]** or high-**[λ aaRS]** or low-**[C1 aaRS]** toxicity OTS components were recorded and plotted as histograms representing individual FSC values by abundance in the population. Comparison of population distributions highlights cell size differences across the sample populations. Visualization of cell size distribution and determination of mean cell size (vertical dashed lines) from one million discrete cell measurements was conducted using FlowJo (D).

© 2023 The Authors

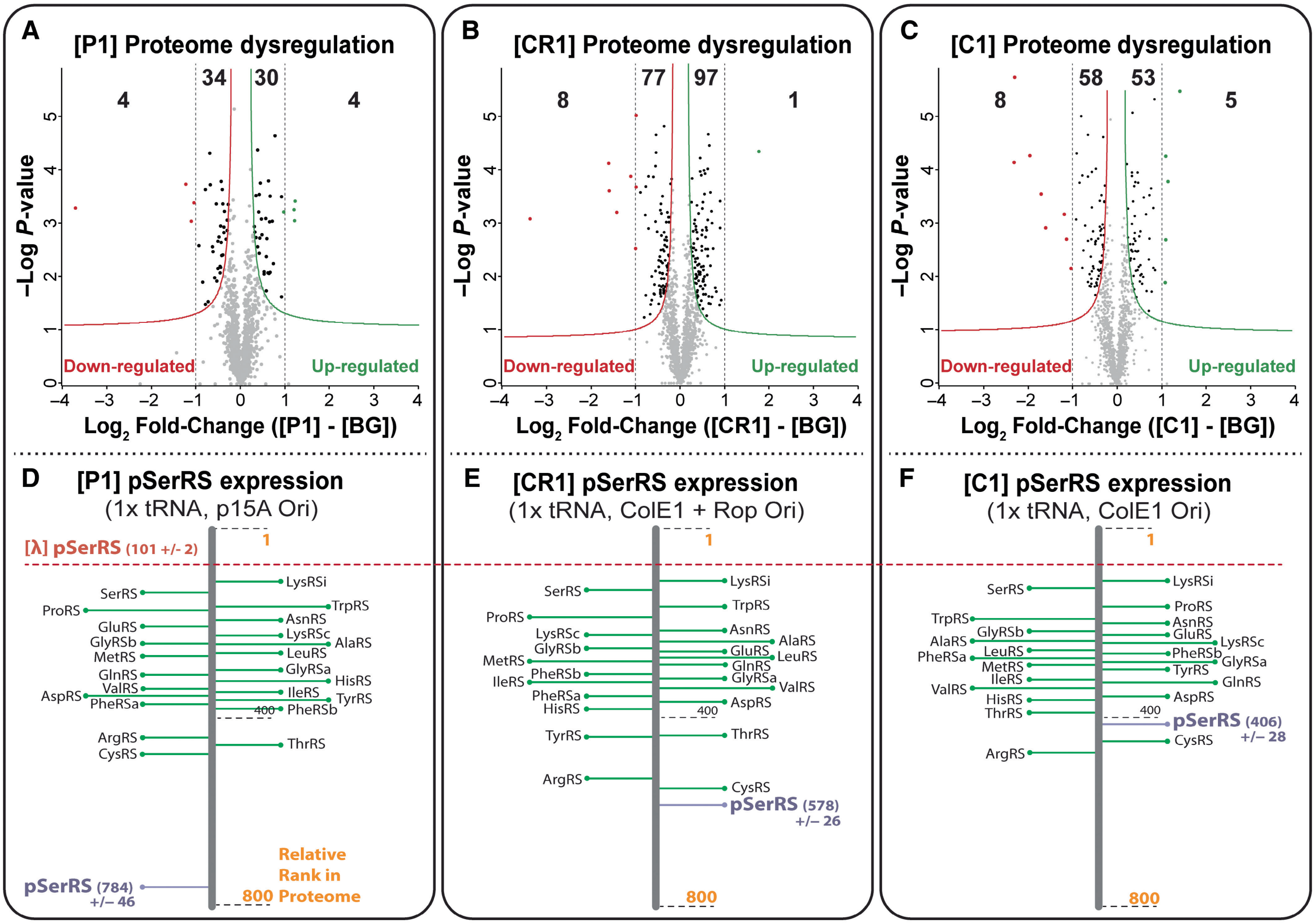

Figure EV2. Plasmid origin of replication contributes to pSerRS-dependent proteome dysregulation.

A–F Proteome composition was determined by mass spectrometry from host cells expressing identical OTS variants aside from their origins of replication; p15a **[P1]** (A), ColE1 + Rop **[CR1]** (B), or ColE1 **[C1]** (C). Proteomic dysregulation and statistical analysis were conducted in Perseus and illustrated as a volcano plot comparing differences in individual protein expression to the transformed *P*-value of the same differentially expressed protein. Statistically significant dysregulated proteins were identified by pair-wise *t*-test ($P < 0.05$) and FDR (0.1) calculation from three independent biological replicates and fell outside the asymptotic lines. From the same proteomes, relative protein abundance was assigned based on rank-ordered, label-free iBAQ protein quantification scores determined by MaxQuant analysis software and used to annotate the abundance of pSerRS (purple line) relative to host *E. coli* aaRSs (green lines) and pSerRS abundance from host cells expressing **[λ]** (red dashed line); p15a **[P1]** (D), ColE1 + Rop **[CR1]** (E), or ColE1 **[C1]** (F).

 © 2023 The Authors

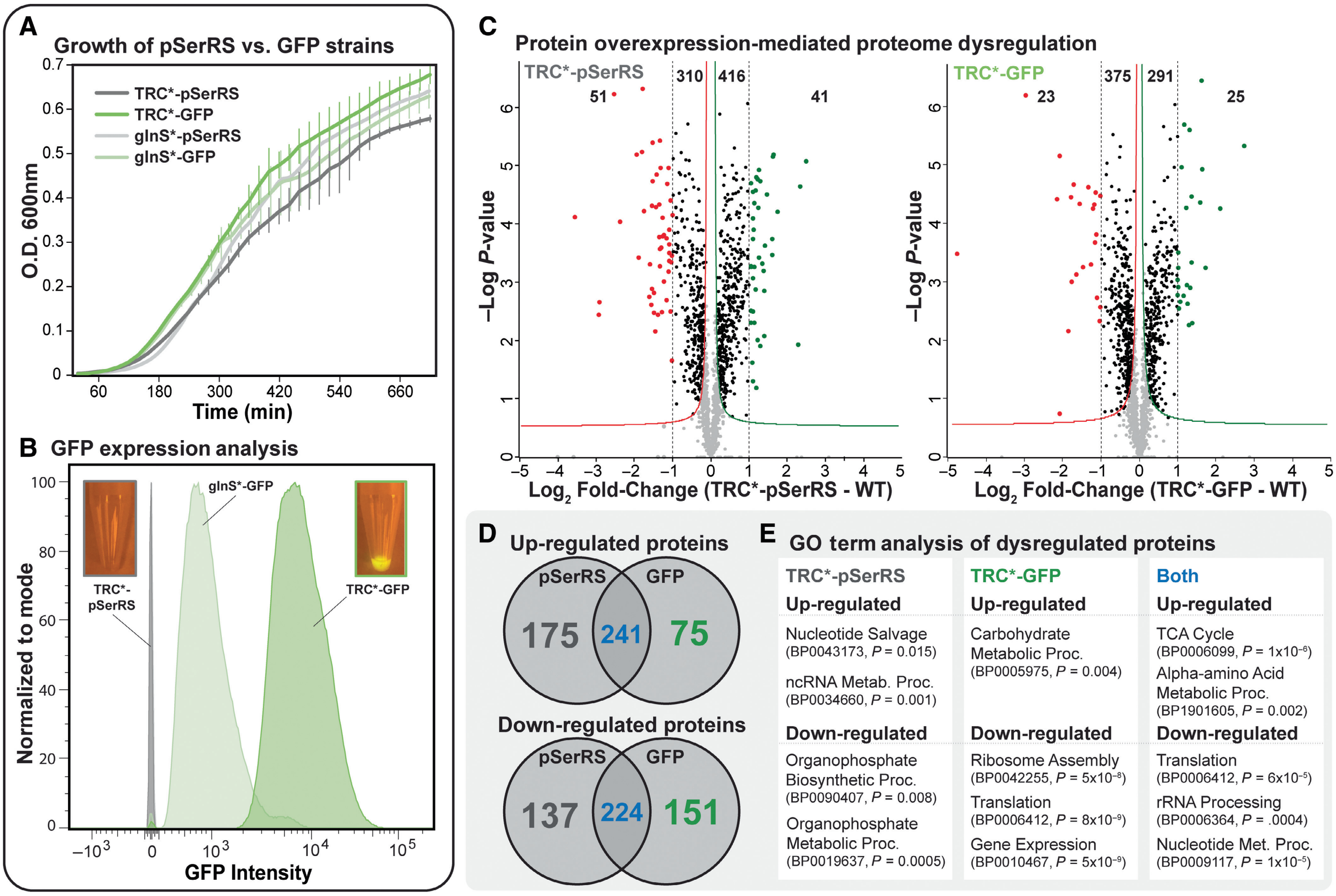

Figure EV3. Identification of pSerRS-specific proteome dysregulation.

A–E Growth fitness defects resulting from overexpression of pSerRS or a non-aaRS protein (GFP) were identified following analysis of kinetic growth metrics derived from continuous monitoring of the optical density (absorbance at 600 nm) as a function of time in host cells with high-level expression of TRC*-pSerRS (dark gray) or TRC*-GFP (dark green) and low-level expression of glnS*-pSerRS (light gray) or glnS*-GFP (light green). Reported values for specific growth rates represent the mean and SD from three biological replicates; growth curve error bars reflect the SD of discrete O.D. measurements (A). Flow cytometry analysis of $1e10^6$ cell to verify GFP expression and intensity across a population of host cells with high-level expression of TRC*-pSerRS (dark gray) or TRC*-GFP (dark green) and low-level expression of glnS*-pSerRS (light gray) or glnS*-GFP (light green). Data analysis and visualization were conducted using FlowJo analysis software and illustrated as an overlay of log scale GFP intensity normalized to individual sample mode (B). Proteome composition was determined by mass spectrometry from host cells expressing either TRC*-pSerRS or TRC*-GFP from identical expression vectors. Proteomic dysregulation and statistical analysis were conducted in Perseus and illustrated as a volcano plot displaying differences in individual protein expression across the pSerRS proteome relative to the proteome of cells expressing GFP. Statistically significant dysregulated proteins were identified by pair-wise *t*-test ($P < 0.05$) and FDR (0.1) calculation from three independent biological replicates and fell outside the asymptotic lines (C). Dysregulated proteins from (C) were compared to identify pSerRS-(gray), GFP-specific (green), and overlapping proteins (blue) relative to the host cell background (D). Extended GO term enrichment analysis of unique and common dysregulated proteins was conducted stringDB and Pathway Tools to identify statistically significant functional pathway and biological process enrichments (*P*-values as indicated) and define the common core response (CCP) to protein overexpression (E). All the data were collected from three independent biological replicated unless otherwise noted.

© 2023 The Authors
