# Supplementary material for: System‐wide optimization of an orthogonal translation system with enhanced biological tolerance
Source: Mol Syst Biol. 2023 Jul 21;19(8):e10591. doi: 10.15252/msb.202110591 (PMC10407733; doi:10.15252/msb.202110591)
Supplement: Supplementary file 5 — PDF+ [file MSB-19-e10591-s004.pdf]

Article

TRANSPARENT PROCESS
OPEN ACCESS

molecular systems biology

System-wide optimization of an orthogonal translation system with enhanced biological tolerance

Kyle Mohler[1,2], Jack M Moen[3,4,5], Svetlana Rogulina[1,2] & Jesse Rinehart[1,2,*]

Abstract

Over the past two decades, synthetic biological systems have revolutionized the study of cellular physiology. The ability to site-specifically incorporate biologically relevant non-standard amino acids using orthogonal translation systems (OTSs) has proven particularly useful, providing unparalleled access to cellular mechanisms modulated by post-translational modifications, such as protein phosphorylation. However, despite significant advances in OTS design and function, the systems-level biology of OTS development and utilization remains underexplored. In this study, we employ a phosphoserine OTS (pSerOTS) as a model to systematically investigate global interactions between OTS components and the cellular environment, aiming to improve OTS performance. Based on this analysis, we design OTS variants to enhance orthogonality by minimizing host process interactions and reducing stress response activation. Our findings advance understanding of system-wide OTS:host interactions, enabling informed design practices that circumvent deleterious interactions with host physiology while improving OTS performance and stability. Furthermore, our study emphasizes the importance of establishing a pipeline for systematically profiling OTS:host interactions to enhance orthogonality and mitigate mechanisms underlying OTS-mediated host toxicity.

Keywords bacterial physiology; orthogonal translation system; phosphoserine; stress tolerance; synthetic biology
Subject Categories Biotechnology & Synthetic Biology; Methods & Resources
DOI 10.15252/msb.202110591 | Received 20 July 2021 | Revised 5 July 2023 | Accepted 5 July 2023 | Published online 21 July 2023
Mol Syst Biol. (2023) 19: e10591

Introduction

Aminoacyl-tRNA synthetases (aaRSs) play a critical role in translating the genetic code into functional proteins. They generate the direct substrates for protein translation by selecting and activating specific amino acids and transferring them to their cognate tRNAs, forming aminoacyl-tRNA (aa-tRNA; Ibba & Soll, 2000; Pang *et al*, 2014). The accuracy of this process, known as aminoacylation, is a primary determinant of proteome fidelity and is maintained through proofreading mechanisms that eliminate mispaired aa-tRNA species (Jakubowski, 2012).

Recent advances in synthetic biology have exploited this fundamental cellular process to expand the genetic code and, thus, the functional space of the proteome by introducing foreign aaRSs and tRNAs that function independently from host translational machinery (Wang *et al*, 2007). This genetic code expansion has led to the development of orthogonal translation systems (OTSs) that enable the incorporation of non-standard and biologically relevant amino acids like phosphoserine (pSer; Park *et al*, 2011) during protein translation using engineered orthogonal aaRS (o-aaRS) and tRNA (o-tRNA) pairs (Liu & Schultz, 2010; Jin *et al*, 2019).

The discovery of an OTS to site-specifically incorporate pSer (pSerOTS) has significantly enhanced our ability to investigate cellular processes involving protein phosphorylation directly (Lee *et al*, 2013; Pirman *et al*, 2015; Rogerson *et al*, 2015; Zhu & Dai, 2019). The pSerOTS uses a phosphoseryl-tRNA synthetase (pSerRS) from *Methanococcus maripaludis* to attach pSer onto a modified *Methanococcus janaschii* tRNA ($tRNA^{pSer}$) that has been re-assigned to decode the UAG stop codon. Subsequent addition of an aa-tRNA-binding translation factor engineered to better accommodate the bulky, negative charge of pSer (EF-pSer) improved delivery of pSer-$tRNA^{pSer}$ to the ribosome and enhanced overall OTS efficiency (Fig 1A; Park *et al*, 2011).

Since their initial deployment, most research efforts have focused on improving OTSs by modifying individual OTS components to increase substrate recognition or catalytic efficiency, yielding general improvements in OTS performance. Despite these improvements, significant challenges remain due to off-target interactions between host translational machinery and OTS components that increase cellular toxicity and compromise OTS function. A significant step toward improving the biological tolerance of OTSs was the

1 Department of Cellular & Molecular Physiology, Yale School of Medicine, New Haven, CT, USA
2 Systems Biology Institute, Yale University, New Haven, CT, USA
3 Quantitative Biosciences Institute (QBI), University of California, San Francisco, San Francisco, CA, USA
4 2QBI Coronavirus Research Group (QCRG), San Francisco, CA, USA
5 Department of Cellular and Molecular Pharmacology, University of California, San Francisco, San Francisco, CA, USA
*Corresponding author. Tel: +1 203 737 3144; E-mail: jesse.rinehart@yale.edu

© 2023 The Authors. Published under the terms of the CC BY 4.0 license

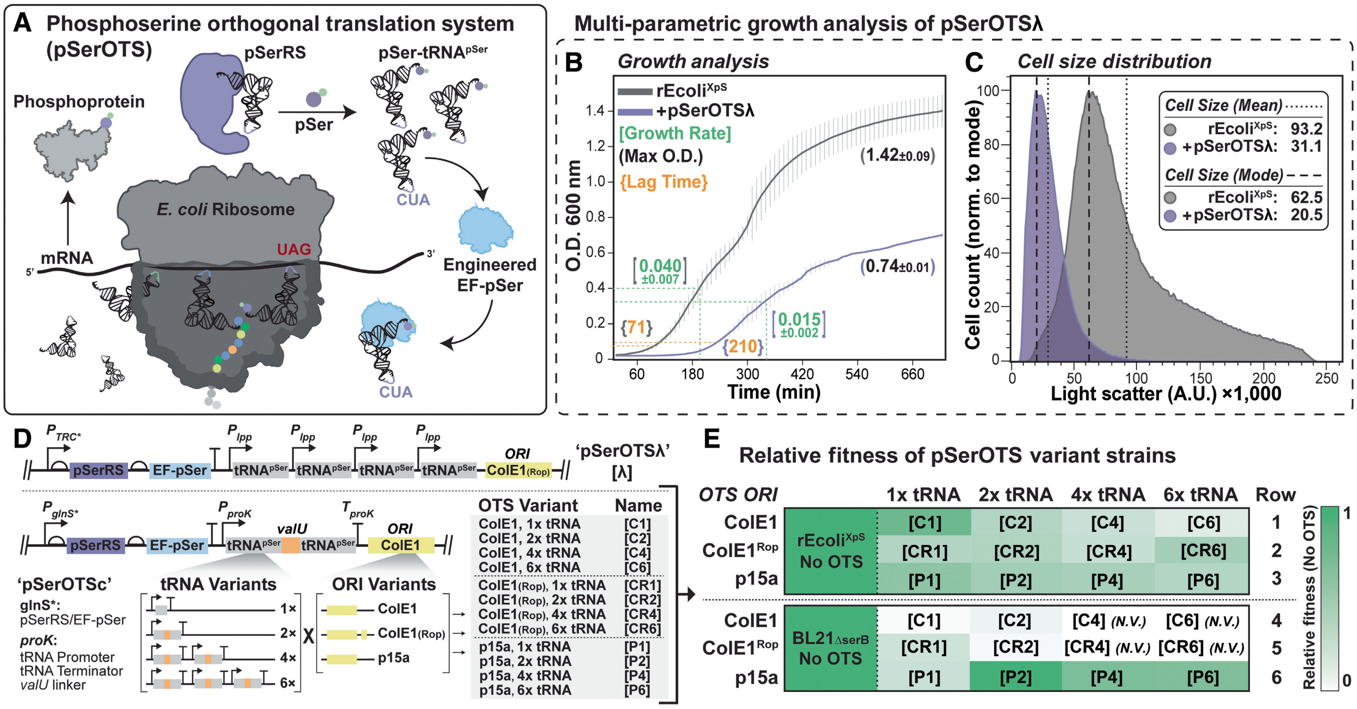

Figure 1. Effects of pSerOTS variant expression on host cell fitness.

A–E Components of the pSerOTS are illustrated in (A). Analysis of kinetic growth metrics derived from continuous monitoring of the optical density (absorbance at 600 nm) as a function of time for pSerOTSλ and host background (rEcoli^{XpS}). Reported values for specific growth rate (green), max O.D. (black), and lag time (orange) represent the mean and SD from three biological replicates; growth curve error bars reflect the SD of discrete O.D. measurements (B). Cell size was determined by proxy using forward light scattering (FSC) measurements from one million individual rEcoli^{XpS} cells with (purple) and without (gray) expression from pSerOTSλ. Cell size distribution, mean, mode, and SD were visualized and calculated using FlowJo analysis software. (C) Variations of the starting OTS architecture **[λ]** include the implementation of glnS* (modified *Ec* GlnRS promotor), proK (*Ec* tRNA^{Pro}) promoter/terminator with valU tRNA linker sequence (*Ec* tRNA^{Val} operon), and plasmid origin of replication (p15a, ColE1, or ColE1 + Rop) (D). The relative fitness of OTS variants (D) in BL21ΔserB and rEcoli^{XpS} host cells was assessed by multi-parametric analysis of kinetic growth metrics derived from continuous monitoring of the optical density (absorbance at 600 nm) as a function of time and cell size measurements. Fitness values were calculated relative to host cell fitness in the absence of an OTS and visualized as a heat map displaying relative fitness with a value of "1," indicating identical fitness relative to the host strain; $n = 3$, detailed parametric data available in Appendix Table S1. *N.V.*, Not Viable (E).

construction of a genetically modified strain of *Escherichia coli* (C321.ΔA, rEcoli; Lajoie *et al*, 2013) where all 321 instances of the TAG stop codon are replaced with TAA, reducing OTS-mediated toxicity caused by global nonsense suppression of UAG stop codons in non-recoded cells (Johnson *et al*, 2011, 2012; Heinemann *et al*, 2012). In addition, deleting RF1, the host release factor responsible for terminating translation at UAG, eliminates protein truncation caused by premature termination in proteins with internal UAG codons (Isaacs *et al*, 2011). While this advancement addresses a fundamental limitation in OTS biology and ultimately enables the permanent reassignment of the TAG codon to a non-standard amino acid, the biological tolerance of many OTSs, including pSerOTS, remains low, and additional sources of OTS-mediated toxicity are poorly understood.

Cells maintain finely tuned pools of metabolites, like amino acids, that serve as substrates for macromolecule production and regulatory agents in metabolic pathways. The amino acid pool is exceptionally dynamic and alters its composition in response to cellular stress to preserve the integrity of protein translation (Sander *et al*, 2019). Artificially disrupting the balance of amino acids in the cell leads to protein mistranslation and misregulation of nutritional stress responses (Starosta *et al*, 2014). Similarly, altering the activity or fidelity of enzymes that interact with the amino acid pool, like aaRSs, misactivates the stringent response (Jakubowski & Goldman, 1992; the central regulator of amino acid biosynthesis) and hinders the cell's ability to accurately sense and respond to amino acid starvation (Bullwinkle & Ibba, 2016; Mohler *et al*, 2017b, 2017c; Kelly *et al*, 2019; Han *et al*, 2020).

The fundamental challenge of OTS biology is engineering OTS components to achieve specificity while maintaining their orthogonality to host translational machinery. Because OTS components interact with cellular substrate pools and, by extension, host cellular processes, they represent a primary source of OTS-mediated cytotoxicity that can be addressed through systematic interrogation of cellular physiology (Ahel *et al*, 2003; Gruic-Sovulj *et al*, 2005; Ling *et al*, 2009; Jakubowski, 2012). However, despite natural precedence indicating that an OTS might interfere with host biological processes, few investigations have focused on characterizing OTS: host interactions or remediating the consequences of these interactions to enhance the biological tolerance of an OTS and improve OTS function. Addressing these concerns should improve OTS-mediated protein yield and fidelity and improve OTS stability.

    © 2023 The Authors

Herein, we use pSerOTS to investigate the intersection of OTS and host physiology to identify specific interactions with host cellular substrates and processes that underlie OTS-mediated cytotoxicity. Through systems-level analysis of host proteomes as a function of OTS composition, we identify dysregulation in stress response pathways and global metabolic burden caused by elements of episomal vectors commonly used in OTS design. Furthermore, by challenging the host cell with individual OTS component expression while monitoring the cellular and molecular phenotypic response, we reveal o-aaRS-specific perturbations in energy metabolism and o-tRNA-dependent reductions in the fidelity of host protein biosynthesis. Together, these findings provide the insight necessary to mitigate toxicity and enable the construction of pSerOTS variants that enhance OTS orthogonality and performance by minimizing off-target interactions within the host cell, thereby improving biological tolerance. Our study presents a generalizable strategy to improve the performance of current and future OTSs by systematically evaluating and resolving deleterious OTS:host interactions and prioritizing biological tolerance during OTS development.

Results

OTS component expression decreases host cell fitness

OTS instability and toxicity negatively impact protein expression in host cells, limiting their widespread use during recombinant protein expression. To improve OTS:compatibility, we aimed to determine the systemic sources of toxicity in a model OTS, pSerOTSλ **[λ]** (Pirman *et al*, 2015). Cellular growth is often distilled to growth rate measurements when, in reality, it represents a composite of discrete growth phases and cellular phenotypes coordinated through complex regulatory schemes (Wick & Egli, 2004; Patange *et al*, 2018; Biselli *et al*, 2020; Hamill *et al*, 2020). Reflecting this complexity, we used a multi-parametric strategy that monitored growth lag time, specific growth rate, growth efficiency (maximum cell density), and cell size distribution to characterize the reproductive fitness of host cells accurately (Akerlund *et al*, 1995; Campos *et al*, 2018; Krishnamurthi *et al*, 2021; Appendix Fig S1). Introducing **[λ]** into rEcoli experimental phosphoserine (rEcoli^{XpS}) host cells caused growth and cell size defects. Compared with rEcoli^{XpS} without an OTS, cells containing **[λ]** displayed a ~2-fold reduction in growth rate and efficiency and a ~3-fold increase in lag time (Fig 1B). In addition, the average cell size (an independent measure of stress) reduced nearly 3-fold as indicated by a reduction in light scattering from 93.2 A.U. to 31.1 A.U. (Fig 1C), which, along with the growth characteristics, is consistent with established responses to cellular stress (Yao *et al*, 2012; Bertrand, 2019; preprint: Miguel *et al*, 2022).

The metabolic burden of heterologous expression systems (including OTSs) on the host cell can negatively impact the expression system's stability and reliability. In the case of OTSs, we theorized that this metabolic burden involves three primary elements that may contribute to OTS-mediated cytotoxicity: the levels of OTS component expression, reduced orthogonality between OTS and host processes, and plasmid maintenance (replication). Plasmid copy number directly influences the degree of metabolic burden in a host cell (Camps, 2010). Most OTS expression vectors utilize the ColE1-family replication origins, which maintain plasmid copy number through a system of small regulatory RNAs and are partially suppressed by accessory repressor proteins (Rops) that reduce steady-state plasmid copy number 3 to 5-fold (Tomizawa & Som, 1984; Lin-Chao *et al*, 1992). To disentangle the OTS-specific metabolic burden from the general metabolic burden imparted by expression system maintenance, we established a spectrum of intracellular OTS plasmid copy numbers – low (p15a), medium (ColE1 + Rop), and high (ColE1; Diaz Ricci & Hernandez, 2000). We constructed a set of rationally designed pSerOTS variants within these expression systems based on a core regulatory framework (pSerOTSc). This framework placed transcriptional control of the *pSerRS-EF-pSer* operon under the constitutive, low-level glnS* promoter and tRNA^{pSer} under the control of an *E. coli* proK tRNA promoter (Chatterjee *et al*, 2013). The names of OTS variants derived from the pSerOTSc framework (outlined in Fig 1D) were designated based on the combination of transcriptional promoters, tRNA copy number, and plasmid ORI (e.g., **[C1]** for pSerOTSc with ColE1 and 1x tRNA).

In addition to the metabolic burden from heterologous expression systems, previous studies demonstrate that fitness costs vary depending on the host genetic background and that fitness defects are particularly severe when plasmid components interact with host cellular processes (Humphrey *et al*, 2012; Standley *et al*, 2019). Therefore, to identify OTS components that mediated genotype-specific reductions in cellular fitness, we chose two modified strains of *E. coli* as host cells: genomically recoded rEcoli^{XpS} and non-recoded BL21ΔserB. In both strains, deleting the phosphoserine phosphatase, *serB*, raised pSer levels in the amino acid pool and eliminated the need for pSer supplementation in growth media (Steinfeld *et al*, 2014).

Using these strains and the new OTS framework, we evaluated the fitness of pSerOTS variants relative to **[λ]** to establish a baseline for experiments to reduce the metabolic burden and improve the biological tolerance of pSerOTS (Fig 1E and Appendix Table S1). Introducing **[λ]** into the non-recoded BL21ΔserB strain was unsuccessful, in line with previously observed toxicity caused by high-level readthrough of UAG stop codons (Lajoie *et al*, 2013). In contrast, **[C1]** was successfully introduced into both BL21ΔserB cells, albeit with reduced fitness, and rEcoli^{XpS} cells, with only a modest reduction in relative fitness (Fig 1E). Under the same conditions, we also examined OTS-mediated E(17)TAG–GFP reporter protein expression to determine whether fitness defects indicated OTS performance. Indeed, immunoblot analysis of reporter protein expression demonstrated that the most OTS variants failed to facilitate pSer incorporation in the BL21ΔserB strain background. However, pSer incorporation was improved in a partially genomically recoded strain of BL21 (BL21ΔserB B-95; Fig EV1A and B), supporting a genotype-dependent relationship between cellular growth and OTS performance (Mukai *et al*, 2015).

Previous work from our lab identified the intracellular concentration of o-tRNA as a critical determinant of OTS fidelity but did not identify the underlying mechanism (Pirman *et al*, 2015). Thus, we aimed to directly investigate this mechanism by constructing three sets of OTSs with varying levels of o-tRNA expression (from one to six copies of tRNA) and plasmid copy number (**[C1]**–**[C6]**, **[CR1]**–**[CR6]**, **[P1]**–**[P6]**, Fig 1D). These OTS variants created a spectrum of intracellular o-tRNA concentration and allowed us to characterize the relationship between o-tRNA abundance, host fitness, and OTS

© 2023 The Authors

performance. To establish this relationship, we first determined the fitness of cells with each tRNA OTS variant in either rEcoli^{XpS} or BL21ΔserB relative to the background strains alone (Fig 1E). In all rEcoli^{XpS} strains, we observed a decrease in relative fitness proportional to the increase in o-tRNA copy number, regardless of the plasmid copy number (Fig 1E, Row 1–3). In contrast, except for lower copy p15a OTS variants, we were unable to generate BL21ΔserB strains with tRNA OTS variants expressing more than two copies of o-tRNA (Fig 1E, Row 4–6), supporting previous observations and establishing a clear role for o-tRNA as a mediator of strain-dependent fitness defects.

Although we observed apparent fitness defects between OTS variants, we hypothesized that each OTS component might also possess the capacity to mediate toxicity independently. To attribute toxicity to specific OTS components, we created variants expressing individual OTS components and monitored fitness defects in rEcoli^{XpS} cells. In comparison to the complete OTS **[λ]**, we observed that host cells expressing a pSerRS-only derivative (**[λ aaRS]**) possessed growth and cell size fitness defects of similar severity (Fig EV1C and D), while, conversely, the relative fitness of cells expressing a tRNA-only derivative (**[λ tRNA]**) was marginally improved relative to **[λ]** (Fig EV1C). In contrast, the fitness of host cells expressing either a glnS*-pSerRS derivative of **[C1]** (**[C1 aaRS]**) or a tRNA-only derivative of **[C2]** (**[C2 tRNA]**) increased relative to **[λ]** (Fig EV1C). Though statistically insignificant, we observed a negative fitness trend following further increases in tRNA-only expression from **[C4 tRNA]** and **[C6 tRNA]** derivatives (Fig EV1C). Our initial characterizations indicate that OTS cytotoxicity results from a mixture of OTS-component-specific mechanisms and strongly correlates with component expression, particularly for o-aaRS. Understanding these discrete mechanisms is critical to improving OTS biological tolerance and performance and warrants further investigation.

aaRS expression levels correlate with host fitness and global proteome dysregulation

We use aggregate cellular phenotypes, such as cell growth or morphology, to identify and evaluate OTS properties that mediate host toxicity. However, these phenotypes lack the necessary resolution to establish causal associations (Caglar *et al*, 2017). To achieve a higher resolution, we adopted a protein-centric molecular phenotyping strategy that pairs comparative shotgun proteomics with label-free quantification to assess system-wide changes in protein abundance (Gouveia *et al*, 2020). Using this strategy, we sought to define the global landscape of OTS:host interactions and identify specific cellular responses mediating OTS toxicity by profiling the composition of host proteomes challenged with OTS expression.

Paralleling our initial investigations of cellular phenotypes, we began by establishing the molecular phenotypes of rEcoli^{XpS} (**[BG]**) cells expressing either high-toxicity **[λ]** or low-toxicity **[C1]** OTS variants. We found that host cells containing **[λ]** had highly dysregulated proteomes (653 proteins), matching its low-relative fitness (Fig 2A). In addition, the relative abundance of pSerRS expressed from **[λ]** was ~2-6-fold higher than the native host aaRS pool, making it one of the most abundant proteins in the proteome (relative abundance of 101 ± 2) and a potentially significant source of metabolic burden (Fig 2B). In comparison, the proteome of cells containing the OTS with the highest relative fitness (**[C1]**) had nearly 5-fold fewer dysregulated proteins (125 proteins, Fig 2C), and the relative abundance of pSerRS was ~4-fold lower, falling within the range of host aaRS pool expression (relative abundance of 406 ± 28, Fig 2D).

We also examined low-toxicity 1× tRNA OTS variants (**[P1]** and **[CR1]**) with similar component compositions but differing origins of replication. When compared with **[C1]**, the low-copy **[P1]** had fewer dysregulated proteins (72 proteins, Fig EV2A) and lower pSerRS levels, falling well below the range of host aaRS pool expression (relative abundance of 784 ± 46, Fig EV2B). Interestingly, medium-copy **[CR1]** had ~50% more dysregulated proteins (183 proteins, Fig EV2C) when compared with high-copy **[C1]**, despite the relative abundance of pSerRS falling predictably between the levels observed in **[P1]** and **[C1]** at 578 ± 26 (Fig EV2D–F). Aside from this inconsistency, pSerRS expression and proteome dysregulation increase proportionally with plasmid copy number.

Previous work has linked plasmid instability to the presence of the Rop plasmid regulatory protein in cells under stress, which helps to explain the proteomic inconsistencies observed in **[CR1]**, as well as the abnormalities in relative fitness observed across CR-based OTS variants (Fig 1E, Row 2; Wegrzyn, 1999; Camps, 2010). As a result, we excluded CR-based OTSs from future analyses. OTSs with p15a origins, despite predictable relative fitness and low-proteomic dysregulation, had a low-relative abundance of pSerRS and, for this reason, were similarly excluded from most follow-up experiments. We focused on the comparative analysis of high-toxicity **[λ]** and low-toxicity **[C1]** OTS variants to dissect the mechanisms of OTS-mediated toxicity.

Overlap analysis of proteomes from cells containing **[λ]** or **[C1]** revealed 319 dysregulated proteins unique to **[λ]**, while only 56 proteins unique to **[C1]**. Furthermore, the intersection of upregulated proteins was lower than that of downregulated proteins, with 26 and 42 shared proteins, respectively (Fig 2E). To better visualize the magnitude and distribution of proteomic dysregulation across host cell processes, we mapped genes corresponding to dysregulated proteins in **[λ]** and **[C1]** onto an illustration of the host genome, revealing genome-wide alteration of metabolic, biosynthetic, and regulatory operons and pathways (Appendix Figs S2 and S3, and Dataset EV1). Correspondingly, pathway enrichment analysis of dysregulated proteins in **[λ]** identified over 60 perturbed host biological processes, including 14 transcription factors and corresponding regulons associated with cellular stress response (e.g., dksA) or nucleotide metabolism (e.g., purR) and few significant pathway perturbations in **[C1]** (Appendix Fig S4 and Dataset EV2).

We next wanted to examine how each OTS component contributed to the **[λ]**-mediated disruption of host pathways. Thus, we examined the molecular phenotypes of cells expressing the isolated components of **[λ aaRS]** and **[λ tRNA]**. Similar to intact **[λ]**, we observed substantial proteomic dysregulation. In cells expressing **[λ aaRS]**, we observed 371 significantly dysregulated proteins (205 up- and 166 downregulated) in contrast to only 196 dysregulated proteins (112 up- and 84 downregulated) in cells expressing **[λ tRNA]** (Fig 2E). Overlap analysis showed that **[λ aaRS]** had the highest overlap of dysregulated proteins with **[λ]** (156 proteins) compared with 77 proteins in **[λ tRNA]** and 68 proteins in **[C1]** (Fig 2E). Consistent with previous observations of cells containing

 © 2023 The Authors

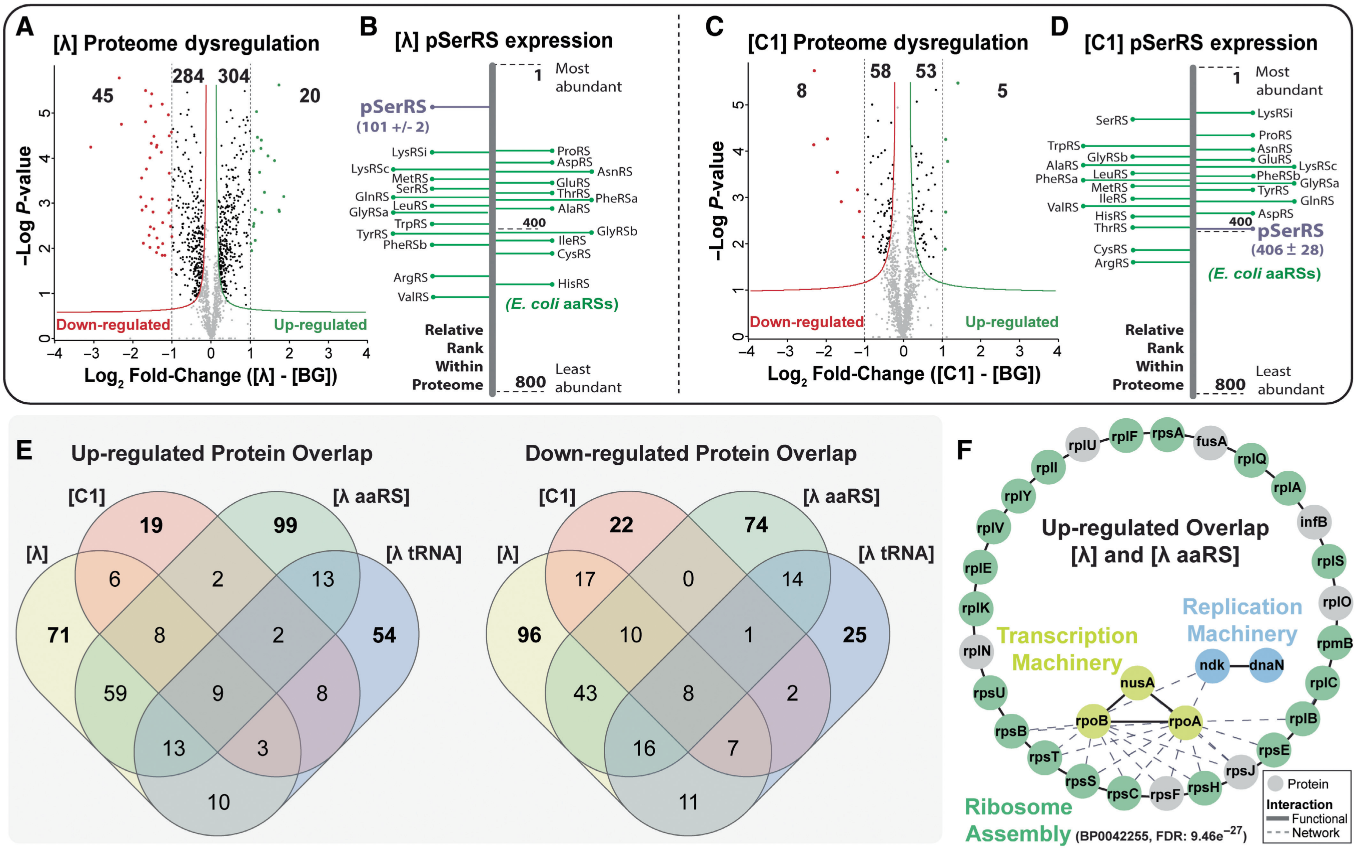

Figure 2. pSerRS-dependent proteome dysregulation and modulation of the metabolic response to stress.

A–F Proteome dysregulation and the relative abundance of pSerRS protein in cells containing various OTS constructs were determined by mass spectrometry. Proteomic composition and statistical analysis were conducted in Perseus and illustrated as a volcano plot comparing differences in individual protein expression to the transformed *P*-value of the same differentially expressed protein. Statistically significant dysregulated proteins were identified by pair-wise *t*-test ($P < 0.05$) and FDR (0.1) calculation from three independent biological replicates and fell outside the asymptotic lines. From the same proteomes, relative protein abundance was assigned based on rank-ordered, label-free iBAQ protein quantification scores determined by MaxQuant analysis software and used to annotate the abundance of pSerRS (purple line) and the level of expression relative to host *E. coli* aaRSs (green lines). Proteome dysregulation and pSerRS abundance were determined for high-toxicity (**[λ]** A, B) and low-toxicity (**[C1]** C, D) OTS variants. The overlap of significantly upregulated and downregulated proteins from host cells expressing full OTS or OTS components was assessed following mass spectrometry, and statistical analysis using Perseus is summarized in (E). The overlap of significantly up-regulated proteins from host cells expressing **[λ]** or **[λ aaRS]** was analyzed using stringDB to identify statistically significant pathway enrichments and grouped using a k-means clustering algorithm. Functionally enriched interactions were observed for ribosome assembly (green), transcription (yellow), and DNA replication (blue) processes (F).

excess uncharged tRNA, isolated o-tRNA expression from **[λ tRNA]** caused upregulation of proteins involved in amino acid biosynthesis, which suggests that the accumulation of uncharged o-tRNA activates the stringent response (Dworkin, 2023; Appendix Fig S5).

GO term enrichment analysis of **[λ]** and **[λ aaRS]** overlap uncovered a functionally enriched protein network composed primarily of proteins involved in ribosome assembly (Fig 2F, FDR: 9.46e-27). Considering also the overlap with proteins dysregulated in **[λ tRNA]** and **[C1]**, expression from **[λ aaRS]** resulted in the highest number of unique protein dysregulations (173 proteins) and, astonishingly, accounted for nearly 50% of total protein dysregulation across all conditions (Fig 2E). While these observations implicate high-level o-aaRS expression as a primary mediator of OTS cytotoxicity, the question remains whether this toxicity results from generic protein overexpression stress or the enzymatic properties of o-aaRSs.

High-level orthogonal aaRS expression redirects cellular energy resources and reduces host aa-tRNA pool fidelity

Heterologous protein overexpression can be toxic to the host cell, but the unique properties of the overexpressed protein may also be a contributing factor. Metabolic burden during protein overexpression activates a cellular stress response that resembles the heat shock response and amino acid limitation, reducing the expression of both the heterologous and host cell proteins (Pramanik & Keasling, 1997; Gill *et al*, 2000; Durrschmid *et al*, 2008; Camps, 2010). The overexpression of an aaRS presents additional challenges due to its catalytic activity and interaction with numerous host substrate pools and, in some instances, causes toxicity (Bedouelle *et al*, 1990; Chalker *et al*, 1994). To determine whether the toxicity observed during high-level pSerRS expression is generic or pSerRS-specific, we examined the cellular and molecular

phenotypes of host cells expressing either pSerRS or a control protein, GFP, from vectors with identical regulatory elements.

High-level expression of pSerRS driven by the TRC* promoter (TRC*-pSerRS) reduced host fitness relative to cells expressing GFP from the same promoter (TRC*–GFP, Fig 3A). However, we observed no fitness defects when pSerRS and GFP were expressed from a low-level promoter (glnS*) or between cells expressing either high- or low-level GFP (Fig EV3A and B). Comparative proteomics of cells expressing TRC*-pSerRS and -GFP revealed highly dysregulated proteomes in both strains, with 818 and 714 significantly dysregulated proteins, respectively (Fig EV3C). We analyzed the overlap of these dysregulated proteins and identified a core set of over 450 proteins shared between cells expressing either pSerRS or GFP (Fig 3B). Enriched biological processes from GO term analysis of overlapping "common core" proteins (CCP) mirrored previously characterized molecular phenotypes related to ColE1-based episomal vectors (TCA cycle upregulation) and protein overexpression (stringent response activation), comprising the core cellular response to heterologous protein expression (Fig 3C; Wang *et al*, 2006; Durrschmid *et al*, 2008).

After removing common core proteins, we found over 300 uniquely dysregulated proteins in host cells with high-level pSerRS expression and that these dysregulated proteins are enriched for nucleotide salvage and organophosphate metabolic processes (Figs 3B, and EV3D and E). In contrast, we observed few significantly enriched processes among the 225 proteins uniquely dysregulated during high-level GFP aside from processes associated with common core stress response pathways, suggesting that the unique molecular phenotype during pSerRS expression mediates the reduction in relative fitness (Fig EV3E). Supporting this conclusion, a direct comparative analysis of molecular phenotypes from TRC*-pSerRS and -GFP strains found 341 upregulated and 231 down-regulated proteins associated with pSerRS expression that could participate in aaRS-mediated cytotoxicity (Fig 3D).

As key enzymes in protein translation, aaRSs interact with numerous cellular substrate pools, providing ample opportunity to disrupt host processes. Like host aaRSs, o-aaRSs consume ATP to activate amino acids. ATP concentration is tightly regulated by monitoring the composition of the intracellular adenylate pool (i.e., ATP, ADP, and AMP), and excessive ATP consumption can disrupt adenylate pool homeostasis leading to compromised growth (Atkinson, 1977). High-level pSerRS expression may facilitate excessive ATP consumption via non-productive aminoacylation by failing to transfer the activated pSer–AMP to its tRNA acceptor. The unused pSer–AMP could either diffuse from the active site or (as in the closest structural homolog to pSerRS, archaeal PheRS (Kamtekar *et al*, 2007; Englert *et al*, 2013)) hydrolyze pSer–AMP to release pSer and AMP (Fig 3E; Rauhut *et al*, 1985; Gruic-Sovulj *et al*, 2007). In support of this model, we found that the enzymes responsible for degrading AMP (Amn) and pyrophosphate (Ppa) are upregulated during high-level pSerRS expression (TRC*-pSerRS and **[λ]**) and remain upregulated after removal of CCP, i.e., (pSerRS–GFP), but are not significantly upregulated during low-level pSerRS expression from **[C1]** (Fig 3F). Furthermore, enzymes central to the adenine

Figure 3. Sources of aaRS-mediated cytotoxicity.

A–M Growth fitness defects resulting from overexpression of pSerRS or a non-aaRS protein (GFP) were identified following analysis of kinetic growth metrics derived from continuous monitoring of the optical density (absorbance at 600 nm) as a function of time in host cells with high-level expression of TRC*-pSerRS (gray) or TRC*-GFP (green). Reported values for specific growth rates represent the mean and S.D. from three biological replicates; growth curve error bars reflect the SD of discrete O.D. measurements (A). The molecular phenotype of the same samples with either high-level expression of TRC*-pSerRS (gray) or TRC*-GFP (green) relative to the host cell proteome without protein overexpression was assessed by mass-spectrometry comparative proteomics. Statistically significant dysregulated proteins from three biological replicates were identified in Perseus by pair-wise *t*-test ($P < 0.05$) and FDR (0.1) calculation. Dysregulated proteins from both overexpression conditions were compared to identify unique and overlapping dysregulated proteins relative to the host cell background (B). Dysregulated protein overlap was analyzed in stringDB to identify statistically significant functional pathway and process enrichments that define the general common core response (CCP) to protein overexpression (C). Direct comparison of proteomic dysregulation between cells expressing TRC*-pSerRS (pSerRS) or TRC*-GFP (GFP) was conducted in Perseus and illustrated as a volcano plot displaying differences in individual protein expression across the pSerRS proteome relative to the proteome of cells expressing GFP, i.e. (pSerRS–GFP). Statistically significant dysregulated proteins were identified by pair-wise *t*-test and FDR calculation from three independent biological replicates and fall outside the asymptotic lines (D). There are several potential mechanisms of aaRS-mediated cytotoxicity. Excessive ATP consumption through non-productive aminoacylation and diffusion or hydrolysis of the activated amino acid from the active site of pSerRS (E). Pathway enrichment analysis of dysregulated proteins identified through proteomic analysis of cells expressing TRC*-pSerRS, **[λ]**, **[C1]**, and the differential expression of (pSerRS–GFP) from D was conducted using Pathway Tools analysis. Significantly enriched pathways and the expression of pathway constituents are for AMP and pyrophosphate (PPi) degradation (F) and the adenine salvage pathway (G) are displayed with upregulated proteins in red, downregulated proteins in purple, and proteins with no change in expression compared to background host cell proteomes in gray. Enrichment cutoffs were set to a statistically significant differential expression score of 0.1. Excessive ATP consumption may also occur through the synthesis of Diadenosine Tetraphosphate (Ap_4A) or other dinucleotide phosphates in the active site of pSerRS through interaction with additional ATP molecules during non-productive aminoacylation (H). Pathway enrichment analysis of dysregulated proteins using proteomic data and analysis methods described above for E–G revealed additional pathway enrichments for enzymes involved in the degradation of Ap_4A and conversion of ADP to ATP (I). aaRS-mediated cytotoxicity may also occur through aberrant interactions with host tRNAs that result in the misincorporation of pSer and protein mistranslation. tRNA misrecognition by pSerRS occurs due to overlapping tRNA identity elements with tRNAs. This overlap is illustrated by the comparison of o-tRNA^{pSer} and *E. coli* tRNA^{Gly} acceptor stem sequences displaying primary sequence overlap (gray circles), tRNA^{Gly} specific identity elements (blue circles), and identity elements common to both tRNAs (green circles) (J). Host tRNA misrecognition and decoding fidelity at Gly, Ser, and Thr sense codons was assessed by monitoring site-specific amino acid incorporation at the indicated position within the MS–READ reporter peptide (red circle marked with X in peptide). Incorporation events were identified by mass spectrometry and qualitatively assessed by integration of the area under the curve for MS1 precursor ion intensities using Skyline, with faithful decoding (green bars), misincorporation (gray bars), and pSer misincorporation (purple bars) displayed according to relative peptide intensity for each reporter codon (K). After proteomic analysis and removal of common core proteins (CCP), the overlap of remaining dysregulated proteins was assessed for **[λ]** and TRC*-pSerRS (L) or cells expressing o-tRNA only **[λ tRNA]** (M). Overlapping proteins were input into stringDB to construct network diagrams and identify statistically significant functional protein associations. Functional associations were further distinguished by biological process and molecular function enrichments indicated by the colored nodes and associated FDR value, with edge width indicating the confidence of association. Colored rings around nodes indicate the upregulation (red) or downregulation (purple) of each protein node within the source proteome.

© 2023 The Authors

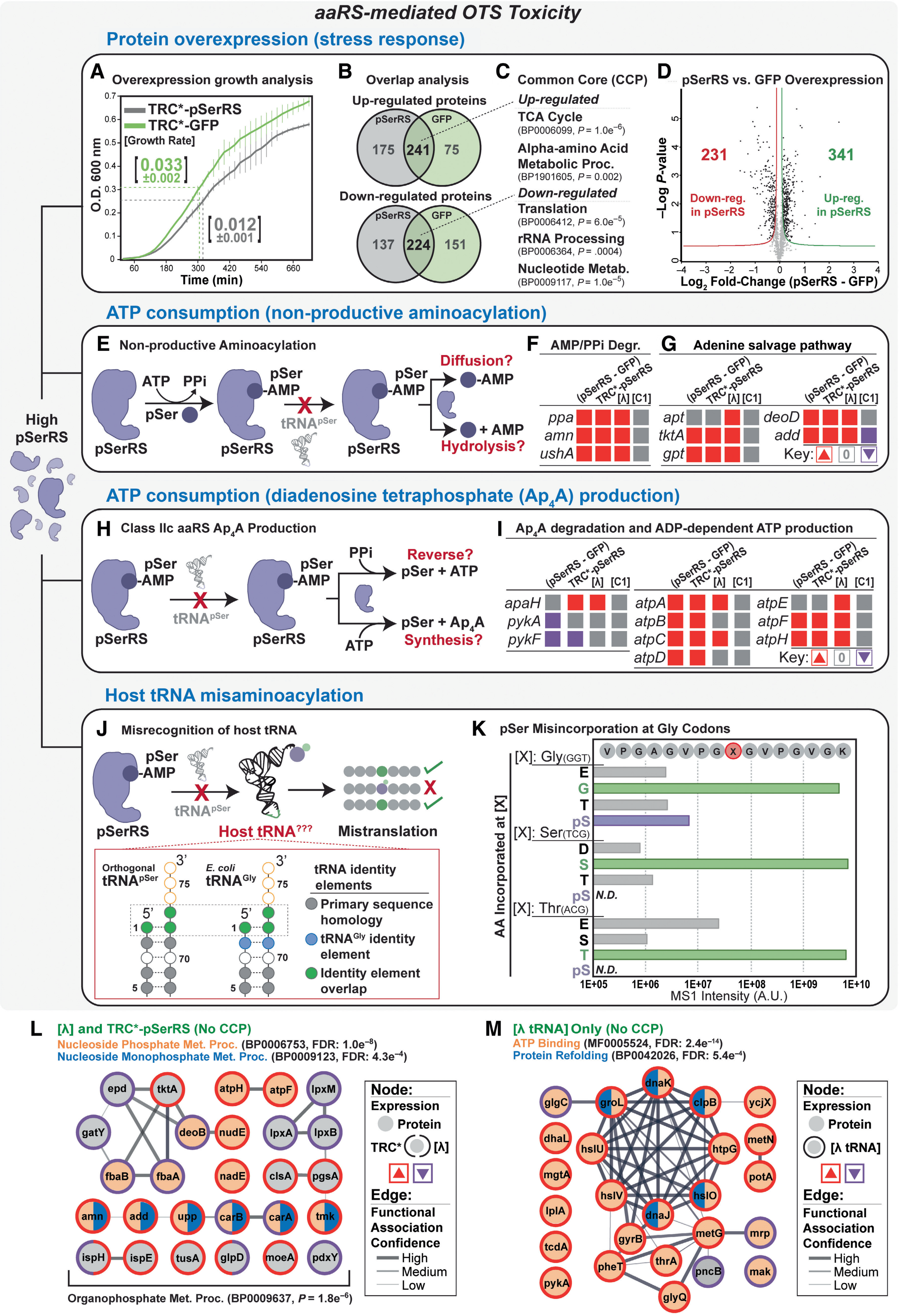
aaRS-mediated OTS Toxicity
Protein overexpression (stress response)
A Overexpression growth analysis
TRC*-pSerRS
TRC*-GFP
[Growth Rate]
0.033 ±0.002
0.012 ±0.001
O.D. 600 nm
Time (min)
B Overlap analysis
Up-regulated proteins
pSerRS GFP
175 241 75
Down-regulated proteins
137 224 151
C Common Core (CCP)
Up-regulated
TCA Cycle
(BP0006099, P = 1.0e−6)
Alpha-amino Acid Metabolic Proc.
(BP1901605, P = 0.002)
Down-regulated
Translation
(BP0006412, P = 6.0e−5)
rRNA Processing
(BP0006364, P = .0004)
Nucleotide Metab.
(BP0009117, P = 1.0e−5)
D pSerRS vs. GFP Overexpression
231
341
Down-reg. in pSerRS
Up-reg. in pSerRS
−Log P-value
Log2 Fold-Change (pSerRS - GFP)
ATP consumption (non-productive aminoacylation)
E Non-productive Aminoacylation
ATP PPi
pSer
pSerRS
pSer -AMP
tRNApSer
Diffusion?
-AMP
+ AMP
Hydrolysis?
F AMP/PPi Degr.
(pSerRS - GFP) TRC*-pSerRS [λ] [C1]
ppa
amn
ushA
G Adenine salvage pathway
apt
tktA
gpt
deoD
add
Key:
High pSerRS
ATP consumption (diadenosine tetraphosphate (Ap4A) production)
H Class IIc aaRS Ap4A Production
pSer -AMP
tRNApSer
pSerRS
PPi
Reverse?
pSer + ATP
pSer + Ap4A
Synthesis?
ATP
I Ap4A degradation and ADP-dependent ATP production
apaH
pykA
pykF
atpA
atpB
atpC
atpD
atpE
atpF
atpH
Key:
Host tRNA misaminoacylation
J Misrecognition of host tRNA
pSer -AMP
tRNApSer
pSerRS
Host tRNA???
Mistranslation
Orthogonal tRNApSer
E. coli tRNAGly
3' 5' 75 70 1 5
tRNA identity elements
Primary sequence homology
tRNAGly identity element
Identity element overlap
K pSer Misincorporation at Gly Codons
V P G A G V P G X G V P G V G K
[X]: Gly(GGT)
[X]: Ser(TCG)
[X]: Thr(ACG)
E G T pS D S T pS E S T pS
N.D.
AA Incorporated at [X]
MS1 Intensity (A.U.)
L [λ] and TRC*-pSerRS (No CCP)
Nucleoside Phosphate Met. Proc. (BP0006753, FDR: 1.0e−8)
Nucleoside Monophosphate Met. Proc. (BP0009123, FDR: 4.3e−4)
epd tktA atpH atpF lpxM gatY deoB nudE lpxA lpxB fbaB fbaA nadE clsA pgsA amn add upp carB carA tmk ispH ispE tusA glpD moeA pdxY
Organophosphate Met. Proc. (BP0009637, P = 1.8e−6)
Node: Expression Protein TRC* [λ]
Edge: Functional Association Confidence High Medium Low
M [λ tRNA] Only (No CCP)
ATP Binding (MF0005524, FDR: 2.4e−14)
Protein Refolding (BP0042026, FDR: 5.4e−4)
dnaK glgC groL clpB ycjX dhaL hslU htpG metN mgtA potA lplA hslV hslO dnaJ tcdA gyrB metG mrp pheT thrA pncB mak pykA glyQ
Node: Expression Protein [λ tRNA]
Edge: Functional Association Confidence High Medium Low

Figure 3.

salvage pathway and nucleotide biosynthesis (including those that scavenge the byproducts of AMP degradation by Amn) followed the same expression pattern, consistent with previously characterized mechanisms of adenylate pool maintenance through synthesis and degradation of AMP (Fig 3G and Appendix Fig S6; Atkinson, 1977; Ataullakhanov & Vitvitsky, 2002).

In addition, pSerRS may intensify its ATP consumption if it uses multiple ATP molecules to produce the stress-induced signaling molecule diadenosine tetraphosphate (Ap_4A; Lee *et al*, 1983; Ji *et al*, 2019). If non-productive pSer–AMPs are not hydrolyzed or reverted to free pSer and ATP through the reverse activation reaction, the pSer–AMP bound in the active site, similar to other class II aaRSs, can substitute a second ATP molecule for pSer and produce Ap_4A (ATP + AMP, Fig 3H; Zamecnik *et al*, 1966; Belrhali *et al*, 1995; Chen *et al*, 2017). As we would expect to see in response to this model of Ap_4A accumulation, we observe that the only enzyme known to degrade Ap_4A in *E. coli*, the ApaH phosphatase, is up-regulated during high-level pSerRS expression from TRC*-pSerRS and **[λ]** and remains up-regulated after removal of CCP i.e., (pSerRS – GFP), but is not significantly up-regulated during low-level pSerRS expression from **[C1]** (Fig 3I) (Farr *et al*, 1989). Further supporting this model, we find that the proteins comprising ADP-dependent ATP-synthase (AtpA-H) that are typically down-regulated during protein overexpression are up-regulated during high-level pSerRS expression (Durrschmid *et al*, 2008; Fig 3I). The additional down-regulation of enzymes that consume AMP (PykA and PykF) indicates that excess ADP generated during Ap_4A cleavage may imbalance the adenylate pool and that the increase in ATP-synthase expression re-stabilizes this pool by matching ADP consumption with production (Fig 3I).

Beyond ATP consumption, o-aaRS-mediated cytotoxicity could reflect proteome damage caused by off-target interactions between pSerRS and host tRNAs. Prior work demonstrated that altering aaRS:tRNA ratios by overexpressing an aaRS reduced its selectivity for tRNAs and compromised aaRS fidelity (Swanson *et al*, 1988; Sherman *et al*, 1992). In addition, maintaining tRNA orthogonality is challenging because of limited opportunities for structural and sequence variations (i.e., identity elements), and any overlap between host and o-tRNA identity elements can exacerbate the misrecognition of tRNA during o-aaRS overexpression. In the context of these observations, overexpression of pSerRS, particularly in the absence of its $tRNA^{pSer}$, is likely to reduce its ability to discriminate against host tRNAs efficiently and lead to a global reduction in host proteome fidelity, and thus host fitness, through pSer misincorporation (Fig 3J).

Using available literature, we identified host tRNAs with identity elements recognized by pSerRS and found significant identity element overlap with two host tRNAs: $tRNA^{Gly}$ and $tRNA^{Thr}$ (Hasegawa *et al*, 1992; Nameki *et al*, 1997). To assess whether pSerRS could recognize these tRNAs and cause pSer misincorporation, we used our previously established mass spectrometry reporter for exact amino acid decoding (MS–READ), which quantifies amino acid incorporation at a specific codon within the reporter peptide to detect pSer incorporation at Gly, Thr, or a control Ser codon (Mohler *et al*, 2017a). During high-level pSerRS expression, we found pSer misincorporation exclusively at Gly codons (Fig 3K and Appendix Fig S7). This observation confirms that the extensive identity element homology within the tRNA acceptor stem enables $tRNA^{Gly}$ misrecognition and pSer misincorporation (Fig 3J and K, and Appendix Fig S8A and B). Based on this result, we re-examined our proteomic data for pSer misincorporation events at native Gly codons across the host proteome and identified 33 high-confidence Gly to pSer substitution events during high-level pSerRS expression from **[λ aaRS]**. In striking contrast, we observed zero misincorporation events in the proteomes of either background host cells or cells during low-level pSerRS expression (Appendix Table S2). In addition, LC–MS/MS analysis of a candidate mistranslated peptide reproducibly observed across all samples at Gly110 of the host protein groL confirmed pSer misincorporation (Appendix Fig S8C).

All of the mechanisms of aaRS-mediated cytotoxicity outlined above likely contribute to reductions in host fitness, but the primary contributor appears to be ATP consumption. After removing proteins that fall into the common core response and mask pSerRS-specific molecular phenotypes, most of the remaining dysregulated proteins observed during high-level pSerRS expression are associated with stabilizing the host cell's adenylate pool. Supporting this conclusion, we were able to establish functional network enrichments from the remaining dysregulated proteins unique to both **[λ aaRS]** and **[λ]** with significant enrichments for organophosphate and nucleoside monophosphate metabolic processes (Fig 3L, Network *P*-value = 1.8e–6). Similar network analysis of **[λ tRNA]**-specific proteins displayed unique functional enrichment for proteins that mediate protein refolding (Fig 3M, BP FDR: 5.4e-16). Importantly, this observation raises the question of the extent to which o-tRNA independently contributes to reductions in OTS performance and biological tolerance.

Balance of orthogonal tRNA in the tRNA pool is essential to translational fidelity

Foreign o-tRNAs introduced into a host cell can interact with cellular translational and regulatory processes, leading to toxicity. Despite the opportunity for these interactions, they are seldom evaluated as discrete components during o-tRNA development, leaving their contributions to OTS fidelity and host fitness poorly characterized. During our initial observations of host fitness, we noted that relative fitness was inversely proportional to o-tRNA copy number (Fig 1E, Row 1). More specifically, we observed an ~8-fold reduction in growth rate as the o-tRNA copy number increased from one copy of $tRNA^{pSer}$ (**[C1]**) to six copies (**[C6]**) which, strikingly, corresponded to a ~3-fold reduction in growth rate relative to **[λ]** (Fig 4A). Although less pronounced, we observed a similar trend across P1-variants as o-tRNA copy number was increased (Fig 1E, Row 3).

In addition to reducing host fitness, the overexpression of o-tRNA can compromise OTS fidelity. As an initial qualitative assessment of OTS fidelity, we expressed an E17(TAG)–GFP reporter protein alongside o-tRNA-copy-number-variants and monitored the relative incorporation of pSer following resolution by PhosTag SDS–PAGE. We observed a dose-dependent increase in the fraction of non-pSer-containing protein as the o-tRNA copy number was increased from one copy to six copies of $tRNA^{pSer}$, while the abundance of pSer-containing protein remained unchanged across the same series (Fig 4B, Lanes 1–4). Notably, aggregate reporter expression as assessed by SDS–PAGE was relatively similar among o-tRNA variants and, in all cases, higher than reporter expression mediated

 © 2023 The Authors

by **[λ]** (Fig 4B, Lanes 1–5). We obtained similar results from o-tRNA-copy-number P1 variants (Appendix Fig S9A and B).

The o-tRNA-dependent reductions in host fitness and OTS fidelity could result from three primary mechanisms: through (i) misdecoding (ribosome selecting the wrong tRNA), through (ii) misaminoacylation (host aaRSs improperly selecting $tRNA^{pSer}$), or through (iii) misactivation (pSerRS activating the wrong amino acid; Fig 4C). Thus, we aimed to establish the specific contribution and the relative extent of these mechanisms toward o-tRNA-mediated cytotoxicity.

Misdecoding at UAG stop codons (i.e., nonsense suppression) occurs naturally during protein translation when a ribosome selects an aa-tRNA instead of terminating translation or, occasionally, when mutations in a tRNA allow it to recognize and decode UAG codons (Fig 4Ci; Biswas & Gorini, 1972; Lu *et al*, 1995; Singaravelan *et al*, 2010; Manickam *et al*, 2014; Roy *et al*, 2015). To establish a baseline for UAG misdecoding, we profiled amino acid incorporation at UAG by MS–READ using protein purified from **[BG]** cells (no OTS) and identified five unique UAG misdecoding events. In addition, we quantified reporter peptide intensity and found that the primary misdecoding events were Phe, Gln, and Tyr. We also observed the incorporation of Lys, though an order of magnitude lower in intensity (Fig 4D). Except for Phe, these data are consistent with previous observations and establish the baseline contribution of UAG misdecoding to protein mistranslation mediated by the native tRNA pool in **[BG]** cells (Lajoie *et al*, 2013; Aerni *et al*, 2015; Gan & Fan, 2017).

In addition to misdecoding by host tRNAs, the expression of an o-tRNA that shares identity elements with one or more host tRNAs results in misrecognition by host aaRSs (misaminoacylation) and, if uncorrected, protein mistranslation (Fig 4Cii). The recognition of tRNA identity elements and the relative affinity for a given tRNA among competing aaRSs are the primary determinants of accurate tRNA selection (Sherman *et al*, 1992). As such, the fidelity of this

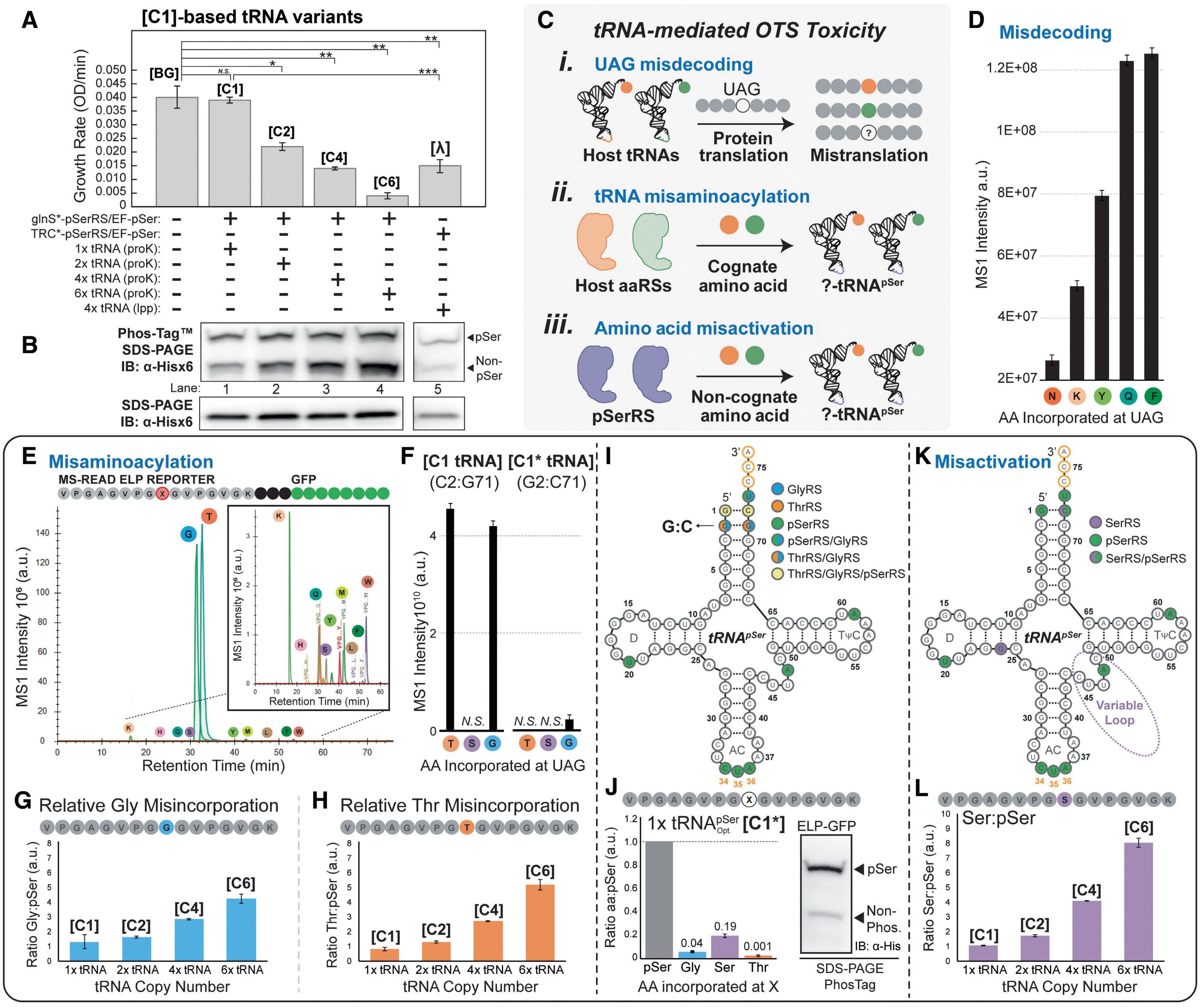

Figure 4.

◀ **Figure 4. tRNA identity element overlap facilitates concentration-dependent o-tRNA-mediated cytotoxicity.**

A–L Cytotoxicity resulting from o-tRNA expression was assessed by comparative analysis of cellular growth phenotype from host cells **[BG]** expression [C1]-based OTS variants (**[C1]–[C6]**) or **[λ]**. All the growth rate parameters reflect the average measurements from three independent biological replicates with error bars representing 1 SD Statistical significance of growth rate changes was assessed by two-tailed student's *t*-test (equal variance) where * equals $P < 0.05$, ** equals $P < 0.005$, *** equals $P < 0.0005$, and *N.S.* denotes Not Significant (A). Preliminary qualitative assessment of OTS fidelity as a function of o-tRNA expression (corresponding to experimental conditions in (A)) was conducted by monitoring the relative incorporation of pSer into a reporter protein separated by either Phos–Tag™ SDS–PAGE (indicated upper band = pSer and indicated lower band = non-pSer) or standard SDS–PAGE and visualized by immunoblot against the 6×HIS-tagged reporter protein. Each lane was loaded with 7.5 μl of O.D.-normalized cell lysate (B). Potential modes of tRNA-mediated OTS toxicity include misdecoding of UAG codons via host tRNA nonsense suppression events (i), Misaminoacylation of o-tRNA with non-cognate amino acid by host aaRSs (ii), and misactivation of non-cognate amino acids by the o-aaRS (iii) (C). o-tRNA-mediated toxicity from misdecoding (i) was assessed by mass spectrometry through the identification of amino acid incorporation events in a UAG-containing MS–READ reporter protein expressed in host cells without an OTS. Amino acid incorporation (for indicated amino acids) was quantified in Skyline using MS1 precursor ion intensities (area under the curve) averaged across three biological replicates and displayed as a function of amino acid identity; error bars represent 1 SD (D). Misaminoacylation of o-tRNA by host aaRSs (ii) was identified by analysis of amino acid incorporation events in host cells expressing a UAG-containing MS–READ reporter protein alongside $tRNA^{pSer}$ alone. Amino acid incorporation events were identified by mass spectrometry and analyzed using Skyline. An example output displaying MS1 ion intensity as a function of chromatographic peptide retention time illustrates the resolution of the reporter peptides with single amino acid substitutions which enables MS–READ-based amino acid incorporation analyses (E). Select amino acid incorporation events from (E) were quantified by integration of MS1 precursor ion intensities (area under the curve) for both unmodified and optimized $tRNA^{pSer}$ averaged across three biological replicates and displayed as a function of amino acid identity; error bars represent 1 SD (F). Amino acid misaminoacylation and the effect of increasing tRNA-copy-number on misaminoacylation was assessed by mass spectrometry using a UAG-containing MS–READ reporter co-expressed in host cells with [C1]-based tRNA copy number variants, (**[C1]–[C6]**). Amino acid incorporation events mediated by each OTS variant were quantified in Skyline using MS1 precursor ion intensities (area under the curve) averaged across three biological replicates, normalized to the level of pSer incorporation within the same sample, and displayed as a relative ratio for primary misincorporation events Gly:pSer (G, blue) and Thr:pSer (H, orange); error bars represent 1 SD. To identify the cause of misaminoacylation by host aaRSs, identity elements for *E. coli* tRNAs identified in (E, F) were overlaid onto the primary sequence of $tRNA^{pSer}$ to examine identity element overlap that may decrease fidelity and potential targets for mutational analysis aimed at decreasing host GlyRS (blue), ThrRS (orange) $tRNA^{pSer}$ recognition (I). The acceptor stem base pair C2:G71 was identified as a recognition element unique to host aaRSs and was selected for mutation. The effect of mutating C2:G7 to G2:C71 (generating OTS variant **[C1*]**) on aminoacylation fidelity was assessed by MS–READ analysis in host cells expressing **[C1*]** and analyzed by mass spectrometry as described in (E, F) and displayed as a relative ratio for primary misincorporation events Gly:pSer (blue) Ser:pSer (purple) and Thr:pSer (orange) from three biological replicates; error bars represent 1 SD OTS fidelity (corresponding to the same reporter protein analyzed by mass spectrometry) was qualitatively examined by Phos–Tag™ SDS–PAGE and immunoblot as described in (B) (indicated upper band = pSer and indicated lower band = non-pSer) (J). Amino acid misactivation (iii) was identified through a comparative analysis of Ser incorporation events present in previously examined samples (G, H). Overlap analysis of primary *E. coli* $tRNA^{Ser}$ identity elements with $tRNA^{pSer}$ shows almost no identity element overlap, with $tRNA^{pSer}$ missing the extended variable loop region required for recognition by host SerRS (K). Quantification of Ser incorporation was performed as described in (G, H) on three independent biological replicates; error bars representing 1 SD (L).

process is sensitive to the concentration of free, uncomplexed tRNA, especially when the overexpressed tRNA contains shared identity elements (Giege *et al*, 1998, 2012).

We identified host aaRSs that misrecognize $tRNA^{pSer}$ and cause mistranslation of UAG codons by overexpressing $tRNA^{pSer}$ (without pSerRS, **[C1 tRNA]**) in host cells alongside the MS–READ reporter and monitoring amino acid incorporation at UAG codons by mass spectrometry. We observed two primary amino acid incorporation events, Gly and Thr, not observed during our analysis of UAG misdecoding (Fig 4E). Furthermore, we quantified Gly and Thr incorporation events and found ~100-fold higher levels of incorporation relative to levels observed during misdecoding (Fig 4F).

These observations indicate that, in the absence of pSerRS, $tRNA^{pSer}$ is misrecognized by host glycyl-tRNA synthetase (GlyRS) and threonyl-tRNA synthetase (ThrRS), resulting in Gly and Thr misincorporation at UAG codons, respectively. To determine if the inclusion of pSerRS could prevent these off-target interactions, we challenged the host cell with OTS variants that increased the expression of $tRNA^{pSer}$ relative to the expression of pSerRS (**[C1]–[C6]**) and assessed the rate of Gly and Thr incorporation relative to pSer. Interestingly, despite competition from the cognate pSerRS, we still observed Gly and Thr incorporation at UAG codons. The levels of Gly and Thr incorporation, relative to pSer, were comparable and increased proportionally to increasing $tRNA^{pSer}$ expression, with Gly and Thr incorporation rates nearly equal to pSer during expression from **[C1]** and up to ~5-fold higher than pSer during expression from **[C6]** (Fig 4G and H). When matched to host fitness and OTS fidelity across the same OTS series, these data indicate that the host fitness and OTS fidelity reductions that we observe in Fig 4A and B may result from $tRNA^{pSer}$ misrecognition (and misaminoacylation) by the host Gly- and ThrRS. However, additional experiments involving a broader range of tRNA mutants will be required to concretely establish this causal relationship.

Because competition between aaRSs for tRNA substrates with ambiguous identity elements is the major source of tRNA misrecognition (Sherman *et al*, 1992), we reasoned that removing this ambiguity might also eliminate o-tRNA misrecognition. Based on prior work characterizing the recognition of tRNA by GlyRS and ThrRS, we identified tRNA identity elements for $tRNA^{Gly}$ and $tRNA^{Thr}$ and mapped them to the primary sequence of $tRNA^{pSer}$ to identify overlapping elements (Fig 4I; Hasegawa *et al*, 1992; Nameki *et al*, 1996, 1997). Both the host tRNAs have conserved primary identity elements in the acceptor stem of $tRNA^{pSer}$ (G1:C72 and C2:G71) and the discriminator base U73 (Park *et al*, 2011). Although both U73 and the G1:C72 pair in the acceptor stem of $tRNA^{pSer}$ serve as essential recognition elements for pSerRS, the C2:G71 base pair in the acceptor stem of $tRNA^{pSer}$ exclusively facilitates recognition by GlyRS and ThrRS and represents a prime target for mutational analysis aimed at reducing $tRNA^{pSer}$ misrecognition. To maintain the general sequence composition of $tRNA^{pSer}$, we chose to flip the acceptor stem pair and generated a G2:C71 variant of $tRNA^{pSer}$ ($tRNA^{pSer}_{Opt.}$, Fig 4I).

Employing the same experimental design used to test $tRNA^{pSer}$ misrecognition during expression from **[C1 tRNA]**, we overexpressed $tRNA^{pSer}_{Opt.}$ (without pSerRS, **[C1* tRNA]**) in host cells alongside the MS–READ reporter and monitoring amino acid

 © 2023 The Authors

incorporation at UAG codons by mass spectrometry. When compared with the expression of un-modified $tRNA^{pSer}$ from **[C1 tRNA]**, we observed a striking reduction in Gly incorporation (~100-fold), and Thr incorporation fell below the limit of detection (Fig 4F). Encouraged by these results, we constructed a complete OTS variant by exchanging the unmodified $tRNA^{pSer}$ from **[C1]** with one copy of the $tRNA^{pSer}_{Opt.}$ (**[C1*]**) and quantified amino acid incorporation at UAG codons using the MS–READ reporter. Relative to expression from **[C1]**, the incorporation rates of Gly:pSer and Thr:pSer decreased ~30-fold and ~1,000-fold, respectively, and reflect the reduction in non-pSer product observed by reporter protein immunoblot analysis (Fig 4J).

In addition to Gly and Thr, we observed conditional Ser incorporation at UAG codons dependent on the co-expression of pSerRS and $tRNA^{pSer}$. The absence of Ser incorporation during expression from **[C1 tRNA]** combined with insignificant overlap with host $tRNA^{Ser}$ primary identity elements (e.g., extended variable loop) substantially reduces the probability that Ser incorporation is due to misrecognition of $tRNA^{pSer}$ by the host seryl-tRNA synthetase (SerRS; Fig 4K; Lenhard *et al*, 1999). Excluding misrecognition by SerRS leaves two potential alternative sources of pSerRS-dependent Ser incorporation: de-phosphorylation of pSer to Ser after production of pSer–$tRNA^{pSer}$ or direct misactivation of Ser and transfer to $tRNA^{pSer}$ by pSerRS (Fig 4Ciii).

De-phosphorylation of pSer is unlikely to be the predominant source of Ser incorporation. If Ser incorporation is due to spontaneous hydrolysis of pSer, we expect a constant ratio of Ser:pSer incorporation independent of phosphoprotein yield. Alternatively, if de-phosphorylation is mediated enzymatically by host protein phosphatases, we would expect two distinct trends in Ser incorporation that depend on the phosphatase activity level relative to the phosphoprotein production rate. In the first scenario under phosphatase saturating conditions, where phosphatase activity exceeds the rate of phosphoprotein production, we would expect to see a fixed steady-state ratio of Ser:pSer dictated by the equilibrium between phosphoprotein production and the catalytic activity of the phosphatase. Under these conditions, the steady-state equilibrium should not be tRNA-dependent unless the rate of phosphoprotein production is enhanced by an increase in tRNA such that it exceeds the catalytic capacity of the phosphatase. In this alternative scenario, where phosphoprotein production exceeds phosphatase activity, we expect a decrease in the ratio of Ser:pSer proportional to phosphoprotein yield. However, our observations matched none of the scenarios above. Instead, we observed an increase in the relative ratio of Ser:pSer that, like Gly and Thr, increased proportional to increasing $tRNA^{pSer}$ expression up to ~8-fold during expression from **[C6]** relative to **[C1]** (Fig 4L). If we eliminate misrecognition and de-phosphorylation as the primary sources of Ser incorporation and consider previous work demonstrating low-level activation of Ser by pSerRS (~10% the rate of pSer activation; Hauenstein *et al*, 2008), our data are most consistent with Ser incorporation caused by pSerRS-mediated Ser misactivation. In this context, Ser misactivation would be enhanced if the concentration pSer was limited in the amino acid pool relative to the concentration of Ser. This scenario would affect the selectivity of pSerRS such that any increase in the amount of $tRNA^{pSer}$ available for aminoacylation, following pSer pool depletion, is likely to result in misacylation with lower-specificity near-cognate amino acid substrates like Ser and an increase in the relative ratio of Ser:pSer that is proportional to $tRNA^{pSer}$ expression.

OTS development guided by host biological tolerance enhances OTS performance

Addressing the sources of OTS-mediated toxicity identified above improves OTS performance. By comparing three OTS variants with host-optimized OTS component expression to their progenitor (**[λ]**), we demonstrate the ability of host-centric OTS design to identify baseline OTS-component expression with low-host toxicity (**[P1]**), modulate OTS copy number to improve OTS capacity (**[C1]**), and increase OTS fidelity through targeted modifications that enhance orthogonality (**[C1*]**; Fig 5A). To highlight these targeted improvements, we evaluated the effect of expression from **[P1]**, **[C1]**, or **[C1*]** variants on host cell growth, phosphoprotein yield and purity, and the molecular phenotype.

Tuning individual OTS-component expression improves host cell fitness. Consistent with previous results, we observed no significant fitness defects in cells expressing **[P1]**, **[C1]**, or **[C1*]**. In contrast, we observed a ~2-fold decrease in growth rate during expression from **[λ]** (*P*-value: 0.0001) relative to **[BG]** cells and a small but statistically significant increase in growth rate during expression from **[C1*]** (*P*-value: 0.01) relative to cells expressing **[C1]** (Fig 5B).

Optimizing OTS translational capacity by modulating OTS copy number improves phosphoprotein yield and purity. We assessed phosphoprotein yield and purity mediated by **[λ]** and low-toxicity **[P1]**, **[C1]**, or **[C1*]** variants using quantitative densitometry of MS–READ reporter immunoblots following separation of pSer-containing reporter by PhosTag SDS–PAGE. Consistent with previous observations, the yield of the MS–READ reporter mediated by **[P1]** and **[λ]** is low when compared with the yield from **[C1]** and **[C1*]** variants (Fig 5C; Lanes 2–3 vs. Lanes 4–5) and phosphoprotein purity is compromised during reporter expression from **[P1]** and **[C1]** variants due to Gly and Thr incorporation from misrecognition of $tRNA^{pSer}$, pre-optimization (Fig 5C; Lanes 3 and 4, non-pSer). Following quantitative analysis, we found that the phosphoprotein yield was similar between **[λ]** and **[P1]** variants at approximately 50 and 60 mg/l, respectively, though the fidelity of **[P1]** was marginally improved (~20%). However, the yield of phosphoprotein relative to **[λ]** increased 5–6-fold to ~330 mg/l using **[C1]** and ~380 mg/l using **[C1*]**. Phosphoprotein purity from **[C1]** was similar to **[λ]** but improved by ~50% during expression using **[C1*]** (Fig 5D).

Including host-optimized $tRNA^{pSer}$ with enhanced orthogonality reduces proteome dysregulation caused by cellular stress. We performed comparative molecular phenotyping of host proteomes during expression from **[C1*]** relative to expression from **[C1]** and found 105 significantly dysregulated proteins, of which nearly 80% were downregulated (Fig 5E). In addition, we analyzed proteins with lower abundance in **[C1*]** for GO term enrichment and constructed a functionally enriched network for amino acid biosynthetic processes (network *P*-value = $1.8e^{-6}$) and, more specifically, biological process enrichment for aspartate-family amino acid biosynthesis (Fig 5F; FDR: $5.8e^{-6}$). These results are consistent with a reduction in the host cellular stress response that regulates amino acid metabolism (i.e., the stringent response) in cells expressing the ultimate host-optimized **[C1*]** variant.

© 2023 The Authors

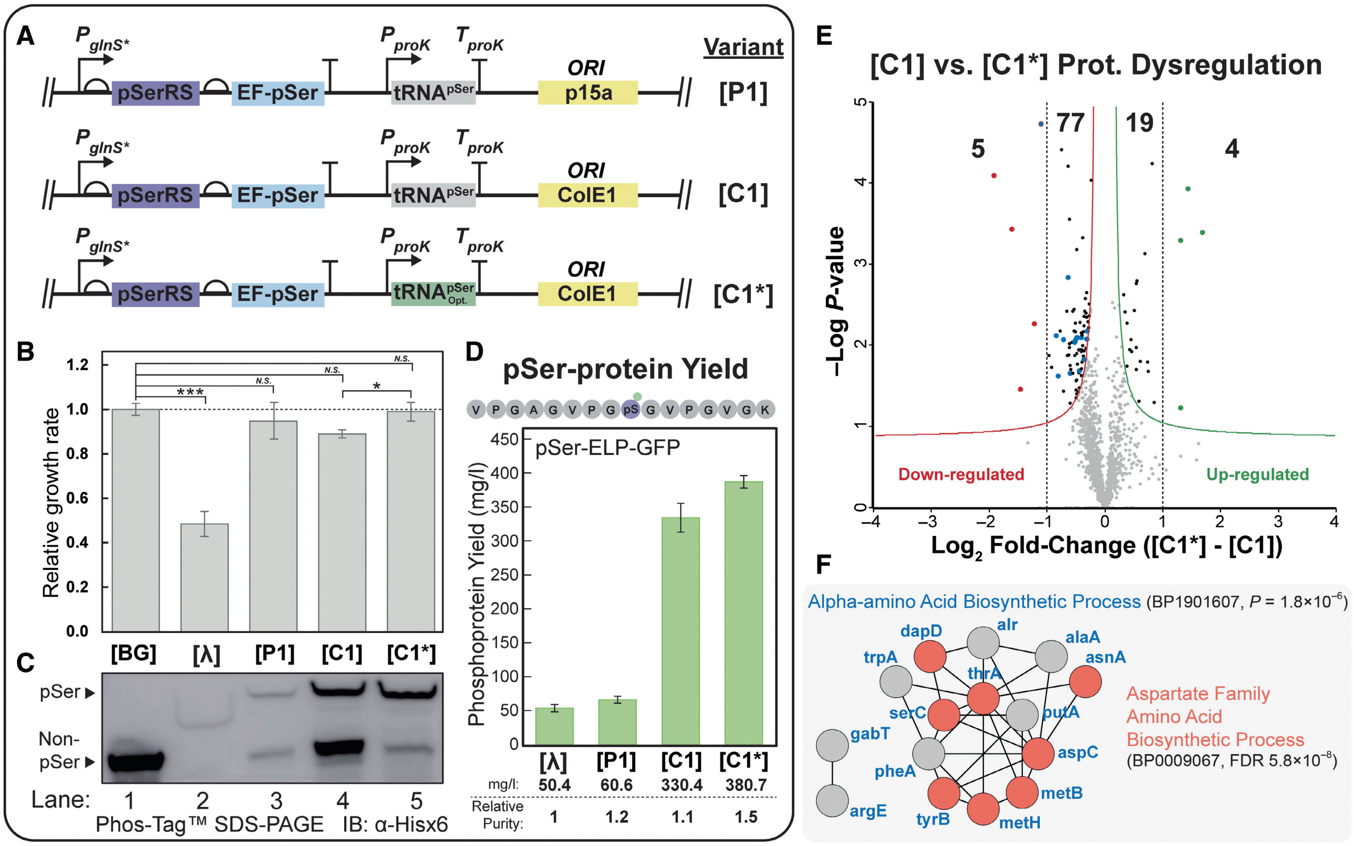

Figure 5. Assessing the performance of host-optimized OTS variants.

A–F OTS architecture for optimized OTS variants **[P1]**, **[C1]**, and **[C1*]** (A). The effect of OTS variant expression on host growth rate was assessed by monitoring cell density (absorbance at 600 nm) in host cells **[BG]** and cells expressing either **[λ]** or OTS variants from (A) over time. All the growth rate parameters reflect the average measurements from three independent biological replicates with error bars representing 1 SD. Statistical significance of growth rate changes was assessed by two-tailed student's *t*-test (equal variance) where * equals $P < 0.05$, ** equals $P < 0.005$, *** equals $P < 0.0005$, and *N.S.* denotes Not Significant (B). OTS performance was preliminarily compared with OTS variant growth through qualitative assessment of pSer incorporation fidelity determined by separation of a UAG-containing MS-READ reporter on a Phos–tag™ SDS–PAGE and immunoblot analysis. Each lane was loaded with 7.5 μl of O.D.-normalized cell lysate (C). For quantitative assessment of OTS performance, absolute phosphoprotein yield and purity (relative to **[λ]**) facilitated by host cells expressing **[λ]**, **[P1]**, **[C1]**, or **[C1*]** was assessed by densitometric analysis of Phos–tag™ SDS–PAGE resolved pSer-containing MS–READ reporter protein. Each lane was loaded with 0.5 O.D. equivalents of O.D.-normalized cell lysate. Reported values represent the average phosphoprotein yield from three biological replicates with error bars representing 1 SD (D). Alteration of the proteomic composition mediated by o-tRNA optimization was quantified by mass spectrometry. Proteomic dysregulation and statistical analysis were conducted in Perseus and illustrated as a volcano plot displaying differences in individual protein expression from host cells expressing **[C1*]** relative to **[C1]**. Statistically significant dysregulated proteins (black circles) were identified by pair-wise *t*-test ($P < 0.05$) and FDR (0.1) calculation from three independent biological replicates and fall outside the asymptotic lines (green demarking upregulation and red downregulation) with the expression of individual proteins involved in alpha-amino acid biosynthesis highlighted (blue circles) (E). Significantly down-regulated proteins in **[C1*]** were analyzed in stringDB to identify statistically significant pathway and biological process enrichments, yielding a functionally enriched protein network for alpha-amino acid biosynthetic processes (blue labels corresponding to blue circles in (E)) and sub-network enrichment for aspartate family amino acid biosynthetic processes (red nodes) (F).

Discussion

Defining the host cell's role in OTS performance

OTSs enable the incorporation of a vast collection of non-standard amino acids, facilitating the characterization of protein:substrate interactions, dissection of enzyme function, and the creation of synthetically modified proteins with industrial applications. However, despite their widespread use, our understanding of the interactions between OTSs and host cells and the resulting impact on target protein expression remains limited. This study provides the first systematic characterization of the global effects of OTS introduction to host *E. coli* cells, using pSerOTS as a model system. Our findings reveal that the host cell's role in OTS-mediated protein expression extends beyond that of a simple chassis and that complex OTS:host interactions influence transcriptional and translational programming through stress responses and resource re-allocation, which affects OTS performance.

Typically, OTSs for recombinant protein expression are deployed in traditional protein expression strains, such as BL21, modified to reduce proteolytic degradation and enhance overall protein yield. However, this feature dramatically reduces the mechanisms available to the cell to counteract the toxic effects of off-target stop codon suppression mediated by o-tRNA and premature translation

 © 2023 The Authors

termination of UAG-containing heterologously expressed protein, significantly reducing yield in the presence of an OTS. The development of genetically recoded organisms has provided an effective means to bypass the detrimental effects of off-target stop codon suppression, but the potential for deleterious interactions with the host cell remains through a breakdown in OTS orthogonality (Lajoie *et al*, 2013; Pirman *et al*, 2015).

OTS orthogonality is often regarded as a static condition. However, much like the host cell itself, this simplification is complicated by the dynamic nature of the cell's response to external stimuli and internally by the basal demands of physiological processes. Our work here highlights these complexities and the susceptibility of OTS performance to host-mediated reductions in OTS orthogonality, often overlooked during OTS development and deployment. For example, maintaining the balance of tRNAs in the tRNA pool is crucial to maintaining translational fidelity and regulation. Altering tRNA pool composition is known to increase the synthesis of mispaired aa-tRNA, resulting in protein mistranslation and reduced regulatory capacity of translation processes (Swanson *et al*, 1988; O'Connor, 1998). Although most mispaired aa-tRNAs are corrected through carefully evolved aa-tRNA proofreading mechanisms, o-tRNAs are rarely developed in the context of these editing mechanisms and may evade them (Jakubowski & Fersht, 1981; Roy *et al*, 2004; Martinis & Boniecki, 2010).

While our understanding of the consequences of natural perturbations to the tRNA pool has expanded, little has been done outside the present work to assess the impact of introducing heavily evolved orthogonal tRNAs, specifically on host physiology. In this work, we demonstrate through a proteome-centric approach that introducing heavily evolved o-tRNAs reduces host cell fitness by compromising translational fidelity in a concentration-dependent manner through interactions with native aminoacyl–tRNA synthetases. Traditional approaches to assess tRNA orthogonality fail to account for this aspect of tRNA substrate recognition and therefore overlook reductions in OTS fidelity caused by alteration of tRNA selectivity rather than tRNA specificity during o-tRNA optimization. Our comprehensive analysis strategy centers on an unbiased survey of mispaired o-tRNA via amino acid misincorporation and molecular phenotyping. The results of this analysis offer unparalleled insight into OTS:host cell interactions and can be utilized to further our understanding of tRNA, host cell, and OTS biology.

Our findings illustrate the vast complexity of potential OTS:host interactions and the need for detailed analysis of OTS performance and biological tolerance within the context of the host cell. Further characterization of these interactions will enable more informed OTS design practices, limiting the fitness defects OTSs impart on host cells while improving OTS performance and robustness across various experimental settings.

Establishing permanent OTS installation through systematic characterization

Using plasmid vectors to express OTS components poses challenges to their performance and reliability. Plasmid copy number and stability can vary widely depending on the origin of plasmid replication, host strain genotype, and growth conditions, making it challenging to predict OTS behavior under experimental conditions that deviate from those used during OTS optimization (Wegrzyn, 1999). An alternative approach to increase reliability and ease of use would be permanently integrating the OTS into the host cell genome. While OTSs have been genomically integrated previously, little consideration has been given to how this affects the host environment and interactions with host translational machinery (Amiram *et al*, 2015). In this study, we propose a systematic approach to benchmark variations in OTS component expression against native host cellular processes while simultaneously monitoring OTS efficiency. Establishing the optimal expression levels of o-aaRS and o-tRNA in the proteome and cellular substrate pools relative to their host cell counterparts will be crucial to ensure the stable integration of OTSs into the genome. Overexpression of an OTS component can cause stress that may result in a negative selection pressure against maintaining a genomically encoded OTS. Over the years, we and others have observed frequent transposon inactivation of the highly expressed pSerRS from pSerOTSλ, reinforcing the importance of data-driven design practices to guide the future integration and expression of OTS components at levels comparable to those of native substrate pools in addition to improving component orthogonality. Successful installation of OTSs into the translational complement of host cells will improve the ease of use and overall reliability of these tools, making stable genomic integration a promising target for the future of OTS biology.

Materials and Methods

Cell growth and general techniques

All the DNA oligonucleotides with standard purification and desalting and Sanger DNA sequencing services were obtained through the Keck DNA Sequencing facility and Oligonucleotide Resource, Yale University. Unless otherwise stated, all cultures were grown at 37°C in LB–Lennox medium (LB, 10 g/l bacto tryptone, 5 g/l sodium chloride, 5 g/l yeast extract). LB agar plates were LB plus 16 g/l bacto agar. Antibiotics were supplemented for selection, where appropriate (Kanamycin, 50 μg/ml and Ampicillin, 100 μg/ml). *E. coli* Top10 cells (Invitrogen, Carlsbad, CA) were used for cloning and plasmid propagation. NEBuilder HiFi Assembly Mix and restriction enzymes were obtained from New England BioLabs. Plasmid DNA was prepared with the QIAprep Spin Miniprep Kit (Qiagen). Pre-cast 4–12% (wt/vol) Bis–Tris gels for SDS–PAGE were purchased from Bio–Rad. Phos–Tag™ reagent for hand-cast protein gels was purchased from Wako.

A complete list of strains and plasmids is provided in Appendix Table S3. All the plasmids were transformed into recipient strains by electroporation. Electrocompetent cells were prepared by inoculating 20 ml of LB with 200 μl of saturated culture and growing at 37°C until reaching an OD_{600} of 0.4. Cells were harvested by centrifugation at 6,000 × *g* for 2 min. at 4°C. Cells pellets were washed twice with 20 ml ice-cold 10% glycerol in deionized water (dH_2O). Electrocompetent pellets were re-suspended in 100 μl of 10% glycerol in dH_2O. 50 ng of plasmid was mixed with 50 μl of re-suspended electrocompetent cells and transferred to 0.1 cm cuvettes, electroporated (BioRad GenePulser™, 1.78 kV, 200 Ω, 25 μF), and then immediately resuspended in 600 μl of LB. Transformed cells were recovered at 37°C for 1 h, and 100 μl was subsequently plated on an appropriate selective medium.

© 2023 The Authors

Construction of OTS vectors

The list of plasmids used in this study is found in Appendix Table S3. Generally, OTS components were amplified from the progenitor OTS, pSerOTSλ (SepOTSλ, Addgene # 68292). The pSerRS (SepRS9) and EF-pSer (EF-Sep21) were amplified from pSerOTSλ and placed under the control of the glnS* promoter and assembled with the kanamycin selection marker and origin of replication from pSerOTSλ by gibson assembly using NEBuilder HiFi DNA Assembly master mix (NEB). tRNA expression cassettes (1×-, 2×-, 4×-, and 6×-tRNA) were constructed from a 2×-tRNA construct under the control of a proK tRNA promoter and terminator and separated by a valU tRNA linker that was ordered as a DNA fragment from Genewiz. tRNA cassettes were assembled into the previously assembled pSerRS-EF-pSer backbone by gibson assembly, creating ColE1-based pSerOTSc OTSs. OTS variants with different origins of replication were created by the assembly of Rop amplified from pSerOTSλ into the ColE1-based OTSs, and p15a variants were created by replacement of ColE1 with p15a origins by gibson assembly. The fully optimized pSerOTS **[C1*]** is available through Addgene (pSerOTS-C1*, #188537).

Construction of $rEcoli^{XpS}$

Strain modification using a lambda red-based strategy was performed as previously described (Datsenko & Wanner, 2000). Briefly, transformants carrying a lambda red plasmid (pKD46) were grown in 20 ml LB cultures with ampicillin and l-arabinose at 30°C to an OD_{600} of ≈0.6 and then made electrocompetent. 10–100 ng of PCR product targeting serB was transformed into the prepared cells and recovered in 1 ml of LB for 1 h at 37°C. Following recovery, one-half was spread onto LB-agar plates with Kanamycin for selection. serB gene deletion was verified by PCR, and selected colonies were purified non-selectively at 37°C to remove pKD46. The KAN deletion cassette was excised from the serB locus using FLP recombinase (pCP20). $rEcoli^{XpS}$ is available through Addgene (#192872).

Multi-parametric cellular fitness analysis

Parameters used to calculate relative fitness metrics were collected and analyzed as described previously (Akerlund *et al*, 1995; Fernandez-Ricaud *et al*, 2016) and synthesized to produce discrete relative fitness values as follows:

Characterization of cell growth parameters

Stationary phase pre-cultures were obtained by overnight growth with shaking at 37°C in 5 ml of LB supplemented with antibiotic for plasmid maintenance, where appropriate. Stationary phase cultures were diluted to an OD_{600} of 0.01 in 250 µl of LB supplemented with appropriate antibiotic. Growth was monitored by measuring the optical density (absorbance at 600 nm, OD_{600}) on a Biotek Synergy H1 microplate reader at 10-min intervals for 16 h at 37°C with continuous double orbital shaking. All the data were measured in triplicate. Cellular growth parameters for lag phase endpoint ($_{\lambda}$), specific growth rate (μ), and growth efficiency (maximum cell density, OD_{Max}) were determined by analysis in PRECOG (Fernandez-Ricaud *et al*, 2016). Briefly, the lag phase endpoint was determined by identifying the intercept of log-transformed initial cell population density with the line extrapolated from the maximum specific growth rate. The growth rate was determined by calculating the slopes of log-transformed data (excluding the first 3 h) in a moving window of three time-points and then creating a rank-ordered list, highest to lowest. The five highest discrete slopes (excluding the two highest slopes) are then averaged to determine the specific growth rate. Growth efficiency was calculated as the total increase in population size based on the difference between the average of the two lowest cell density readings and the six highest cell density readings (i.e., $OD_{Max} - OD_{Min}$). Cellular growth parameters, as described above, represent the average values extracted from three biological replicates for each phenotypic characterization.

Characterization of cell size parameters

Cell cultures (5 ml) were grown overnight and diluted to an OD_{600} of 0.10 in 5 ml of LB containing antibiotic, where appropriate, and grown until the cell population reached mid-log (OD_{600} ∼0.4–0.6). 50 µl of cells were mixed with 3 ml ice-cold PBS in a 5 ml polystyrene tube (BD Falcon) for flow cytometry analysis using a BD FACSAria III. For each sample, one million cells were measured by flow cytometry, with forward light scattering (FSC) used as a proxy for cell size, as described previously (Akerlund *et al*, 1995). Cell size distribution was visualized as a histogram representing individual FSC values by abundance within the population and analyzed to determine the mean, mean SD, median, median SD, mode, and mode SD of cell size using FlowJo version 10.8.1 (Flowstar).

Analytical gel and immunoblotting

For qualitative analysis of protein yield, 7.5 µl of normalized cell lysate (prepared from lysis of 8 OD_{600} cell equivalents in 120 µl of lysis buffer) were analyzed by Phos–tag SDS–PAGE and SDS–PAGE. 100 µM Phos–tag™ acrylamide (Wako) within hand-cast 12% acrylamide gels were used for the separation of phosphorylated reporter proteins. SDS–PAGE gels (4–15% acrylamide, Bio-Rad) and Phos–tag™ gels were transferred onto PVDF membranes. All the gels were visualized by immunoblot. Anti–His immunoblots were performed using 1:2,500 diluted rabbit Anti–6×His antibody (PA1-983B, Thermo Fisher Scientific) in 5% w/v milk in TBST for 1 h. Secondary antibody incubations used 1:10,000 diluted donkey anti-rabbit HRP (711-035-152, Jackson ImmunoResearch) in 5% w/v milk in TBST for 1 h. Protein bands were then visualized using Clarity ECL substrate (Bio-Rad) and an Amersham Imager 600 (GE Healthcare Life Sciences). Densitometry analysis was performed using ImageJ (Schneider *et al*, 2012). For quantitative analysis of protein yield, cell lysates corresponding to 0.5 OD_{600} equivalents were run on both Phos–tag SDS–PAGE and SDS–PAGE alongside a series of 6×His tagged GFP standards of known concentration and processed as described above. For total protein yield, values were extrapolated based on GFP standard curve intensities. For phosphoprotein yield, the total protein yield was adjusted based on the ratio of phosphorylated to the non-phosphorylated product derived from densitometry analysis of immunoblots of the same samples separated by Phos–tag SDS–PAGE.

MS–READ reporter purification

Frozen *E. coli* cell pellets were thawed on ice, and pellets were lysed by sonication with lysis buffer consisting of 50 mM Tris–HCl (pH

© 2023 The Authors

7.4, 23°C), 500 mM NaCl, 0.5 mM EGTA, 1 mM DTT, 10% glycerol, 50 mM NaF, and 1 mM Na_3O_4V. The extract was clarified with two rounds of centrifugation performed for 20 min at 4°C and 14,000 × *g*. Cell-free extracts were applied to Ni–NTA metal affinity resin and purified according to the manufacturer's instructions. Wash buffers contained 50 mM Tris pH 7.5, 500 mM NaCl, 0.5 mM EGTA, 1 mM DTT, 50 mM NaF, 1 mM Na_3VO_4, and increasing concentrations of imidazole 20 mM, 40 mM, and 60 mM, sequentially. Proteins were eluted with a wash buffer containing 250 mM imidazole. Eluted protein was subjected to four rounds of buffer exchange (20 mM Tris pH 8.0 and 100 mM NaCl) and concentrated using a 30 kDa molecular weight cutoff spin filter (Amicon).

Protein digestion and mass spectrometry

MS–READ analysis

Affinity purified, buffer exchanged protein was digested and analyzed by mass spectrometry as described previously with some modifications. Briefly, the concentration of protein was determined by UV280 spectroscopy, and 5 μg ELP–GFP (MS–READ) reporter from *E. coli* was dissolved in 12.5 μl solubilization buffer consisting of 10 mM Tris–HCl pH = 8.5 (23°C), 10 mM DTT, 1 mM EDTA and 0.5% acid labile surfactant (ALS-101, Protea). Samples were heat denatured for 20 min at 55°C in a heat block. Alkylation of cysteines was performed with iodoacetamide (IAM) using a final IAM concentration of 24 mM. The alkylation reaction proceeded for 30 min at room temperature in the dark. Excess IAM was quenched with DTT, and the buffer concentration was adjusted using a 1 M Tris–HCl pH 8.5, resulting in a final Tris–HCl concentration of 150 mM. The reaction was then diluted with water and 1 M $CaCl_2$ solution to obtain an ALS-101 concentration of 0.045% and 2 mM $CaCl_2$, respectively. Finally, sequencing grade porcine trypsin (Promega) was added to obtain an enzyme/protein ratio of 1/5.3, and the digest was incubated for 15 h at 37°C without shaking. The digest was quenched with 20% TFA solution resulting in a sample pH of 2. Cleavage of the acid-cleavable detergent proceeded for 15 min at room temperature. Digests were frozen at −80°C until further processing. Peptides were desalted on C_{18} UltraMicroSpin columns (The Nest Group Inc.), essentially following the instructions provided by the manufacturer but using 300 μl elution solvent consisting of 80% ACN, 0.1% TFA for peptide elution. Peptides were dried in a vacuum centrifuge at room temperature. Dried peptides were reconstituted and analyzed by LC–MS/MS.

Digestion of intact E. coli for shotgun proteomics

20 ml cultures were inoculated to a starting OD_{600} of 0.01 in LB media using an overnight culture to stationary phase. After reaching mid-log, cells were chilled on ice and pelleted by centrifugation for 2 min at 6,000 × *g*. The resulting pellet was frozen at −80°C for downstream processing. For cell lysis and protein digest, cell pellets were thawed on ice, and 2 μl of cell pellet was transferred to a microcentrifuge tube containing 40 μl of lysis buffer (10 mM Tris–HCl pH 8.6, 10 mM DTT, 1 mM EDTA, and 0.5% ALS). Cells were lysed by vortex for 30 s, and disulfide bonds were reduced by incubating the reaction for 30 min at 55°C. The reaction was briefly quenched on ice, and 16 μl of a 60 mM IAM solution was added. Alkylation of cysteines proceeded for 30 min in the dark. Excess IAM was quenched with 14 μl of a 25 mM DTT solution, and the sample was then diluted with 330 μl of 183 mM Tris–HCl buffer pH 8.0 supplemented with 2 mM CaCl2. Proteins were digested overnight using 12 μg sequencing grade trypsin. Following digestion, the reaction was quenched with 12.5 μl of a 20% TFA solution, resulting in a sample pH < 3. The remaining ALS reagent was cleaved for 15 min at room temperature. The sample (~30 μg protein) was desalted by reverse phase clean-up using C18 UltraMicroSpin columns. The desalted peptides were dried at room temperature in a rotary vacuum centrifuge and reconstituted in 30 μl 70% formic acid 0.1% TFA (3:8 v/v) for peptide quantitation by UV280. The sample was diluted to a final concentration of 0.2 μg/μl, and 5 μl (1 μg) was injected for LC–MS/MS analysis.

Data acquisition and analysis

LC–MS/MS was performed using an ACQUITY UPLC M-Class (Waters) and Q Exactive Plus mass spectrometer. The analytical column employed was a 65-cm-long, 75-μm-internal-diameter PicoFrit column (New Objective) packed in-house to a length of 50 cm with 1.9 μm ReproSil-Pur 120 Å C18-AQ (Dr. Maisch) using methanol as the packing solvent. Peptide separation was achieved using mixtures of 0.1% formic acid in water (solvent A) and 0.1% formic acid in acetonitrile (solvent B) with either a 90-min gradient 0/1, 2/7, 60/24, 65/48, 70/80, 75/80, 80/1, 90/1; (min/%B, linear ramping between steps). The gradient was performed with a flowrate of 250 nl/min. At least one blank injection (5 μl 2% B) was performed between samples to eliminate peptide carryover on the analytical column. 100 fmol of trypsin-digested BSA or 100 ng trypsin-digested wildtype K-12 MG1655 *E. coli* proteins were run periodically between samples as quality control standards. The mass spectrometer was operated with the following parameters: (MS1) 70,000 resolution, $3e^6$ AGC target, 300–1,700 m/z scan range; (data dependent-MS2) 17,500 resolution, $1e^6$ AGC target, top 10 mode, 1.6 m/z isolation window, 27 normalized collision energy, 90 s dynamic exclusion, unassigned and +1 charge exclusion. Data were searched using MaxQuant version 1.6.10.43 with Deamidation (NQ), Oxidation (M), and Phospho(STY) as variable modifications and Carbamidomethyl (C) as a fixed modification with up to three missed cleavages, five AA minimum length, and 1% FDR against a modified Uniprot *E. coli* database containing custom MS–READ reporter proteins. MS–READ search results were analyzed using Skyline version 20.1.0.31, and proteome search results were analyzed with Perseus version 1.6.2.2.

Data availability

The mass spectrometry proteomics data have been deposited to the ProteomeXchange Consortium via the PRIDE (Perez-Riverol *et al*, 2019). Datasets can be found under the following accession number: PXD026217 (http://www.ebi.ac.uk/pride/archive/projects/PXD026217).

Expanded View for this article is available online.

Acknowledgements

Funding was provided by the Yale School of Medicine, Yale West Campus, Systems Biology Institute. The National Institutes of Health grants F32CA224946 (KM) and GM117230 (JR).

© 2023 The Authors

Author contributions

Kyle Mohler: Conceptualization; data curation; formal analysis; validation; investigation; visualization; methodology; writing – original draft; writing – review and editing. **Jack M Moen:** Conceptualization; data curation; investigation; writing – review and editing. **Svetlana Rogulina:** Resources; data curation; investigation. **Jesse Rinehart:** Conceptualization; resources; supervision; funding acquisition; project administration; writing – review and editing.

Disclosure and competing interests statement

JR is on the scientific advisory board of Pearl Bio. The other authors declare that they have no conflict of interest.

References

Aerni HR, Shifman MA, Rogulina S, O'Donoghue P, Rinehart J (2015) Revealing the amino acid composition of proteins within an expanded genetic code. *Nucleic Acids Res* 43: e8

Ahel I, Korencic D, Ibba M, Söll D (2003) Trans-editing of mischarged tRNAs. *Proc Natl Acad Sci USA* 100: 15422–15427

Akerlund T, Nordstrom K, Bernander R (1995) Analysis of cell size and DNA content in exponentially growing and stationary-phase batch cultures of *Escherichia coli*. *J Bacteriol* 177: 6791–6797

Amiram M, Haimovich AD, Fan C, Wang YS, Aerni HR, Ntai I, Moonan DW, Ma NJ, Rovner AJ, Hong SH *et al* (2015) Evolution of translation machinery in recoded bacteria enables multi-site incorporation of nonstandard amino acids. *Nat Biotechnol* 33: 1272–1279

Ataullakhanov FI, Vitvitsky VM (2002) What determines the intracellular ATP concentration. *Biosci Rep* 22: 501–511

Atkinson DE (1977) 4 – adenylate control and the adenylate energy charge. In *Cellular Energy Metabolism and Its Regulation*, Atkinson DE (ed), pp 85–107. San Diego, CA: Academic Press

Bedouelle H, Guez V, Vidal-Cros A, Hermann M (1990) Overproduction of tyrosyl-tRNA synthetase is toxic to *Escherichia coli*: a genetic analysis. *J Bacteriol* 172: 3940–3945

Belrhali H, Yaremchuk A, Tukalo M, Berthet-Colominas C, Rasmussen B, Bosecke P, Diat O, Cusack S (1995) The structural basis for seryl-adenylate and Ap4A synthesis by seryl-tRNA synthetase. *Structure* 3: 341–352

Bertrand RL (2019) Lag phase is a dynamic, organized, adaptive, and evolvable period that prepares bacteria for cell division. *J Bacteriol* 201: e00697-18

Biselli E, Schink SJ, Gerland U (2020) Slower growth of *Escherichia coli* leads to longer survival in carbon starvation due to a decrease in the maintenance rate. *Mol Syst Biol* 16: e9478

Biswas DK, Gorini L (1972) Restriction, de-restriction and mistranslation in missense suppression. Ribosomal discrimination of transfer RNA's. *J Mol Biol* 64: 119–134

Bullwinkle TJ, Ibba M (2016) Translation quality control is critical for bacterial responses to amino acid stress. *Proc Natl Acad Sci USA* 113: 2252–2257

Caglar MU, Houser JR, Barnhart CS, Boutz DR, Carroll SM, Dasgupta A, Lenoir WF, Smith BL, Sridhara V, Sydykova DK *et al* (2017) The *E. coli* molecular phenotype under different growth conditions. *Sci Rep* 7: 45303

Campos M, Govers SK, Irnov I, Dobihal GS, Cornet F, Jacobs-Wagner C (2018) Genomewide phenotypic analysis of growth, cell morphogenesis, and cell cycle events in *Escherichia coli*. *Mol Syst Biol* 14: e7573

Camps M (2010) Modulation of ColE1-like plasmid replication for recombinant gene expression. *Recent Pat DNA Gene Seq* 4: 58–73

Chalker AF, Ward JM, Fosberry AP, Hodgson JE (1994) Analysis and toxic overexpression in *Escherichia coli* of a staphylococcal gene encoding isoleucyl-tRNA synthetase. *Gene* 141: 103–108

Chatterjee A, Sun SB, Furman JL, Xiao H, Schultz PG (2013) A versatile platform for single- and multiple-unnatural amino acid mutagenesis in *Escherichia coli*. *Biochemistry* 52: 1828–1837

Chen M, Kato K, Kubo Y, Tanaka Y, Liu Y, Long F, Whitman WB, Lill P, Gatsogiannis C, Raunser S *et al* (2017) Structural basis for tRNA-dependent cysteine biosynthesis. *Nat Commun* 8: 1521

Datsenko KA, Wanner BL (2000) One-step inactivation of chromosomal genes in *Escherichia coli* K-12 using PCR products. *Proc Natl Acad Sci USA* 97: 6640–6645

Diaz Ricci JC, Hernandez ME (2000) Plasmid effects on *Escherichia coli* metabolism. *Crit Rev Biotechnol* 20: 79–108

Durrschmid K, Reischer H, Schmidt-Heck W, Hrebicek T, Guthke R, Rizzi A, Bayer K (2008) Monitoring of transcriptome and proteome profiles to investigate the cellular response of *E. coli* towards recombinant protein expression under defined chemostat conditions. *J Biotechnol* 135: 34–44

Dworkin J (2023) Understanding the stringent response: experimental context matters. *MBio* 14: e0340422

Englert M, Moses S, Hohn M, Ling J, O'Donoghue P, Soll D (2013) Aminoacylation of tRNA 2′- or 3′-hydroxyl by phosphoseryl- and pyrrolysyl-tRNA synthetases. *FEBS Lett* 587: 3360–3364

Farr SB, Arnosti DN, Chamberlin MJ, Ames BN (1989) An apaH mutation causes AppppA to accumulate and affects motility and catabolite repression in *Escherichia coli*. *Proc Natl Acad Sci USA* 86: 5010–5014

Fernandez-Ricaud L, Kourtchenko O, Zackrisson M, Warringer J, Blomberg A (2016) PRECOG: a tool for automated extraction and visualization of fitness components in microbial growth phenomics. *BMC Bioinformatics* 17: 249

Gan Q, Fan C (2017) Increasing the fidelity of noncanonical amino acid incorporation in cell-free protein synthesis. *Biochim Biophys Acta Gen Subj* 1861: 3047–3052

Giege R, Sissler M, Florentz C (1998) Universal rules and idiosyncratic features in tRNA identity. *Nucleic Acids Res* 26: 5017–5035

Giege R, Juhling F, Putz J, Stadler P, Sauter C, Florentz C (2012) Structure of transfer RNAs: similarity and variability. *Wiley Interdiscip Rev RNA* 3: 37–61

Gill RT, Valdes JJ, Bentley WE (2000) A comparative study of global stress gene regulation in response to overexpression of recombinant proteins in *Escherichia coli*. *Metab Eng* 2: 178–189

Gouveia D, Grenga L, Pible O, Armengaud J (2020) Quick microbial molecular phenotyping by differential shotgun proteomics. *Environ Microbiol* 22: 2996–3004

Gruic-Sovulj I, Uter N, Bullock T, Perona JJ (2005) tRNA-dependent aminoacyl-adenylate hydrolysis by a nonediting class I aminoacyl-tRNA synthetase. *J Biol Chem* 280: 23978–23986

Gruic-Sovulj I, Rokov-Plavec J, Weygand-Durasevic I (2007) Hydrolysis of non-cognate aminoacyl-adenylates by a class II aminoacyl-tRNA synthetase lacking an editing domain. *FEBS Lett* 581: 5110–5114

Hamill PG, Stevenson A, McMullan PE, Williams JP, Lewis ADR, Sudharsan S, Stevenson KE, Farnsworth KD, Khroustalyova G, Takemoto JY *et al* (2020) Microbial lag phase can be indicative of, or independent from, cellular stress. *Sci Rep* 10: 5948

Han NC, Bullwinkle TJ, Loeb KF, Faull KF, Mohler K, Rinehart J, Ibba M (2020) The mechanism of beta-N-methylamino-l-alanine inhibition of tRNA aminoacylation and its impact on misincorporation. *J Biol Chem* 295: 1402–1410

 © 2023 The Authors

Hasegawa T, Miyano M, Himeno H, Sano Y, Kimura K, Shimizu M (1992) Identity determinants of *E. coli* threonine tRNA. *Biochem Biophys Res Commun* 184: 478–484

Hauenstein SI, Hou YM, Perona JJ (2008) The homotetrameric phosphoseryl-tRNA synthetase from *Methanosarcina mazei* exhibits half-of-the-sites activity. *J Biol Chem* 283: 21997–22006

Heinemann IU, Rovner AJ, Aerni HR, Rogulina S, Cheng L, Olds W, Fischer JT, Soll D, Isaacs FJ, Rinehart J (2012) Enhanced phosphoserine insertion during *Escherichia coli* protein synthesis via partial UAG codon reassignment and release factor 1 deletion. *FEBS Lett* 586: 3716–3722

Humphrey B, Thomson NR, Thomas CM, Brooks K, Sanders M, Delsol AA, Roe JM, Bennett PM, Enne VI (2012) Fitness of *Escherichia coli* strains carrying expressed and partially silent IncN and IncP1 plasmids. *BMC Microbiol* 12: 53

Ibba M, Soll D (2000) Aminoacyl-tRNA synthesis. *Annu Rev Biochem* 69: 617–650

Isaacs FJ, Carr PA, Wang HH, Lajoie MJ, Sterling B, Kraal L, Tolonen AC, Gianoulis TA, Goodman DB, Reppas NB *et al* (2011) Precise manipulation of chromosomes in vivo enables genome-wide codon replacement. *Science* 333: 348–353

Jakubowski H (2012) Quality control in tRNA charging. *Wiley Interdiscip Rev RNA* 3: 295–310

Jakubowski H, Fersht AR (1981) Alternative pathways for editing non-cognate amino acids by aminoacyl-tRNA synthetases. *Nucleic Acids Res* 9: 3105–3117

Jakubowski H, Goldman E (1992) Editing of errors in selection of amino acids for protein synthesis. *Microbiol Rev* 56: 412–429

Ji X, Zou J, Peng H, Stolle AS, Xie R, Zhang H, Peng B, Mekalanos JJ, Zheng J (2019) Alarmone Ap4A is elevated by aminoglycoside antibiotics and enhances their bactericidal activity. *Proc Natl Acad Sci USA* 116: 9578–9585

Jin X, Park OJ, Hong SH (2019) Incorporation of non-standard amino acids into proteins: challenges, recent achievements, and emerging applications. *Appl Microbiol Biotechnol* 103: 2947–2958

Johnson DB, Xu J, Shen Z, Takimoto JK, Schultz MD, Schmitz RJ, Xiang Z, Ecker JR, Briggs SP, Wang L (2011) RF1 knockout allows ribosomal incorporation of unnatural amino acids at multiple sites. *Nat Chem Biol* 7: 779–786

Johnson DB, Wang C, Xu J, Schultz MD, Schmitz RJ, Ecker JR, Wang L (2012) Release factor one is nonessential in *Escherichia coli*. *ACS Chem Biol* 7: 1337–1344

Kamtekar S, Hohn MJ, Park HS, Schnitzbauer M, Sauerwald A, Söll D, Steitz TA (2007) Toward understanding phosphoseryl-$tRNA^{Cys}$ formation: the crystal structure of *Methanococcus maripaludis* phosphoseryl-tRNA synthetase. *Proc Natl Acad Sci USA* 104: 2620–2625

Kelly P, Backes N, Mohler K, Buser C, Kavoor A, Rinehart J, Phillips G, Ibba M (2019) Alanyl-tRNA synthetase quality control prevents global dysregulation of the *Escherichia coli* proteome. *mBio* 10: e02921-19

Krishnamurthi VR, Niyonshuti II, Chen J, Wang Y (2021) A new analysis method for evaluating bacterial growth with microplate readers. *PLoS ONE* 16: e0245205

Lajoie MJ, Rovner AJ, Goodman DB, Aerni HR, Haimovich AD, Kuznetsov G, Mercer JA, Wang HH, Carr PA, Mosberg JA *et al* (2013) Genomically recoded organisms expand biological functions. *Science* 342: 357–360

Lee PC, Bochner BR, Ames BN (1983) Diadenosine 5′,5‴-P1,P4-tetraphosphate and related adenylylated nucleotides in salmonella typhimurium. *J Biol Chem* 258: 6827–6834

Lee S, Oh S, Yang A, Kim J, Soll D, Lee D, Park HS (2013) A facile strategy for selective incorporation of phosphoserine into histones. *Angew Chem Int Ed Engl* 52: 5771–5775

Lenhard B, Orellana O, Ibba M, Weygand-Durasevic I (1999) tRNA recognition and evolution of determinants in seryl-tRNA synthesis. *Nucleic Acids Res* 27: 721–729

Lin-Chao S, Chen WT, Wong TT (1992) High copy number of the pUC plasmid results from a rom/Rop-suppressible point mutation in RNA II. *Mol Microbiol* 6: 3385–3393

Ling J, Reynolds N, Ibba M (2009) Aminoacyl-tRNA synthesis and translational quality control. *Annu Rev Microbiol* 63: 61–78

Liu CC, Schultz PG (2010) Adding new chemistries to the genetic code. *Annu Rev Biochem* 79: 413–444

Lu KV, Rohde MF, Thomason AR, Kenney WC, Lu HS (1995) Mistranslation of a TGA termination codon as tryptophan in recombinant platelet-derived growth factor expressed in *Escherichia coli*. *Biochem J* 309: 411–417

Manickam N, Nag N, Abbasi A, Patel K, Farabaugh PJ (2014) Studies of translational misreading in vivo show that the ribosome very efficiently discriminates against most potential errors. *RNA* 20: 9–15

Martinis SA, Boniecki MT (2010) The balance between pre- and post-transfer editing in tRNA synthetases. *FEBS Lett* 48: 455–459

Miguel A, Zietek M, Shi H, Sueki A, Maier L, Verheul J, Blaauwen TD, Valen DV, Typas A, Huang KC (2022) Modulation of bacterial cell size and growth rate via activation of a cell envelope stress response. *bioRxiv* https://doi.org/10.1101/2022.07.26.501648 [PREPRINT]

Mohler K, Aerni HR, Gassaway B, Ling J, Ibba M, Rinehart J (2017a) MS-READ: quantitative measurement of amino acid incorporation. *Biochim Biophys Acta* 1861: 3081–3088

Mohler K, Mann R, Bullwinkle TJ, Hopkins K, Hwang L, Reynolds NM, Gassaway B, Aerni HR, Rinehart J, Polymenis M *et al* (2017b) Editing of misaminoacylated tRNA controls the sensitivity of amino acid stress responses in *Saccharomyces cerevisiae*. *Nucleic Acids Res* 45: 3985–3996

Mohler K, Mann R, Ibba M (2017c) Isoacceptor specific characterization of tRNA aminoacylation and misacylation in vivo. *Methods* 113: 127–131

Mukai T, Hoshi H, Ohtake K, Takahashi M, Yamaguchi A, Hayashi A, Yokoyama S, Sakamoto K (2015) Highly reproductive *Escherichia coli* cells with no specific assignment to the UAG codon. *Sci Rep* 5: 9699

Nameki N, Asahara H, Hasegawa T (1996) Identity elements of *Thermus thermophilus* tRNA(Thr). *FEBS Lett* 396: 201–207

Nameki N, Tamura K, Asahara H, Hasegawa T (1997) Recognition of tRNA (Gly) by three widely diverged glycyl-tRNA synthetases. *J Mol Biol* 268: 640–647

O'Connor M (1998) tRNA imbalance promotes −1 frameshifting via near-cognate decoding. *J Mol Biol* 279: 727–736

Pang YL, Poruri K, Martinis SA (2014) tRNA synthetase: tRNA aminoacylation and beyond. *Wiley Interdiscip Rev RNA* 5: 461–480

Park HS, Hohn MJ, Umehara T, Guo LT, Osborne EM, Benner J, Noren CJ, Rinehart J, Soll D (2011) Expanding the genetic code of *Escherichia coli* with phosphoserine. *Science* 333: 1151–1154

Patange O, Schwall C, Jones M, Villava C, Griffith DA, Phillips A, Locke JCW (2018) *Escherichia coli* can survive stress by noisy growth modulation. *Nat Commun* 9: 5333

Perez-Riverol Y, Csordas A, Bai J, Bernal-Llinares M, Hewapathirana S, Kundu DJ, Inuganti A, Griss J, Mayer G, Eisenacher M *et al* (2019) The PRIDE database and related tools and resources in 2019: improving support for quantification data. *Nucleic Acids Res* 47: D442–D450

Pirman NL, Barber KW, Aerni HR, Ma NJ, Haimovich AD, Rogulina S, Isaacs FJ, Rinehart J (2015) A flexible codon in genomically recoded *Escherichia coli* permits programmable protein phosphorylation. *Nat Commun* 6: 8130

© 2023 The Authors

Pramanik J, Keasling JD (1997) Stoichiometric model of *Escherichia coli* metabolism: incorporation of growth-rate dependent biomass composition and mechanistic energy requirements. *Biotechnol Bioeng* 56: 398–421

Rauhut R, Gabius HJ, Engelhardt R, Cramer F (1985) Archaebacterial phenylalanyl-tRNA synthetase. Accuracy of the phenylalanyl-tRNA synthetase from the archaebacterium *Methanosarcina barkeri*, Zn(II)-dependent synthesis of diadenosine 5′,5‴-P1,P4-tetraphosphate, and immunological relationship of OFFnylalanyl-tRNA synthetases from different urkingdoms. *J Biol Chem* 260: 182–187

Rogerson DT, Sachdeva A, Wang K, Haq T, Kazlauskaite A, Hancock SM, Huguenin-Dezot N, Muqit MM, Fry AM, Bayliss R *et al* (2015) Efficient genetic encoding of phosphoserine and its nonhydrolyzable analog. *Nat Chem Biol* 11: 496–503

Roy H, Ling J, Irnov M, Ibba M (2004) Post-transfer editing in vitro and in vivo by the beta subunit of phenylalanyl-tRNA synthetase. *EMBO J* 23: 4639–4648

Roy B, Leszyk JD, Mangus DA, Jacobson A (2015) Nonsense suppression by near-cognate tRNAs employs alternative base pairing at codon positions 1 and 3. *Proc Natl Acad Sci USA* 112: 3038–3043

Sander T, Farke N, Diehl C, Kuntz M, Glatter T, Link H (2019) Allosteric feedback inhibition enables robust amino acid biosynthesis in *E. coli* by enforcing enzyme overabundance. *Cell Syst* 8: 66–75

Schneider CA, Rasband WS, Eliceiri KW (2012) NIH image to ImageJ: 25 years of image analysis. *Nat Methods* 9: 671–675

Sherman JM, Rogers MJ, Soll D (1992) Competition of aminoacyl-tRNA synthetases for tRNA ensures the accuracy of aminoacylation. *Nucleic Acids Res* 20: 2847–2852

Singaravelan B, Roshini BR, Munavar MH (2010) Evidence that the supE44 mutation of *Escherichia coli* is an amber suppressor allele of glnX and that it also suppresses ochre and opal nonsense mutations. *J Bacteriol* 192: 6039–6044

Standley MS, Million-Weaver S, Alexander DL, Hu S, Camps M (2019) Genetic control of ColE1 plasmid stability that is independent of plasmid copy number regulation. *Curr Genet* 65: 179–192

Starosta AL, Lassak J, Jung K, Wilson DN (2014) The bacterial translation stress response. *FEMS Microbiol Rev* 38: 1172–1201

Steinfeld JB, Aerni HR, Rogulina S, Liu Y, Rinehart J (2014) Expanded cellular amino acid pools containing phosphoserine, phosphothreonine, and phosphotyrosine. *ACS Chem Biol* 9: 1104–1112

Swanson R, Hoben P, Sumner-Smith M, Uemura H, Watson L, Soll D (1988) Accuracy of in vivo aminoacylation requires proper balance of tRNA and aminoacyl-tRNA synthetase. *Science* 242: 1548–1551

Tomizawa J, Som T (1984) Control of ColE1 plasmid replication: enhancement of binding of RNA I to the primer transcript by the rom protein. *Cell* 38: 871–878

Wang Z, Xiang L, Shao J, Wegrzyn A, Wegrzyn G (2006) Effects of the presence of ColE1 plasmid DNA in *Escherichia coli* on the host cell metabolism. *Microb Cell Fact* 5: 34

Wang K, Neumann H, Peak-Chew SY, Chin JW (2007) Evolved orthogonal ribosomes enhance the efficiency of synthetic genetic code expansion. *Nat Biotechnol* 25: 770–777

Wegrzyn G (1999) Replication of plasmids during bacterial response to amino acid starvation. *Plasmid* 41: 1–16

Wick LM, Egli T (2004) Molecular components of physiological stress responses in *Escherichia coli*. *Adv Biochem Eng Biotechnol* 89: 1–45

Yao Z, Davis RM, Kishony R, Kahne D, Ruiz N (2012) Regulation of cell size in response to nutrient availability by fatty acid biosynthesis in *Escherichia coli*. *Proc Natl Acad Sci USA* 109: E2561–E2568

Zamecnik PC, Stephenson ML, Janeway CM, Randerath K (1966) Enzymatic synthesis of diadenosine tetraphosphate and diadenosine triphosphate with a purified lysyl-sRNA synthetase. *Biochem Biophys Res Commun* 24: 91–97

Zhu M, Dai X (2019) Growth suppression by altered (p)ppGpp levels results from non-optimal resource allocation in *Escherichia coli*. *Nucleic Acids Res* 47: 4684–4693

License: This is an open access article under the terms of the Creative Commons Attribution License, which permits use, distribution and reproduction in any medium, provided the original work is properly cited.

© 2023 The Authors

Expanded View Figures

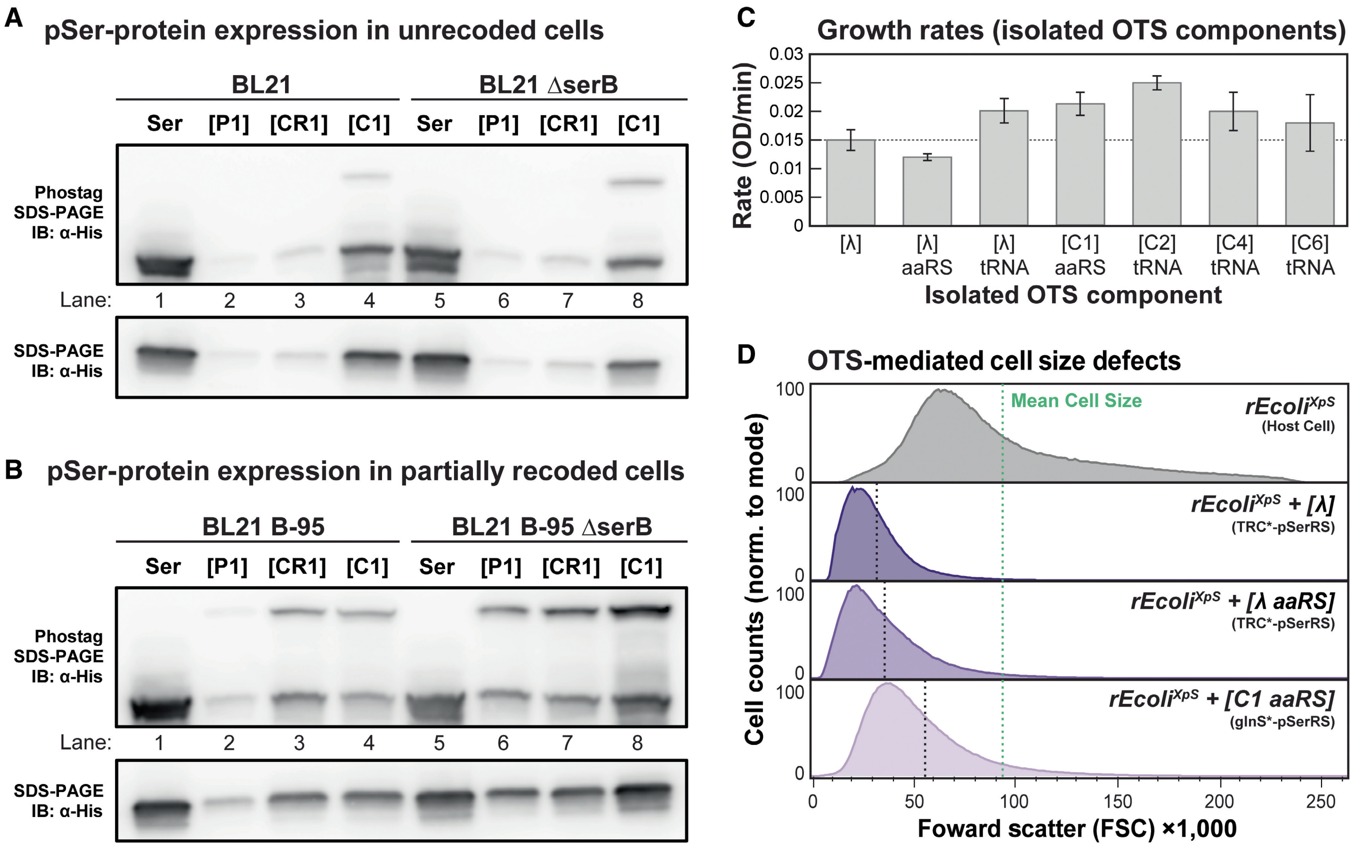

Figure EV1. Genomic recoding status and individual OTS component expression alter OTS performance and cellular fitness.

A–D The phosphoprotein yield and fidelity from non-recoded cells expressing 1× tRNA OTS variants as a function of plasmid copy number (**[P1]**, **[CR1]**, and **[C1]**) were assessed qualitatively by immunoblot analysis of an E(17)TAG-GFP-6×His reporter protein. Crude lysate was separated by SDS–PAGE with and without Phos–tag™ reagent (for phosphoprotein separation) and visualized by immunoblot against the 6xHis epitope tag. Expression and fidelity were assessed in BL21 and BL21 ΔserB (A) and in partially recoded BL21 B-95 and BL21 B-95 ΔserB (B). Each lane was loaded with 7.5 μl of O.D.-normalized cell lysate. Growth rate analysis for host cells (rEcoli^{XpS}) expressing **[λ]** or individual OTS component, as indicated. The growth rate was calculated from continuous monitoring of the optical density (absorbance at 600 nm) as a function of time and averaged across three biological replicates; error bars represent 1 SD, and the horizontal dashed line indicates the average growth rate of cells expressing **[λ]** (C). *E. coli* cell size was measured by flow cytometry with forward light scattering (FSC) used as a proxy for cell size. The size of host rEcoli^{XpS} cells and cells expressing **[λ]** or high-**[λ aaRS]** or low-**[C1 aaRS]** toxicity OTS components were recorded and plotted as histograms representing individual FSC values by abundance in the population. Comparison of population distributions highlights cell size differences across the sample populations. Visualization of cell size distribution and determination of mean cell size (vertical dashed lines) from one million discrete cell measurements was conducted using FlowJo (D).

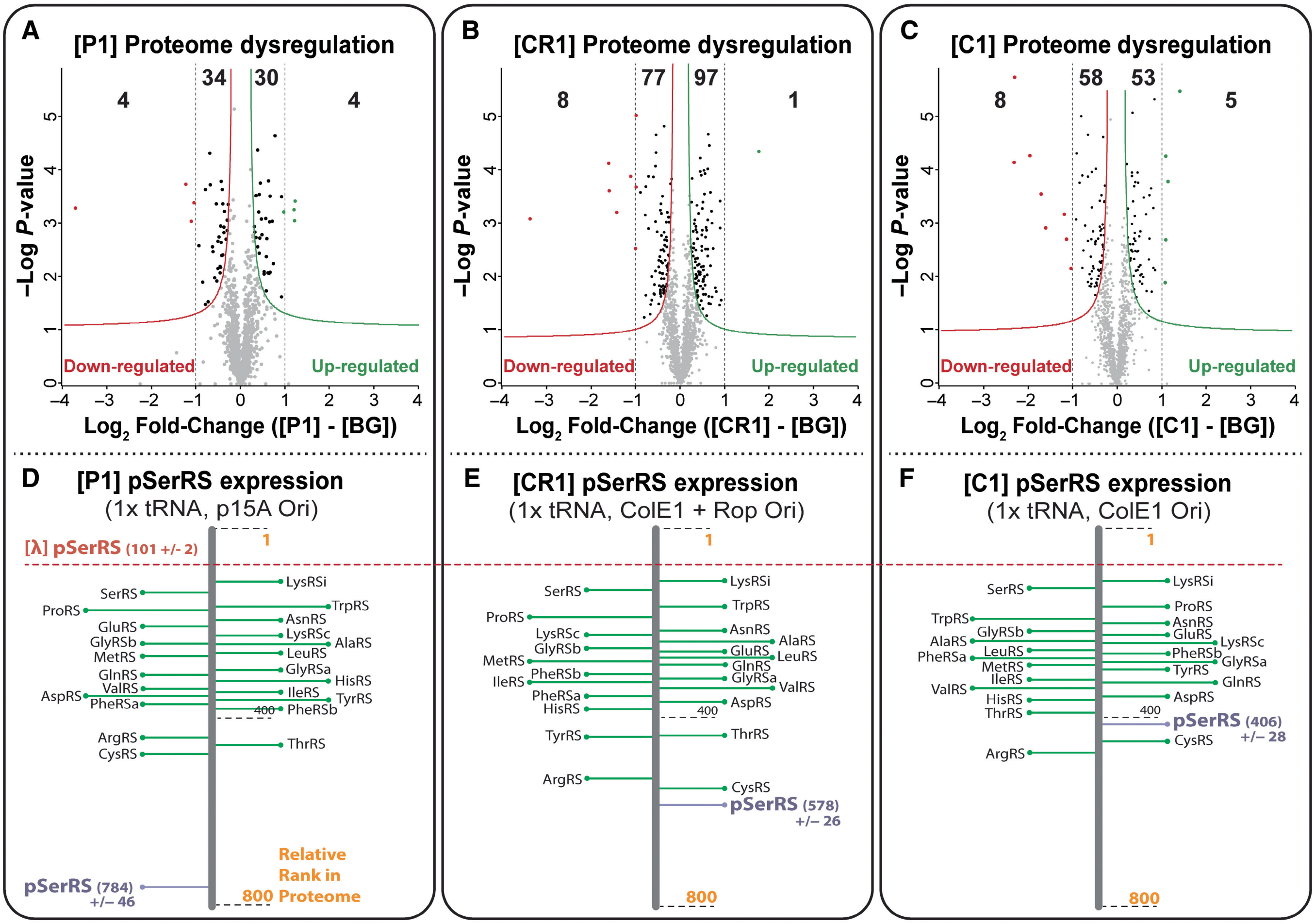

Figure EV2. Plasmid origin of replication contributes to pSerRS-dependent proteome dysregulation.

A–F Proteome composition was determined by mass spectrometry from host cells expressing identical OTS variants aside from their origins of replication; p15a **[P1]** (A), ColE1 + Rop **[CR1]** (B), or ColE1 **[C1]** (C). Proteomic dysregulation and statistical analysis were conducted in Perseus and illustrated as a volcano plot comparing differences in individual protein expression to the transformed *P*-value of the same differentially expressed protein. Statistically significant dysregulated proteins were identified by pair-wise *t*-test ($P < 0.05$) and FDR (0.1) calculation from three independent biological replicates and fell outside the asymptotic lines. From the same proteomes, relative protein abundance was assigned based on rank-ordered, label-free iBAQ protein quantification scores determined by MaxQuant analysis software and used to annotate the abundance of pSerRS (purple line) relative to host *E. coli* aaRSs (green lines) and pSerRS abundance from host cells expressing **[λ]** (red dashed line); p15a **[P1]** (D), ColE1 + Rop **[CR1]** (E), or ColE1 **[C1]** (F).

 © 2023 The Authors

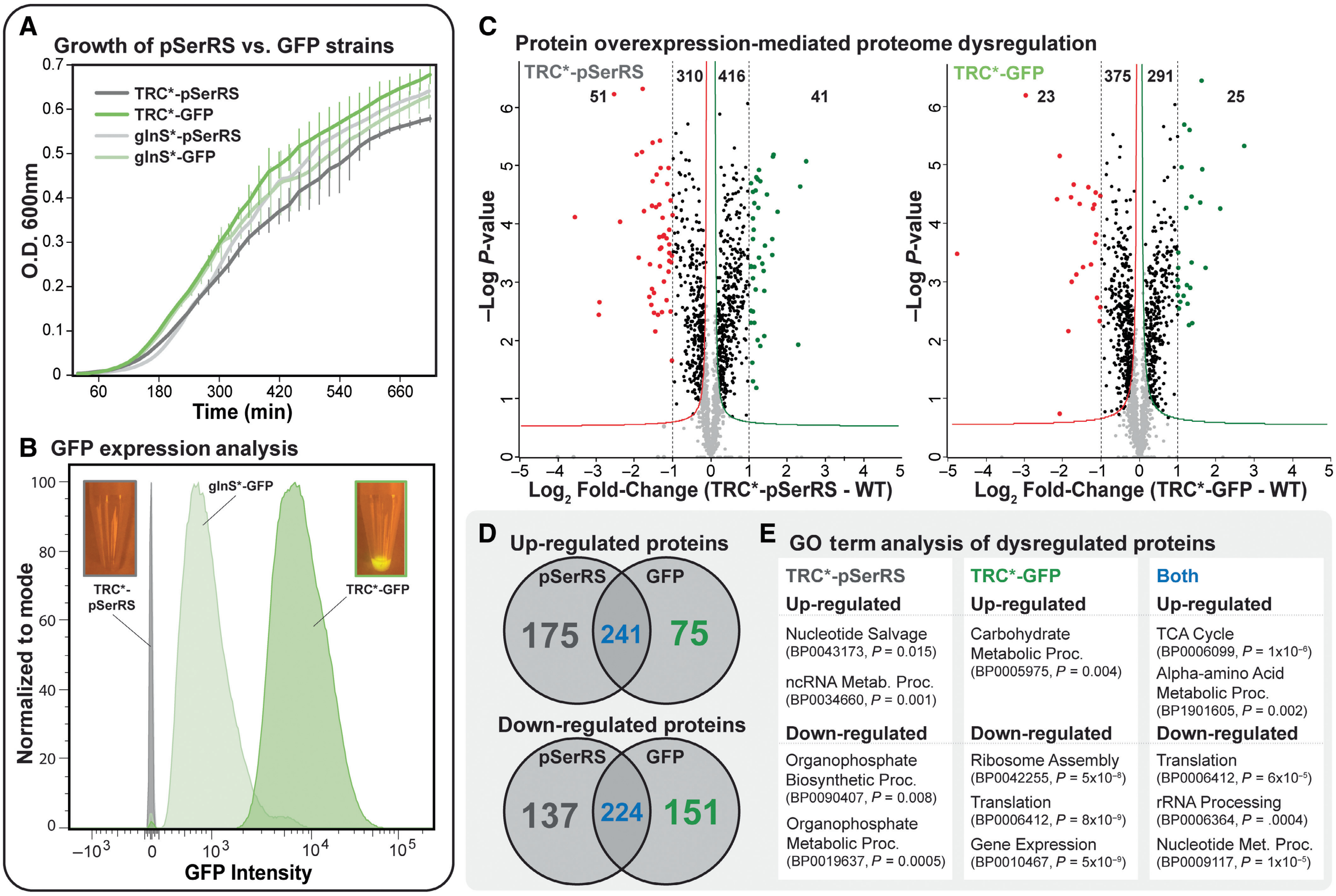

Figure EV3. Identification of pSerRS-specific proteome dysregulation.

A–E Growth fitness defects resulting from overexpression of pSerRS or a non-aaRS protein (GFP) were identified following analysis of kinetic growth metrics derived from continuous monitoring of the optical density (absorbance at 600 nm) as a function of time in host cells with high-level expression of TRC*-pSerRS (dark gray) or TRC*-GFP (dark green) and low-level expression of glnS*-pSerRS (light gray) or glnS*-GFP (light green). Reported values for specific growth rates represent the mean and SD from three biological replicates; growth curve error bars reflect the SD of discrete O.D. measurements (A). Flow cytometry analysis of $1e10^6$ cell to verify GFP expression and intensity across a population of host cells with high-level expression of TRC*-pSerRS (dark gray) or TRC*-GFP (dark green) and low-level expression of glnS*-pSerRS (light gray) or glnS*-GFP (light green). Data analysis and visualization were conducted using FlowJo analysis software and illustrated as an overlay of log scale GFP intensity normalized to individual sample mode (B). Proteome composition was determined by mass spectrometry from host cells expressing either TRC*-pSerRS or TRC*-GFP from identical expression vectors. Proteomic dysregulation and statistical analysis were conducted in Perseus and illustrated as a volcano plot displaying differences in individual protein expression across the pSerRS proteome relative to the proteome of cells expressing GFP. Statistically significant dysregulated proteins were identified by pair-wise *t*-test ($P < 0.05$) and FDR (0.1) calculation from three independent biological replicates and fell outside the asymptotic lines (C). Dysregulated proteins from (C) were compared to identify pSerRS-(gray), GFP-specific (green), and overlapping proteins (blue) relative to the host cell background (D). Extended GO term enrichment analysis of unique and common dysregulated proteins was conducted stringDB and Pathway Tools to identify statistically significant functional pathway and biological process enrichments (*P*-values as indicated) and define the common core response (CCP) to protein overexpression (E). All the data were collected from three independent biological replicated unless otherwise noted.

© 2023 The Authors

System-wide Optimization of an Orthogonal Translation System with Enhanced Biological Tolerance

Kyle Mohler[1,2], Jack M. Moen[3,4], Svetlana Rogulina[1,2], and Jesse Rinehart[1,2]

[1]Department of Cellular & Molecular Physiology, Yale School of Medicine, New Haven, CT 06520, USA

[2]Systems Biology Institute, Yale University, New Haven, CT 06516, USA

[3]Quantitative Biosciences Institute (QBI), University of California, San Francisco; San Francisco, CA 94158, USA. 2QBI Coronavirus Research Group (QCRG); San Francisco, CA 94158, USA.

[4]Department of Cellular and Molecular Pharmacology, University of California, San Francisco, San Francisco, CA 94158, USA.

Appendix Figures and Tables

Correspondence should be addressed to jesse.rinehart@yale.edu

Appendix Contents **Page Number**

Appendix Figure S1 ……………………………………………………………………………….. 3
Appendix Table S1 …….………………………………………………………………………. 4
Appendix Figure S2 ……………………………………………………………………………. 5
Appendix Figure S3 ……………………………………………………………………………… 6
Appendix Figure S4 ……………………………………………………………………………… 7
Appendix Figure S5 ……………………………………………………………………………… 8
Appendix Figure S6 ……………………………………………………………………………… 9
Appendix Figure S7 ………..…..……………………………………………………………….. 10
Appendix Figure S8 ………..……..…………………………………………………………….. 11
Appendix Table S2 …………..………………………………………………………………..…. 12
Appendix Figure S9 …………..…...…………………………………………………………….. 13
Appendix Table S3 ………………..………………………………………………………….…. 14
Appendix References …………….……………………………………………………………… 15

Appendix Figure S1:

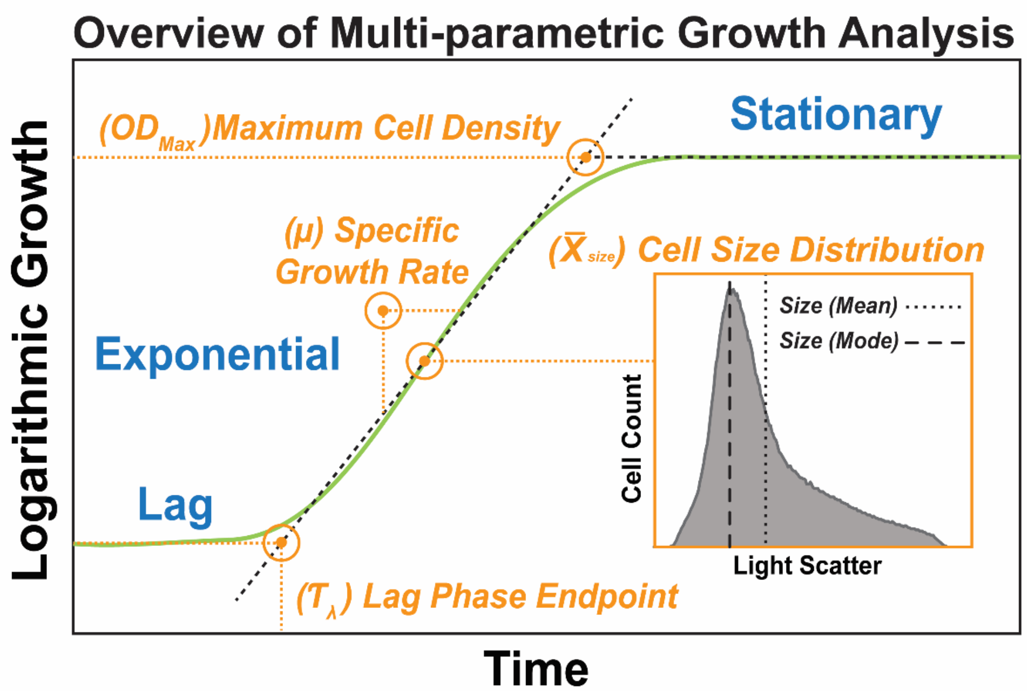

Appendix Figure S1: Graphical overview of growth analysis parameters. Cellular growth phenotypes were assessed by temporal analysis of population growth and cell size properties. Cellular growth was monitored through kinetic measurement of optical density (absorbance at 600 nm) and divided into three discrete parameters - lag phase endpoint ($\boldsymbol{T_{\lambda}}$), specific growth rate ($\boldsymbol{\mu}$), and growth efficiency (maximum cell density, $\boldsymbol{OD_{Max}}$) – using PreCog analysis software. Cell size was measured by flow cytometry using forward scatter (FSC) as a cell-size proxy. The population cell size distribution ($\boldsymbol{\bar{X}_{size}}$) was analyzed using statistical parameters (mean and modal cell size) determined using FlowJo analysis software.

Appendix Table S1:

Appendix Table S1: Cellular growth parameters used to assess growth phenotypes and relative fitness

Host Cell	Ori. of Rep.	tRNA Copy Number	Growth Rate (*μ*)	*S.D.*	Lag Time (min., T_λ)	*S.D.*	Max Cell Density ($O.D._{Max}$)	*S.D.*	Cell Size (Mean, $\bar{X}$)	Cell Size (Mode)	*C.V.*	Relative Fitness
rEcoli^{XpS}	-	-	0.040	0.007	71	10	3.58	0.47	93155	62464	48.2	1.00
rEcoli^{XpS}	pSerOTSλ	5x tRNA	0.015	0.002	210	6	1.06	0.02	31255	20480	49.2	0.34
rEcoli^{XpS}	p15a	1x tRNA	0.028	0.007	74	10	2.75	0.04	28456	19456	67.5	0.73
rEcoli^{XpS}	p15a	2x tRNA	0.022	0.001	86	7	2.49	0.03	46596	24576	71.3	0.64
rEcoli^{XpS}	p15a	4x tRNA	0.019	0.002	81	6	2.56	0.05	37565	21504	70.7	0.62
rEcoli^{XpS}	p15a	6x tRNA	0.015	0.002	90	1	2.71	0.12	34593	20480	61	0.57
rEcoli^{XpS}	ColE1 + Rop	1x tRNA	0.026	0.004	86	11	1.31	0.04	43429	25600	55	0.58
rEcoli^{XpS}	ColE1 + Rop	2x tRNA	0.026	0.004	81	3	1.26	0.02	42267	27648	55.3	0.59
rEcoli^{XpS}	ColE1 + Rop	4x tRNA	0.021	0.001	111	18	1.26	0.04	45235	30720	56.1	0.50
rEcoli^{XpS}	ColE1 + Rop	6x tRNA	0.028	0.003	85	8	1.29	0.07	60398	45056	49.1	0.64
rEcoli^{XpS}	ColE1	1x tRNA	0.039	0.001	72	5	2.80	0.01	63108	41984	70.1	0.85
rEcoli^{XpS}	ColE1	2x tRNA	0.022	0.001	106	1	2.42	0.01	35997	15360	105	0.57
rEcoli^{XpS}	ColE1	4x tRNA	0.014	0.001	132	9	2.46	0.07	36570	21504	63.1	0.49
rEcoli^{XpS}	ColE1	6x tRNA	0.004	0.001	242	40	1.51	0.03	81828	41984	64.4	0.40

Host Cell	Ori. of Rep.	Isolated Component	Growth Rate (*μ*)	*S.D.*	Lag Time (T_λ)	*S.D.*	Max Cell Density ($O.D._{Max}$)	*S.D.*	Cell Size (Mean, $\bar{X}$)	Cell Size (Mode)	*C.V.*	Relative Fitness
rEcoli^{XpS}	ColE1	glnS*-pSerRS	0.021	0.002	79	3	1.63	0.03	52645	38912	49	0.61
rEcoli^{XpS}	ColE1 + Rop	TRC*-pSerRS	0.012	0.001	279	97	1.13	0.02	36565	22528	59.2	0.32
rEcoli^{XpS}	ColE1	2x tRNA Only	0.025	0.001	113	18	1.39	0.05	*N.D.*	*N.D.*	-	0.55
rEcoli^{XpS}	ColE1	4x tRNA Only	0.020	0.003	121	16	1.19	0.01	*N.D.*	*N.D.*	-	0.48
rEcoli^{XpS}	ColE1	6x tRNA Only	0.018	0.005	140	24	1.04	0.01	*N.D.*	*N.D.*	-	0.42
rEcoli^{XpS}	ColE1 + Rop	λ tRNA Only	0.020	0.002	96	14	1.06	0.02	*N.D.*	*N.D.*	-	0.51

Host Cell	Ori. of Rep.	tRNA Copy Number	Growth Rate (*μ*)	*S.D.*	Lag Time (T_λ)	*S.D.*	Max Cell Density ($O.D._{Max}$)	*S.D.*	Cell Size (Mean, $\bar{X}$)	Cell Size (Mode)	*C.V.*	Relative Fitness
BL21	-	-	0.057	0.003	72	12	3.42	0.23	*N.D.*	*N.D.*	-	1.00
BL21	p15a	1x tRNA	0.033	0.002	73	4	1.16	0.03	*N.D.*	*N.D.*	-	0.63
BL21	p15a	2x tRNA	0.048	0.004	79	4	2.76	0.47	*N.D.*	*N.D.*	-	0.85
BL21	p15a	4x tRNA	0.041	0.001	85	1	2.48	0.05	*N.D.*	*N.D.*	-	0.76
BL21	p15a	6x tRNA	0.039	0.002	84	1	2.50	0.11	*N.D.*	*N.D.*	-	0.76
BL21	ColE1 + Rop	1x tRNA	0.027	0.001	70	0	1.32	0.03	*N.D.*	*N.D.*	-	0.63
BL21	ColE1 + Rop	2x tRNA	0.022	0.001	73	4	1.16	0.03	*N.D.*	*N.D.*	-	0.57
BL21	ColE1 + Rop	4x tRNA	*N.V.*	*N.V.*	*N.V.*	*N.V.*	*N.V.*	*N.V.*	*N.V.*	*N.V.*	*N.V.*	*N.V.*
BL21	ColE1 + Rop	6x tRNA	*N.V.*	*N.V.*	*N.V.*	*N.V.*	*N.V.*	*N.V.*	*N.V.*	*N.V.*	*N.V.*	*N.V.*
BL21	ColE1	1x tRNA	0.025	0.001	79	2	1.19	0.03	*N.D.*	*N.D.*	-	0.57
BL21	ColE1	2x tRNA	0.030	0.001	119	3	2.17	0.06	*N.D.*	*N.D.*	-	0.59
BL21	ColE1	4x tRNA	*N.V.*	*N.V.*	*N.V.*	*N.V.*	*N.V.*	*N.V.*	*N.V.*	*N.V.*	*N.V.*	*N.V.*
BL21	ColE1	6x tRNA	*N.V.*	*N.V.*	*N.V.*	*N.V.*	*N.V.*	*N.V.*	*N.V.*	*N.V.*	*N.V.*	*N.V.*

N.D. **(Not Determined); N.V. (Not Viable)**

Appendix Figure S2:

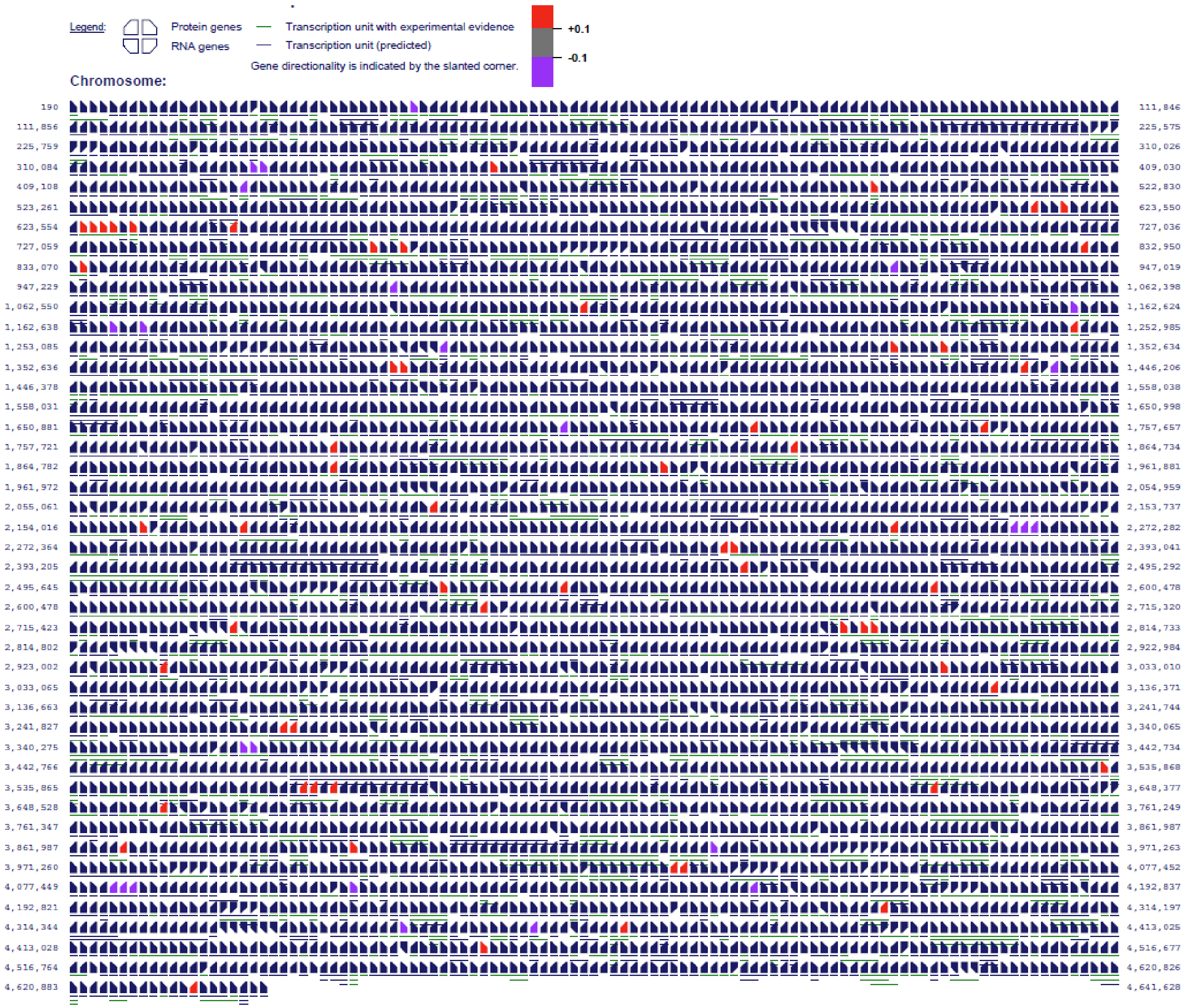

Appendix Figure S2: Alterations to proteome composition mediated by low-toxicity [C1]. Proteomic analysis of rEcoli^{XpS} cells with and without expression from **[C1]** was conducted using Perseus. Proteins with statistically significant up-regulation (red) or down-regulation (purple) were matched to their corresponding gene in relation to the complete *E. coli* genome (navy) using Pathway Tools. Proteomes were obtained in triplicate and statistical significance was determined by t-test with p<0.05.

Appendix Figure S3:

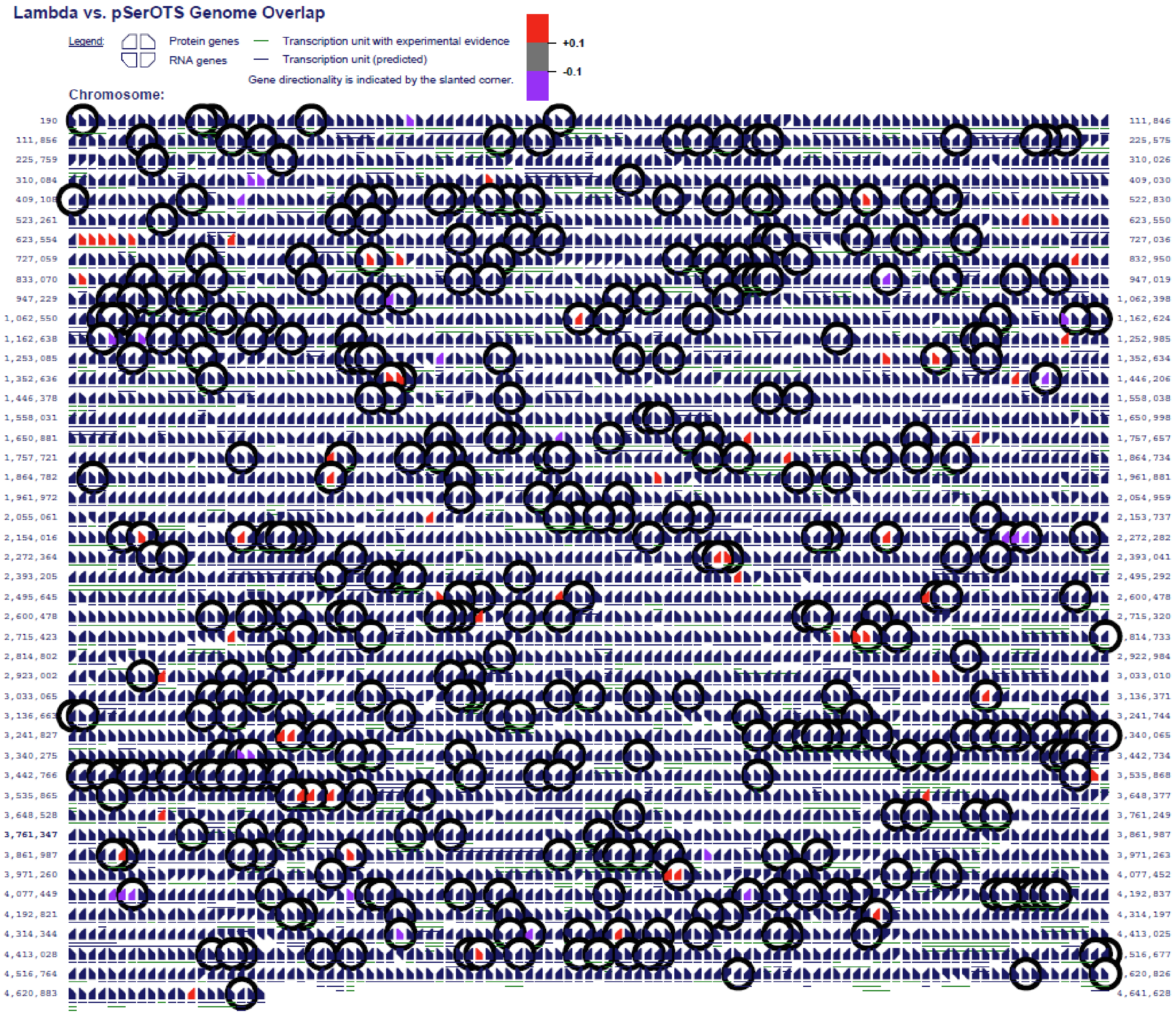

Appendix Figure S3: Alterations to proteome composition mediated by high-toxicity [λ]. Proteomic analysis of rEcoli^{XpS} cells with and without expression from **[λ]** was conducted using Perseus. Proteins with statistically significant up- or down-regulation in the presence of **[λ]** are indicated with black circles, and for **[C1]** up-regulation (red) or down-regulation (purple). All proteins were matched to their corresponding gene in relation to the complete *E. coli* genome (navy) using Pathway Tools. Proteomes were obtained in triplicate and statistical significance was determined by t-test with $p<0.05$.

Appendix Figure S4:

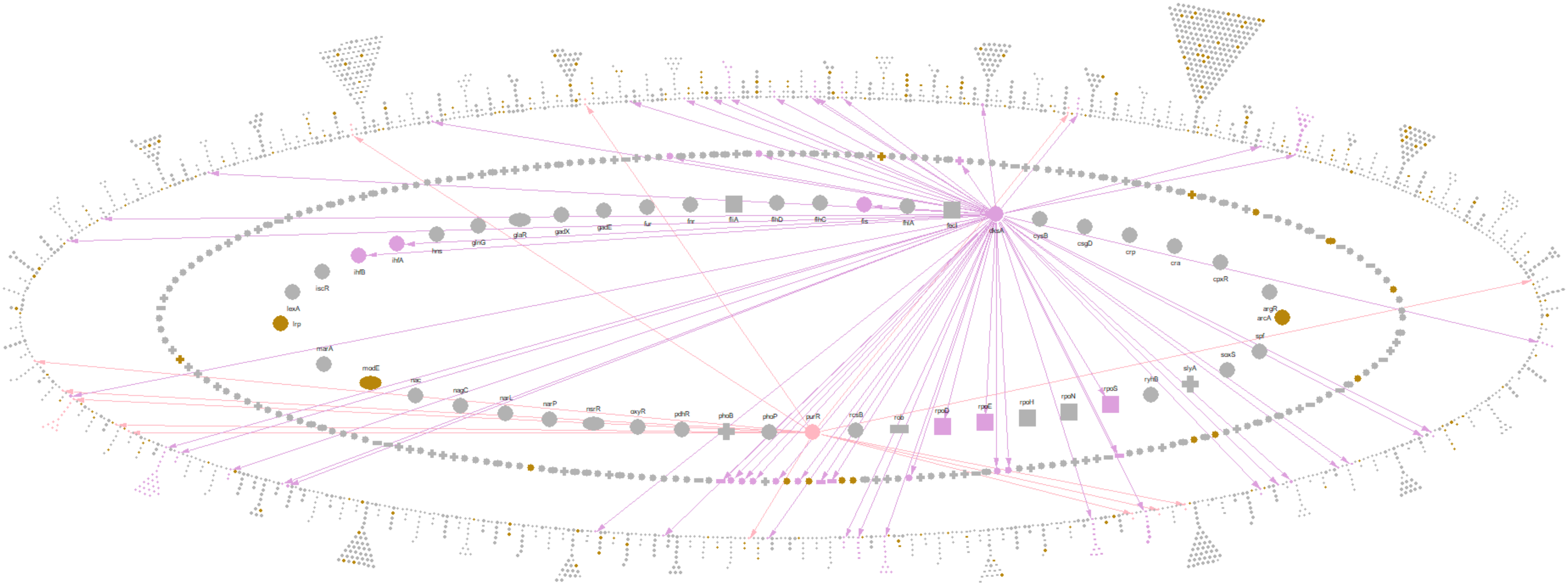

Appendix Figure S4: Expression from high-toxicity [λ] OTS perturbs transcriptional regulation in genomically recoded cells. Proteome dysregulation in cells expressing **[λ]** was mapped to the *E. coli* transcriptional regulatory network using Pathway Tools. Two of the most affected regulons (controlled by DskA and PurR) are highlighted in purple and pink, respectively. The corresponding color-coded arrows and nodes constitute dysregulated interactions across respective regulons. Nodes colored burnt yellow indicate statistically significant change in the protein abundance for that specific transcriptional regulator within cells expressing **[λ]**, relative to to WT. Protein abundance was assessed by mass spectrometry from at least three independent biological replicates and analyzed using MaxQuant. Statistical analysis was performed in Perseus software using an FDR of 0.01 and $p < 0.05$.

Appendix Figure S5:

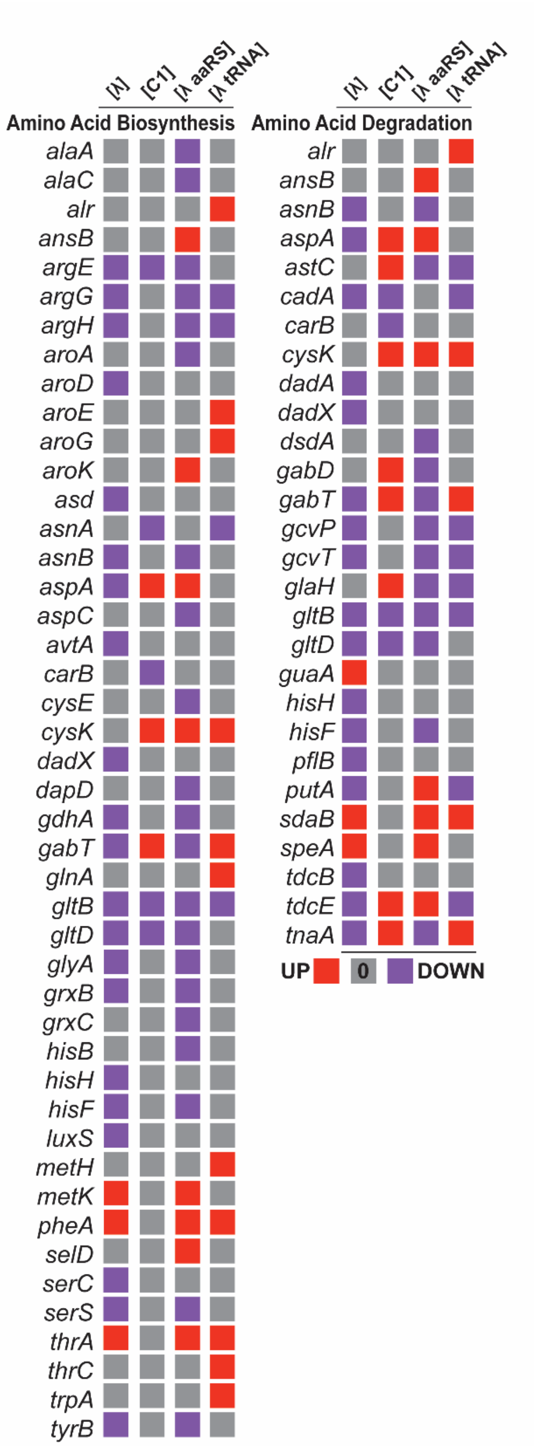

Appendix Figure S5: Isolated o-tRNA expression from [λ tRNA] displays characteristics of stringent response activation. Cells expressing only o-tRNA from **[λ tRNA]** were subjected to proteomic analysis and compared to cells expressing full (**[λ]** and **[C1]**) and partial (**[λ aaRS]**) OTS variants. Pathway enrichment analysis was conducted for strains expressing OTS variants using Pathway Tools. Dysregulated pathway components are highlighted for specific genes with the enriched pathway, with up-regulated proteins in red, down-regulated proteins in purple, and proteins with no change compared to WT host cells in grey. Enrichment cutoffs were set to a statistically significant differential expression score of 0.1. All proteomes were quantified in triplicate and analyzed in Perseus using t-test and volcano plot functions to obtain statistically significant proteomic deviations.

Appendix Figure S6:

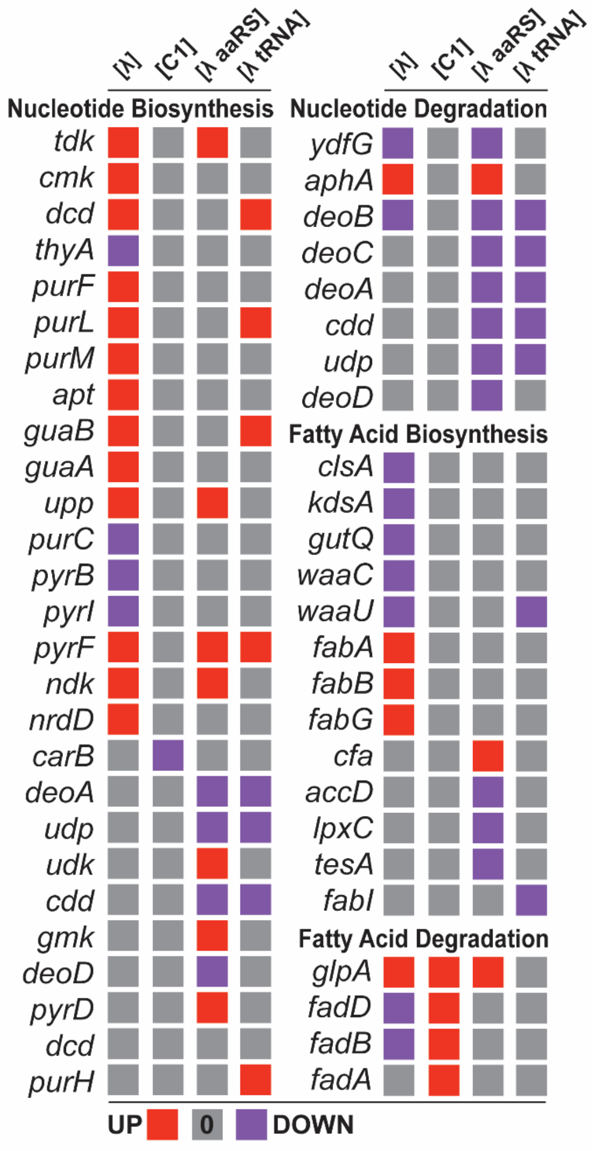

Appendix Figure S6: o-aaRS overexpression mediated dysregulation of nucleotide biosynthesis. Proteomic dysregulation from cells expressing full (**[λ]** and **[C1]**) and partial (**[λ tRNA]** and **[λ aaRS]**) OTS variants was assessed by mass spectrometry. Pathway enrichment analysis was conducted for strains expressing OTS variants using Pathway Tools. Dysregulated pathway components are highlighted for specific genes with the enriched pathway, with up-regulated proteins in red, down-regulated proteins in purple, and proteins with no change compared to WT host cells in grey. Enrichment cutoffs were set to a statistically significant differential expression score of 0.1. All proteomes were quantified in triplicate and analyzed in Perseus using t-test and volcano plot functions to obtain statistically significant proteomic deviations.

Appendix Figure S7:

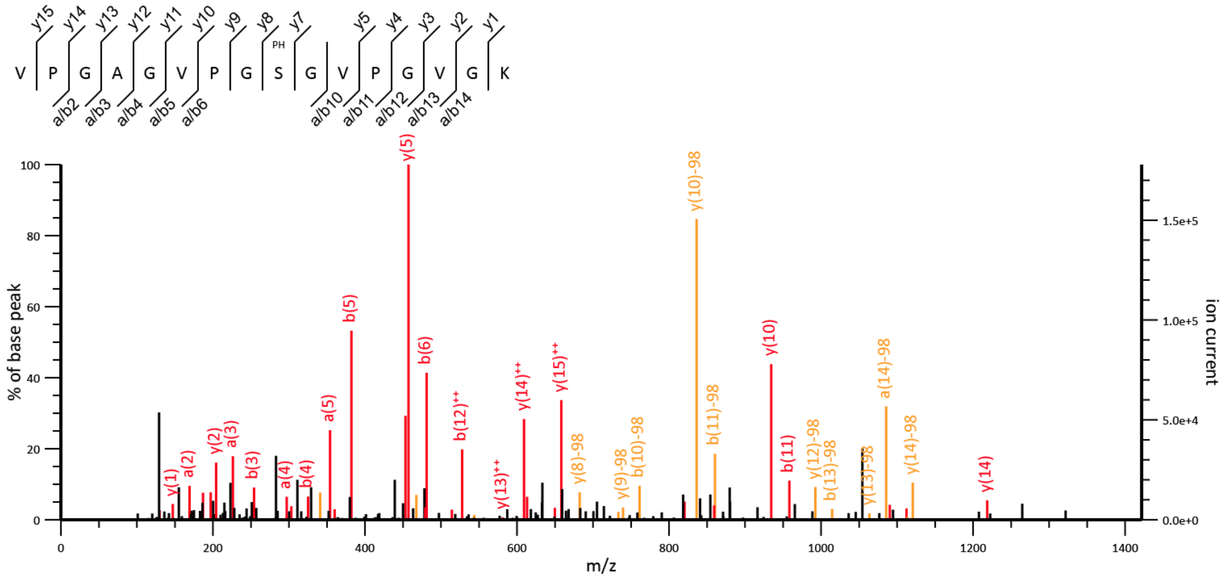

Appendix Figure S7: MS2 ion spectrum and peptide sequence confirming pSer misincorporation. MS-READ reporter proteins with a central Gly at the guest position were purified from rEcoli[XpS] host cells expressing TRC*-pSerRS alone. Raw mass spectrometry data was processed and searched using Mascot. High confidence identification and peptide sequencing for the Gly-MS-READ reporter peptide (precursor m/z 707.858 M^{++}) identified pSer misincorporation at the Gly codon, indicated by "PH" above Ser between y7 and y8.

Appendix Figure S8:

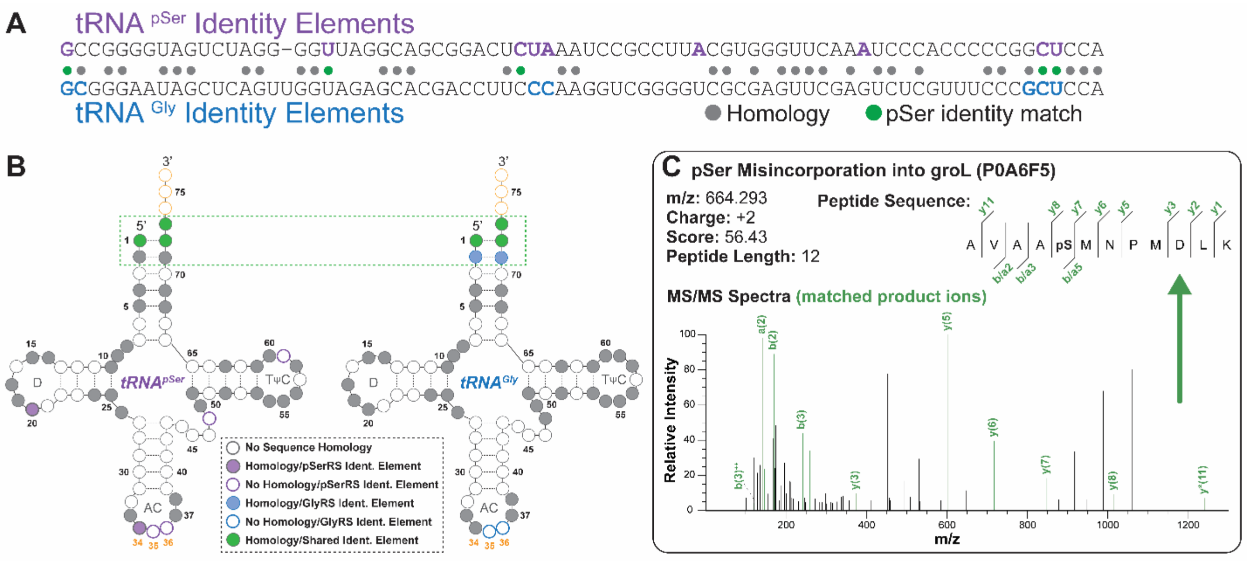

Appendix Figure S8: Proteome damage mediated by misidentification of tRNA^{pSer} by host aaRSs. pSerRS is able to recognize and productively attach pSer onto *E. coli* tRNA^{Gly} causing mistranslation of Gly codons and proteome damage. Misrecognition occurs due to overlapping tRNA identity elements. *E. coli* tRNA^{Gly} (blue) and tRNA^{pSer} (purple) were aligned to identify primary sequence homology (grey circles) and pSerRS recognition elements common to both tRNAs (green circles) (**A**) and mapped to respective tRNA secondary structures to highlight the canonical identity elements and visualize overlap (**B**). The proteomes of cells expressing **[λ]** and **[λ aaRS]** were analyzed for pSer misincorporation using custom modification parameters in Mascot software. The MS2 spectrum and sequence for example groL peptide (m/z 664.293 M^{++}) found in all samples is presented in (**C**).

Appendix Table S2:

Appendix Table S2: Peptides with Gly → pSer substitutions in the E. coli proteome

Sequence	Length	Protein	Gene	Score	Intensity	Missed Cleavages	MS/MS m/z	Charge	m/z	Retention Time
LNEL**G**LQFMQ**G**ARFWHVLDASA**G**K	24	P76329	yedP	52.541	1.33E+08	1	991.7761841	3	991.1053	93.45
DFKLKG**G**VLP**G**EQEIDTVR	19	Q46915	gudX	44.616	13255000	2	1193.029419	2	1192.533	101.89
**G**TL**G**QDVIDIRTLGSKGVFTFDP**G**FTSTASCESK	34	P0ABH7	gltA	42.241	6189200	2	1292.577148	3	1292.236	96.084
MT**G**IVKTFD**G**K	11	P0A976	cspF	52.482	3.22E+08	1	692.8035889	2	692.8028	71.94
VISEAVAAA**GG**KLQ	14	Q46800	xdhB	40.994	50758000	1	752.4145508	2	751.8488	79.704
IATLLLP**G**I**G**TIHDLK	16	P51020	mhpE	48.981	36999000	0	932.0004883	2	931.9933	106.06
T**G**L**G**RRIALILVK	13	P39414	ttdT	47.261	19178000	2	799.453186	2	799.4514	91.432
**G**ISLQVNAGEHIAIL**G**R	17	P23886	cydC	68.536	29870000	0	968.9760742	2	968.4763	86.927
IETLCRLT**G**K	10	P0A738	moaC	121.99	2.4E+08	1	642.8224487	2	642.8227	102.2
LTRPRT**G**NGPR	11	P0A8J8	rhlB	50.607		2	659.8416138	2	659.8406	68.065
LYSMYNSAFLDDLTKAM**G**R	19	P75801	yliF	40.962	33665000	1	765.0105591	3	764.3423	81.847
NL**G**QENFDAAEK	12	P25552	gppA	44.753	1.72E+08	0	716.7801514	2	716.7794	64.83
NQVLEKL**G**LNSEEQK	15	P18390	yjjA	48.741		1	913.4230957	2	913.427	65.93
NVELLT**G**FSNR	11	P76236	yeaI	47.869	30157000	0	672.8151855	2	672.8134	71.466
EPIKNEANN**G**LKNTR	15	P23869	ppiB	63.624	36029000	2	897.4296875	2	896.9331	84.654
AS**G**IPALPWEDCQ	13	P69330	citD	46.001	41994000	0	1537.645996	1	1537.639	98.059
M**G**KGKGNVEYWVALIQPGK	19	P0ADY7	rplP	52.693		2	1085.545166	2	1085.542	112.21
QVKTQSCVVA**G**KK	13	P39346	idnD	45.137		2	764.3868408	2	764.3837	68.082
AVAA**G**MNPMDLK	12	P0A6F5	groL	56.432	3005300	0	664.2930298	2	664.2929	91.465
ELESRQP**G**VR	10	P29131	ftsN	69.628	13653000	1	633.296814	2	633.2979	83.445
GETFA**G**FKQSR	11	P39452	nrdE	49.418		1	661.3015137	2	661.3005	78.103
IISPMT**G**YVSR	11	P27303	emrA	70.919	23921000	0	667.817749	2	667.3147	64.414
IV**G**YDEIFGRK	11	P30128	greB	41.029	17380000	1	695.8461304	2	695.842	87.754
QH**G**LQSMPRLRVMLNEK	17	P0AAK1	hycB	46.319	15848000	2	711.6933594	3	711.355	106.3
**G**MGESNPVTGNTCDNVK	17	P0A910	ompA	53.034	4607400	0	945.3768311	2	945.3739	73.828
IDLVQE**G**LR	9	P0AF06	motB	44.767	31797000	0	569.2811279	2	569.2812	65.859
INGATVDVRL**G**NKFR	15	P28248	dcd	48.44	18301000	2	878.9468384	2	878.4431	101.71
NTLEIVQE**G**VEAR	13	P08201	nirB	64.04	22226000	0	776.3782959	2	776.3744	83.343
SFEPL**G**LKK	9	P37197	yhjA	40.103	33542000	1	556.791687	2	556.7912	77.455
YR**G**VFGQRDVVFMSAK	16	P77561	ydeP	46.352	5.36E+08	2	986.4676514	2	985.9633	73.381
DEVILPYWRQLID**G**IK	16	P0AB89	purB	49.35	3072700	1	1027.5271	2	1027.024	114.44
IAENIKF**G**AAQ	11	P0AFX9	rseB	40.045	38493000	1	629.291626	2	629.2918	73.661
YL**G**RMHQTGR	10	P0C0K3	rdoA	53.17	3868400	1	657.7987061	2	657.2947	84.611

pSer substitution position in annotated as "**G**" in peptide sequence. Peptide in blue featured in Appendix Figure S8C.

Appendix Figure S9:

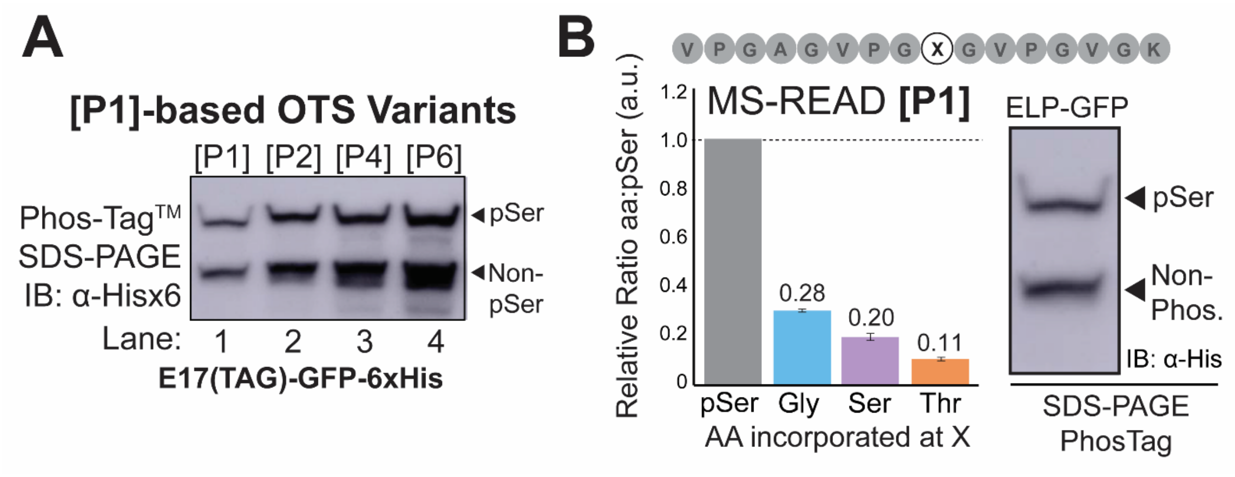

Appendix Figure S9: Reduced orthogonality of o-tRNA decreases fidelity in [P1]-based OTSs. The effect of plasmid copy number variation on OTS fidelity was assessed from cells expressing a UAG-containing reporter protein and an OTS variant (**[P1]**, **[P2]**, **[P4]**, **[P6]**) following separation by Phos-tag™ gel and immunoblot analysis; pSer-reporter protein (upper band), non-pSer-reporter protein (lower band) (**A**). Amino acid misincorporation at in cells expressing **[P1]** was assessed by mass spectrometry analysis of a UAG-containing MS-READ reporter. Amino acid incorporation events from three independent samples were quantified using Skyline and graphed relative to the level of pSer incorporation within the same sample (alongside enlargement of S9A, Lane 1 reproduced for reference as representative qualitative analysis sample) (**B**).

Appendix Table S3:

Appendix Table S3: E. coli strains and plasmids used in this study

Strains	Name	Growth Requirements	Reference
	BL21 (DE3)	No AB required	(Studier & Moffatt, 1986)
	BL21 (DE3) ΔserB	No AB required	(Steinfeld et al, 2014)
	BL21 (DE3) B-95	No AB required	(Mukai et al, 2015)
	BL21 (DE3) B-95 ΔserB	No AB required	(Zhu et al, 2019)
	C321.ΔA (C321 mutS+, λ-, Δ(ybhB-bioAB)::zeoR, ΔprfA)	Requires d-biotin	(Lajoie et al, 2013)
	rEcoli^{XpS} (C321 mutS+, λ-, Δ(ybhB-bioAB)::zeoR, ΔprfA, ΔserB)	Requires d-biotin	This Study
**Plasmids**	**Name**	**Growth Requirements**	**Reference**
E29	pSerOTSλ	Kanamycin, 37 ˚C	(Pirman et al, 2015)
G30	supD tRNA^{Ser} Suppressor	Kanamycin, 37 ˚C	(Pirman et al, 2015)
	pSerOTSc		
Q73	ColE1, proK-1x tRNA, glnS*-pSerRS9-EF-pSer21	Kanamycin, 37 ˚C	This Study
	pSerOTSc - tRNA Variants		
P73	ColE1, proK-2x tRNA	Kanamycin, 37 ˚C	This Study
P74	ColE1, proK-4x tRNA	Kanamycin, 37 ˚C	This Study
P75	ColE1, proK-6x tRNA	Kanamycin, 37 ˚C	This Study
	pSerOTSc - ColE1+ Rop tRNA Variants		
R20	ColE1 + Rop, proK-1x tRNA	Kanamycin, 37 ˚C	This Study
Q58	ColE1 + Rop, proK-2x tRNA	Kanamycin, 37 ˚C	This Study
Q59	ColE1 + Rop, proK-4x tRNA	Kanamycin, 37 ˚C	This Study
Q60	ColE1 + Rop, proK-6x tRNA	Kanamycin, 37 ˚C	This Study
	pSerOTSc - p15a tRNA Variants		
R19	p15a, proK-1x tRNA	Kanamycin, 37 ˚C	This Study
P5	p15a, proK-2x tRNA	Kanamycin, 37 ˚C	This Study
P32	p15a, proK-4x tRNA	Kanamycin, 37 ˚C	This Study
P33	p15a, proK-6x tRNA	Kanamycin, 37 ˚C	This Study
	pSerOTSc - Modified tRNA		
V70	ColE1, G2:C71 1x tRNA	Kanamycin, 37 ˚C	This Study
	OTS Components		
R4	pSerOTSλ TRC*-pSerRS only	Kanamycin, 37 ˚C	This Study
Q81	pSerOTSc glnS*-pSerRS only	Kanamycin, 37 ˚C	This Study
R9	pSerOTSλ lpp-5x tRNA only	Kanamycin, 37 ˚C	This Study
V60	ColE1, G2:C71 1x tRNA only	Kanamycin, 37 ˚C	This Study
R11	ColE1, proK-2x tRNA only	Kanamycin, 37 ˚C	This Study
R12	ColE1, proK-4x tRNA only	Kanamycin, 37 ˚C	This Study
R3	ColE1, proK-6x tRNA only	Kanamycin, 37 ˚C	This Study
	Recombinant Reporter Expression		
C9	E(17)TAG-GFP, aTc Inducible, RSF1030	Chloramphenicol, 37˚C	(Pirman et al, 2015)
E52	TAG-MS-READ mass spectrometry reporter, aTc Inducible, p15a	Ampicillin, 37 ˚C	This Study

aTc = anhydrotetracycline IPTG= Isopropyl β- d-1-thiogalactopyranoside Ara= Arabinose

Supplemental References

Lajoie MJ, Rovner AJ, Goodman DB, Aerni HR, Haimovich AD, Kuznetsov G, Mercer JA, Wang HH, Carr PA, Mosberg JA et al (2013) Genomically recoded organisms expand biological functions. *Science* **342:** 357-360

Mukai T, Hoshi H, Ohtake K, Takahashi M, Yamaguchi A, Hayashi A, Yokoyama S, Sakamoto K (2015) Highly reproductive Escherichia coli cells with no specific assignment to the UAG codon. *Scientific reports* **5:** 9699

Pirman NL, Barber KW, Aerni HR, Ma NJ, Haimovich AD, Rogulina S, Isaacs FJ, Rinehart J (2015) A flexible codon in genomically recoded Escherichia coli permits programmable protein phosphorylation. *Nature communications* **6:** 8130

Steinfeld JB, Aerni HR, Rogulina S, Liu Y, Rinehart J (2014) Expanded cellular amino acid pools containing phosphoserine, phosphothreonine, and phosphotyrosine. *ACS Chem Biol* **9:** 1104-1112

Studier FW, Moffatt BA (1986) Use of bacteriophage T7 RNA polymerase to direct selective high-level expression of cloned genes. *J Mol Biol* **189:** 113-130

Zhu P, Gafken PR, Mehl RA, Cooley RB (2019) A Highly Versatile Expression System for the Production of Multiply Phosphorylated Proteins. *ACS chemical biology* **14:** 1564-1572
