# Supplementary material for: System‐wide optimization of an orthogonal translation system with enhanced biological tolerance
Source: Mol Syst Biol. 2023 Jul 21;19(8):e10591. doi: 10.15252/msb.202110591 (PMC10407733; doi:10.15252/msb.202110591)
Supplement: Supplementary file 6 [file MSB-19-e10591-s005.pdf]

System-wide Optimization of an Orthogonal Translation System with Enhanced Biological Tolerance

Kyle Mohler, Jack Moen, Svetlana Rogulina, and Jesse Rinehart
DOI: 10.15252/msb.202110591

Corresponding author(s): Jesse Rinehart (jesse.rinehart@yale.edu)

Review Timeline:

Submission Date:	20th Jul 21
Editorial Decision:	17th Aug 21
Revision Received:	10th May 23
Editorial Decision:	5th Jun 23
Revision Received:	30th Jun 23
Editorial Decision:	3rd Jul 23
Revision Received:	5th Jul 23
Accepted:	5th Jul 23

Editor: Maria Polychronidou

Transaction Report:

(Note: With the exception of the correction of typographical or spelling errors that could be a source of ambiguity, letters and reports are not edited. Depending on transfer agreements, referee reports obtained elsewhere may or may not be included in this compilation. Referee reports are anonymous unless the Referee chooses to sign their reports.)

17th Aug 2021

RE: MSB-2021-10591, System-wide Optimization of an Orthogonal Translation System with Enhanced Biological Tolerance

Dear Prof. Rinehart,

Thank you again for submitting your work to Molecular Systems Biology. We have now heard back from the three referees who agreed to evaluate your study. Overall, the reviewers acknowledge that the study is a relevant contribution to the field. They raise however a series of concerns, which we would ask you to address in a revision.

I think that the reviewers' recommendations are clear and therefore it is not required to repeat the points listed below. All issues raised by the reviewers need to be satisfactorily addressed. Please contact me in case you would like to discuss in further detail any of the issues raised.

On a more editorial level, we would ask you to address the following points:

- Please provide a .doc file for the manuscript text (including legends for the main figures) and individual production-quality files for the main figures (one file per figure).

- The keywords need to be reduced to 5.

- Please include an 'Author Contributions' and a 'Conflict of Interest statement' in the main text.

- We have replaced Supplementary Information by the Expanded View (EV format). In this case, all additional figures and Tables can be included in a PDF called Appendix. Appendix figures and Tables should be labeled and called out as: "Appendix Figure S1, Appendix Figure S2... Appendix Table S1..." etc. Each legend should be below the corresponding Figure/Table in the Appendix. Please include a Table of Contents in the beginning of the Appendix. For detailed instructions regarding expanded view please refer to our Author Guidelines: .

- Please provide a "standfirst text" summarizing the study in one or two sentences (approximately 250 characters), three to four "bullet points" highlighting the main findings and a "synopsis image" (550px width and max 400px height, jpeg format) to highlight the paper on our homepage.

- All Materials and Methods need to be described in the main text. We would encourage you to use 'Structured Methods', our new Materials and Methods format. According to this format, the Materials and Methods section should include a Reagents and Tools Table (listing key reagents, experimental models, software and relevant equipment and including their sources and relevant identifiers) followed by a Methods and Protocols section in which we encourage the authors to describe their methods using a step-by-step protocol format with bullet points, to facilitate the adoption of the methodologies across labs. More information on how to adhere to this format as well as downloadable templates (.doc or .xls) for the Reagents and Tools Table can be found in our author guidelines:
. An example of a Method paper with Structured Methods can be found here:

- For data quantification: please specify the name of the statistical test used to generate error bars and P values, the number (n) of independent experiments (specify technical or biological replicates) underlying each data point and the test used to calculate p-values in each figure legend. The figure legends should contain a basic description of n, P and the test applied. Graphs must include a description of the bars and the error bars (s.d., s.e.m.).

- The References need to be formatted according to the Molecular Systems Biology reference style.

- When you resubmit your manuscript, please download our CHECKLIST (https://bit.ly/EMBOPressAuthorChecklist) and include the completed form in your submission.
Please note that the Author Checklist will be published alongside the paper as part of the transparent process (https://www.embopress.org/page/journal/17444292/authorguide#transparentprocess).

If you feel you can satisfactorily deal with these points and those listed by the referees, you may wish to submit a revised version of your manuscript. Please attach a covering letter giving details of the way in which you have handled each of the points raised by the referees. A revised manuscript will be once again subject to review and you probably understand that we can give you no guarantee at this stage that the eventual outcome will be favorable.

Kind regards,

Maria

Maria Polychronidou, PhD
Senior Editor
Molecular Systems Biology

If you do choose to resubmit, please click on the link below to submit the revision online *within 90 days*.

https://msb.msubmit.net/cgi-bin/main.plex

IMPORTANT: When you send your revision, we will require the following items:
1. the manuscript text in LaTeX, RTF or MS Word format
2. a letter with a detailed description of the changes made in response to the referees. Please specify clearly the exact places in the text (pages and paragraphs) where each change has been made in response to each specific comment given
3. three to four 'bullet points' highlighting the main findings of your study
4. a short 'blurb' text summarizing in two sentences the study (max. 250 characters)
5. a 'thumbnail image' (550px width and max 400px height, Illustrator, PowerPoint or jpeg format), which can be used as 'visual title' for the synopsis section of your paper.
6. Please include an author contributions statement after the Acknowledgements section (see https://www.embopress.org/page/journal/17444292/authorguide)
7. Please complete the CHECKLIST available at (https://bit.ly/EMBOPressAuthorChecklist).
Please note that the Author Checklist will be published alongside the paper as part of the transparent process (https://www.embopress.org/page/journal/17444292/authorguide#transparentprocess).
8. Please note that corresponding authors are required to supply an ORCID ID for their name upon submission of a revised manuscript (EMBO Press signed a joint statement to encourage ORCID adoption).
(https://www.embopress.org/page/journal/17444292/authorguide#editorialprocess)

Currently, our records indicate that the ORCID for your account is 0000-0003-4839-4005.

Please click the link below to modify this ORCID:
Link Not Available

The system will prompt you to fill in your funding and payment information. This will allow Wiley to send you a quote for the article processing charge (APC) in case of acceptance. This quote takes into account any reduction or fee waivers that you may be eligible for. Authors do not need to pay any fees before their manuscript is accepted and transferred to the publisher.

As a matter of course, please make sure that you have correctly followed the instructions for authors as given on the submission website.

*** PLEASE NOTE *** As part of the EMBO Press transparent editorial process initiative (see our Editorial at https://dx.doi.org/10.1038/msb.2010.72), Molecular Systems Biology publishes online a Review Process File with each accepted manuscripts. This file will be published in conjunction with your paper and will include the anonymous referee reports, your point-by-point response and all pertinent correspondence relating to the manuscript. If you do NOT want this File to be published, please inform the editorial office at msb@embo.org within 14 days upon receipt of the present letter.

Reviewer #1:

This manuscript by Mohler et al systematically explores how orthogonal translation systems (OTSs) and their components interact globally with cellular components. Making use of previously-employed and novel pSerOTSs, the authors use a wide variety of biochemical assays and techniques to thoroughly characterize detrimental effects the introduction of an OTS into (recoded) E. coli can have. Negative effects on cellular growth and viability are shown to relate to stress responses that vary with the concentration of aaRS and tRNAs present. The notion that the orthogonality of an OTS is a dynamic, rather than static condition, is well attested. Moreover, the authors provide solutions for engineering tRNAs or manipulating concentrations of OTS components to enable the efficient incorporation of pSer into a variety of model proteins.

As someone, who has first-hand experience of the detrimental effects the introduction of OTSs in host organisms can cause, this reviewer read this manuscript with great interest. While it was long suspected/known that undesired interactions between OTS-components and the cell are responsible for growth defects, this study provides key insight on how to characterize and prevent these interactions. As such, this reviewer believes that the workflow (proteomics, plasmids with different ORIs, OTSs,

tRNA-copy numbers, UAG-MS-READ reporters, etc.) introduced in this manuscript will greatly benefit the engineering of more efficient OTSs in the future.

Overall, this is a highly interesting, novel, and well-executed study and I therefore recommend the publication of the manuscript in Molecular Systems Biology.

Reviewer #2:

Summary

This manuscript describes the characterization and optimization of an orthogonal translation system (OTS) in E. coli. The authors found that varying the expression levels and/or copy numbers of orthogonal aaRS enzymes and tRNAs affects protein expression yields and cell growth rates. Based on proteomic analyses, the authors suggest that growth effects may arise from perturbations to host protein levels involved in translation and amino acid biosynthesis. The authors further demonstrate that overexpression of an orthogonal aaRS enzyme leads to proteome-wide misincorporation of the unnatural pSer amino acid. The authors also observe misincorporation of natural amino acids at the pSer codon of the target recombinant protein, and suggest that host aaRS enzymes are at least partially responsible. Based on these findings, the authors improved recombinant protein yield by adjusting OTS component expression levels and introducing a mutation in the orthogonal tRNA to minimize interactions with host aaRS enzymes. The authors suggest that future attempts to successfully implement OTSs should apply similar analytical approaches to probe interactions between OTSs and host proteins.

Overall, the data and the approach reported in this manuscript will be useful to a broad audience interested in using OTSs for unnatural amino acid incorporation. However, the manuscript itself was extremely difficult to follow and needs extensive editing before publication. The authors are strongly encouraged to carefully review the manuscript, paragraph by paragraph, to address confusing text, figures, and overall logic. Also, some critical experimental comparisons are unclear and may not be sufficient to support the broad claims made by the authors. I think that there is data to support the general claims described in the summary above, and the authors can revise the manuscript without major new experiments.

Major comments

1. The structure of many figures is confusing, which severely impedes the readability of the manuscript. For example, in Fig 1, there should be labels for each construct in Fig 1B that match descriptions in the text and in other panels of the figure. In Fig 1C, why are there two distinct horizontal axis labels (top black text, bottom blue text)? Which labels correspond to which row? Is there a missing black label for the final column? Why does "Lambda" appear twice on the right side and what does it mean? What is the scale of the green shading? Do the arrows to panels D and E indicate which row matches the data in the bar graphs? I can take a reasonable guess for each of these questions but I shouldn't have to. Similar issues recur in other figures throughout the manuscript.

2. The text of the manuscript is confusing. For example, page 6 is very difficult to parse due to jargon and labels that are not clearly explained. This problem is compounded by the confusing structure of Fig 1. As a specific example, on line 166 the authors write: "Both rEcoliXpS and BL21 (DE3) cells harboring OTS variants with p15a origins were viable and generally tolerated increases in tRNA copy number (Figure 1C-D)." This sentence implies that cells with p15a origins are shown in Fig 1D, but the arrow in the figure itself implies that Fig 1D shows cells with ColE1 origins. What cells are being shown in Fig 1D? A small inconsistency like this would normally be a minor comment, but similar issues recur throughout this section and the rest of the manuscript.

3. The manuscript includes many sentences that are vague. For example, on pg 17, line 349, the authors write: "To better understand the implications of orthogonal tRNA introduction to the host cell, we thoroughly characterized the interactions between tRNApSer and host physiology with particular emphasis on translational fidelity." What exactly does "implications" mean? Especially by this point in the manuscript, the authors should be specific about what they are looking at. What does it mean that they "thoroughly characterized the interactions between tRNApSer and host physiology"? Such a characterization could inclede an extremely broad range of assays, but the authors then refer specifically to translational fidelity. The authors should precisely and concisely describe what questions they are addressing and how they will address these questions. Similar vague statements recur throughout the manuscript.

4. The logic of several sections of the manuscript is confusing. For example on pg 19, line 410, the authors write: "While de-phosphorylation of pSer may contribute to the Ser levels, the tRNA-dependent increase in the relative ratio of Ser:pSer points towards a specific interaction between pSerRS and tRNApSer which results in Ser misaminoacylation." What exactly do the authors mean by "a specific interaction"? Written this way, the authors are implying that the increase in the relative ratio is positive evidence for a model where pSerRS is responsible for misacylation. At best, this claim is not obvious (see below, points 5-8). Similar confusing logic recurs throughout the manuscript.

5. To address the confusing logic noted above, the authors should explicitly describe models and corresponding predictions. The authors consider three alternative models to explain how Ser is misincorporated at the pSer codon, although the authors do not clearly describe these models in the text. My attempt to reconstruct the logic follows. Model 1: pSer is dephosphorylated after aminoacylation, either in the aminoacylated tRNA or the translated protein. Model 2: Native SerRS misacylates tRNApSer with unphosphorylated Ser. Model 3: pSerRS can use unphosphorylated Ser as a substrate to acylate tRNApSer. The authors suggest that model 1 is unlikely because increasing tRNA levels should not increase the Ser:pSer incorporation ratio (lines 410-411). The authors do not clearly explain the logic behind this assertion, and multiple possibilities need to be considered. If dephosphorylation occurs after translation, then the Ser:pSer ratio should be unaffected by tRNA levels. If dephosphorylation occurs on the aminoacylated tRNA, then the effect on the Ser:pSer ratio depends on whether the phosphatase is saturated or not. If the phosphatase is saturated, the Ser:pSer ratio should decrease as the tRNA level increases because there will be more aminoacyl-pSer but the dephosphorylation rate does not change. If the phosphatase is not saturated, the Ser:pSer ratio will stay the same because the phosphatase will produce the same fraction of aminoacyl-Ser to aminoacyl-pSer. The observed Ser:pSer ratio does increase with increasing tRNA (Fig 4C), so the authors rule out model 1. It is critical that the authors supply a clear explanation of the logic, which appears to be much more complicated than implied by the brief description on lines 410-411.

6. Model 2 is that native SerRS misacylates tRNApSer with unphosphorylated Ser. As the authors note earlier with respect to Gly/Thr misincorporation (line 398), increasing tRNA levels could plausibly lead to higher levels of misacylation by a host aaRS enzyme. However, the authors note that "Ser misincorporation required both orthogonal aaRS and tRNA expression and was absent in the isolation of either component" (line 402), suggesting that host aaRS enzymes are not responsible for Ser misincorporation. The authors also note that based on limited homology between tRNASer and tRNApSer, the host SerRS enzyme is unlikely to act on tRNApSer. At this point in the manuscript, this logic seems plausible, leading the authors to conclude that Model 3, the only remaining model, is correct. However, Model 3 does not appear to be consistent with the data (see below).

7. Model 3 is that pSerRS can use unphosphorylated Ser as a substrate to acylate tRNApSer. The authors should make this statement clearly. They instead use vague language about "a specific interaction between pSerRS and tRNApSer which results in Ser misaminoacylation" (lines 411-412). More importantly, it is not immediately obvious why this model predicts that increases in tRNA levels should increase the Ser:pSer ratio. My expectation is that the Ser:pSer ratio would not be affected by increasing tRNA levels because pSerRS will most likely produce the same ratio of Ser misacylation to correct pSer acylation regardless of tRNA concentration. Can the authors clarify this point? It is certainly possible that my logic is incorrect here, but the manuscript lacks a clear explanation that would allow the reader to evaluate this issue.

8. Data presented later in the manuscript is more consistent with Model 2 than Model 3. On line 440 the authors note that mutations to tRNA-pSer result in a decrease in Ser misincorporation. They write: "At the same time, a large decrease in Ser:pSer relative incorporation rates were also observed, suggesting that the sequence modification made to the orthogonal tRNA may have reduced Ser misaminoacylation (Figure 4E)." As noted by the authors in the context of Gly/Thr misincorporation (lines 428-434), this observation is most consistent with misincorporation due to host aaRS action (Model 2) because mutations in the tRNA could perturb misacylation by a host aaRS without necessarily affecting pSer incorporation by pSerRS. It is not obvious why the Ser:pSer ratio would decrease if Model 3 were correct. Once again, multiple possibilities need to be considered. If pSerRS residues involved in tRNA binding are not thermodynamically coupled to active site residues involved in catalysis, then the simplest expectation is that mutations in the tRNA will have the same effect on acylation with Ser or pSer, leaving the Ser:pSer ratio unchanged. Alternatively, it is possible that tRNA binding and catalysis are thermodynamically linked, so that mutations to the tRNA could have different effects on Ser vs. pSer acylation. Such effects could increase or decrease the Ser:pSer ratio, and it is not clear that there is a strong expectation either way. At minimum the authors must explicitly lay out the alternative models and corresponding expectations.

9. Statements about the yield of recombinant pSer-containing protein are central to the manuscript, but the key comparison in the gel in Fig 1D is unclear. What exactly was loaded on the gel? Was it fixed amounts of cells? Was it equal volumes from each culture with different amounts of cells depending on the growth rate? Was it a clarified lysate normalized to the same amount of total protein in each well? This information will indicate whether we are considering yield in the context of mg protein per liter of culture or fraction of protein relative to total protein. If gel samples were a fixed volume of culture, should the reader care about the decrease in growth rate from 1x to 4x tRNA if the absolute protein yield is apparently increasing?

10. The importance and significance of Fig 5 is unclear. Was the optimized tRNApSer from Fig 4E used in the experiments in Fig 5? The text is vague but it seems like the optimized guide was not used. If so, Fig 5 and the corresponding sections seem to reiterate findings about expression levels that were detailed in prior sections. I encourage the authors to be as clear as possible about which tRNA is being used in Fig 5, and to consider restructuring the manuscript to place these experiments before the data shown in Fig 4.

Minor comments

1. On line 88, the authors write: "Although the study of the pSerOTS itself has been significant..." It is not clear what the authors mean by "significant".

2. On line 163, the authors refer to the Rop protein. The authors should explain what Rop is and how it affects plasmid copy number. No citation is provided for a general reader to understand what Rop is or what it is expected to do.

3. On line 175, the authors write: "In comparison to E. coli lacking an OTS (WT), cells expressing glnS*-pSerRS showed a slight growth defect, while cells expressing TRC*-pSerRS displayed a significant decrease in growth (Figure 1E)." The text describes a comparison to WT but Figure 1E does not show the WT growth rate. It is possible to make comparisons to WT in panel 1C, but the comparison is based on subtle differences in green shading that are not easy to evaluate.

4. On lines 178-183, the authors refer to comparisons between growth rates shown in Fig 1E that are within or barely outside the error bars. Can the authors clarify what constitutes a significant change in these values?

5. On line 192, the authors write "Interestingly, the tRNA copy number dependent decrease in cellular viability was inversely proportional to the amount of non-pSer containing GFP reporter (Figure 1D). This observation suggests that tRNApSer misaminoacylation may play a role observed decrease in viability." Although the tRNApSer misaminoacylation idea is corroborated later in the manuscript, at this point the logical connection is not obvious. Can the authors rule out an alternative model where high tRNA levels cause burdens on host systems that perturb cell growth? An appropriate control would be to overexpress some unrelated tRNA to see how it affects pSer incorporation.

6. Similar questions arise for the effect of pSerRS levels on cell growth (Fig 1D/E). Are these effects specific to pSerRS or would they occur when any generic protein was expressed to a similar level? Do the authors have any growth measurements with generic protein overexpression controls for comparison to the pSerRS strains?

7. On line 224, the authors write: "the abundance of pSerRS is likely to challenge the cell by disrupting the binding equilibrium within amino acid and tRNA substrate pools." It is not clear what "equilibrium" means in this context. Ref 39 cited by the authors does a reasonable job of using different language to make the same point more clearly.

8. What are the units of the x-axis in Fig 1E? Is it normalized to fold-change?

9. On line 284, the authors write: "Overall, these data reinforce the notion that fine-tuning OTS component expression based on in-depth proteomic analysis can minimize the impact of an OTS on the host cell." The major comparisons are between just two systems, pSerOTSc and pSerOTSlambda. These differences are meaningful and useful to delineate, but it seems like a stretch to call a comparison between two systems "fine-tuning".

10. Figure 3A is difficult to follow. (1) It would be helpful to include entries for putative pSer peptides in the second and third sets, even if they are below the limit of detection. (2) The alignment of sequences is confusing. The sequences inside the grey balls are barely readable without zooming far in, and it would help if those residues (and particularly the green ball) were lined up with the corresponding sequences below. Alternatively, the authors could remove the grey balls and simply highlight the residue of interest within each text string.

11. In the MS-READ assay shown in Fig 3A, does pS incorporation occur at other Gly codons in the peptide? Can the authors explain briefly how MS-READ identifies misincorporation at a specific site? This manuscript refers to ref 48, which does not clearly address this question and refers to additional references in its own methods section.

12. On line 323, the authors write: "Without phospho-enrichment strategies, we found a total of 33 unique peptides..." How should this number be evaluated? Is 33 higher or lower than one might expect? The Fig 3C legend indicates that proteomes from pSerOTSlambda and lambda aaRS cells were search for misincorporation. The manuscript text is unclear about which cells were the source of the 33 peptides.

13. It is not clear how to interpret Fig 3C and the figure legend does not provide sufficient explanation. What are the red lines and how do they tell us about pSer incorporation?

14. On line 325, the authors write: "The misincorporation of pSer at Gly codons is an important example of native proteome damage caused by an orthogonal aaRS and highlights importance of monitoring and tuning aaRS expression during OTS development." If the goal of OTSs is to produce proteins of interest with ncAA incorporation, and that goal can be achieved, why does it matter if there is misincorporation in the host genome? Do the authors expect that pSer misincorporation reduces yield of desired recombinant protein? Are there specific cases where incorporation of ncAAs has failed or produced low yield that can attributed to misincorporation in the host genome?

15. On line 344, the authors write: "We noticed that increasing the copy number of the suppressor tRNA while decreasing the levels of pSerRS led to an increase in non-phospho recombinant protein (Figure 1D)." It is difficult to evaluate this statement based on a gel image. Can the gel band intensities be quantified?

16. In Fig 4C, middle panel, the "ThrRS/pSerRS" label for tRNA identity elements should be "SerRS/pSerRS".

17. On line 442, the authors write: "A Phos-tagTM assay reflected the increase in recombinant protein purity quantified in the MS experiment (Figure 4E)." Fig 4E shows only a single gel lane for the optimized system. If the point is to show an increase, it would be helpful to show this sample side-by-side on the same gel with at least one of the original OTS systems. As noted above, it would also be helpful to explain what exactly is being loaded on the gel so it is clear whether the increase is relative to culture volume or total protein.

18. The wording on lines 464-472 is confusing and it is difficult to parse which cell type is used with each vector

19. On lines 514-516, the authors write: "The 6x tRNA OTS, for example, is robustly expressed (Lane 6, lower), but the proportion of phospho-mTOR production (Lane 6, upper) is significantly lower when compared to the 1x tRNA OTS counterpart (Lane1) (Figure 5E)." Is "robustly expressed" referring to mTOR or the OTS components? The text makes it seem that the OTS components have robust expression, but Fig 5E indicates that this point is in reference to mTOR.

20. On line 515-516, the authors refer to Fig 5E, lane 6 but there is no lane 6 in Figure 5E. Do the authors mean lane 5?

21. On line 582, the authors write: "Traditional methods of analyzing tRNA orthogonality fail to address this dynamic property of tRNA recognition and are therefore more susceptible to reductions in OTS fidelity due to cellular perturbation." What does this sentence mean? Most simply, there is a subject-verb problem here. The subject of the sentence appears to be "traditional methods of analyzing tRNA orthogonality". How does it make sense to say that traditional analysis methods more susceptible to reductions in fidelity? Again, issues like this are typically minor but occur so frequently in this manuscript that they severely impact its readability.

22. Typos: line 48 "is often" should be "often", line 76 & 196 "it's" should be "its", missing words in the sentence on lines 194-195.

Reviewer #3:

Summary:
Mohler et al describe a thorough characterization of how an orthogonal translation system (OTS), specifically the pSerRS and tRNApSer system, can affect the viability, proteome, and tRNA pool of E. coli and use their results to improve pSer incorporation through amber suppression. First, they analyze how the expression of pSerRS and tRNApSer in two E. coli strain backgrounds, rEcoli and BL21, affect cell viability and protein expression. Strong overexpression of either OTS component was observed to reduce growth rate and accurate pSer incorporation into proteins, and that the growth rate reduction was more significant in BL21. By analyzing the proteome of rEcoli containing these OTS components, they find that cells where pSerRS is overexpressed upregulate translation-associated proteins, suggesting increased cellular burden. Lower expression of pSerRS from the weaker glnS promoter were not observed to similarly upregulate translation-associated proteins. Next, the authors analyzed the orthogonality of the pSer OTS towards native E. coli aaRSs and tRNAs using a mass-spectrometry based assay, MS-READ. They find that pSerRS misacylates native tRNAs, such as tRNAGly, that native aaRSs misacylate tRNApSer with Gly and Thr, and that pSerRS misacylates tRNApSer with Ser. The authors were able to successfully decrease Gly and Thr misacylation by engineering tRNA identity elements and observed reduced Ser misacylation as well. Finally, the authors optimize pSer incorporation into proteins of interest using their best OTS constructs, resulting in improved yields of phosphoproteins as analyzed by gel.

General remarks:
A systems-level understanding of how orthogonal translation systems interact with native E. coli translation components is of interest to the genetic code expansion community, as it would enable more deliberate engineering of orthogonal translation systems. The key data presented in this work are the characterizations that the authors used to understand these interactions. In particular are the proteomics of total E. coli protein of rEcoli, which provides data on how it must redirect resources towards producing the OTS and the protein of interest, and MS-READ, which is a mass-spectrometry based technique previously developed by the authors to decipher interactions between aaRSs, tRNAs, and amino acids. The advances made in this work result in an OTS that appears to have less cellular burden and higher specificity for pSer incorporation.

However, the proteomics data does not appear to have informed the design of orthogonal translation systems, as it appears to have followed the optimization in Figure 1, and it does not seem to provide novel conclusions, as most of the effects are related to genes linked to protein overexpression, which seems intuitive. This was disappointing. With further analysis, described below, I believe this could be a useful data set for the genetic code expansion community more broadly.

The experiments surrounding tRNA engineering using MS-READ thoroughly analyze the fidelity of their OTS. A follow up experiment, described below, should be conducted to characterize the amino acid specificity of pSerRS. Generally, however, I believe this section is well done and describes a strong method to analyze pSer incorporation fidelity.

Major points:
It is unclear if the proteomics data, particularly for constructs expressing pSerRS, is specific to pSerRS and thus provides insight for engineering organisms with expanded genetic codes, or is indicative of more general cellular consequences due to protein overexpression. A useful experiment would be to compare the proteomics data with data from a rEcoli strain that contains similar constructs as pSerOTSc and Lambda but expresses a "dummy" protein that does not interact with native translation machinery. This would clarify if these proteome changes are indicative of unique interactions between the pSerOTS and native translation machinery rather than the cellular burden of protein overexpression. Put another way, is this a systems effect?

The section between lines 343 -392 would benefit from a further experiment on the specificity of the pSerRS. One possibility for misincorporation that is not addressed in the current text is the polyspecificity of engineered aaRSs. This could be tested by challenging rEcoli containing pSerRS and its cognate tRNA to make a reporter in the absence of Sep and could be analyzed using MS-READ. Any reporter protein synthesized that is not found in the data analyzing near-cognate UAG suppression would then likely be a result of pSerRS polyspecificity towards other amino acids. This would further support conclusions that native aaRSs are misacylating tRNApSer by showing the specificity of pSerRS.

It is unclear why the tRNAOptpSer and pSerOTSc* that was engineered in Figure 4 did not appear to be used in the final experiments in Figure 5. A discussion clarifying this decision would be appreciated. Even better, the experiments in Figure 5 could be re-done if it will impact the story.

Please include phosphoprotein yields (g/L), as this is a useful metric that would help guide the choice of OTS for a particular experiment, and help others assess the usefulness of the work for those that want to make phosphoproteins.

Minor points
- The source of Sep in these experiments is unclear. Please clarify if Sep is biosynthesized in vivo or if exogenous Sep was supplemented into growth medium.
- Names for appropriate strains should be clearly defined early in the text. For example, "Lambda" is used in Figure 1C but is not defined until Line 234.
- Figure 1C could also be improved by clearly defining each condition. For example, the first column in Figure 1C is described both as "No OTS" and "glnS*-aaRS," which is confusing.
- A color bar on Figure 1C and a label clearly showing that numbers Figure 2A represent rank would improve readability.
- The data in Supplementary Figures 3 and 4 would be better served by a table containing the proteins that were significantly up/downregulated.
- The sentence starting on line 469 ("Lanes 2 and 3...") should reference a figure.

Point-by-Point Response to Reviewer Comments
Our responses in Blue
Original reviewer comments in black

Reviewer #1:

We would like to thank Reviewer #1 for taking the time to review this manuscript and we greatly appreciate the positive feedback. We hope this reviewer will find the extensively revised manuscript equally as suitable for publication.

This manuscript by Mohler et al systematically explores how orthogonal translation systems (OTSs) and their components interact globally with cellular components. Making use of previously-employed and novel pSerOTSs, the authors use a wide variety of biochemical assays and techniques to thoroughly characterize detrimental effects the introduction of an OTS into (recoded) E. coli can have. Negative effects on cellular growth and viability are shown to relate to stress responses that vary with the concentration of aaRS and tRNAs present. The notion that the orthogonality of an OTS is a dynamic, rather than static condition, is well attested. Moreover, the authors provide solutions for engineering tRNAs or manipulating concentrations of OTS components to enable the efficient incorporation of pSer into a variety of model proteins.

As someone, who has first-hand experience of the detrimental effects the introduction of OTSs in host organisms can cause, this reviewer read this manuscript with great interest. While it was long suspected/known that undesired interactions between OTS-components and the cell are responsible for growth defects, this study provides key insight on how to characterize and prevent these interactions. As such, this reviewer believes that the workflow (proteomics, plasmids with different ORIs, OTSs, tRNA-copy numbers, UAG-MS-READ reporters, etc.) introduced in this manuscript will greatly benefit the engineering of more efficient OTSs in the future.

Overall, this is a highly interesting, novel, and well-executed study and I therefore recommend the publication of the manuscript in Molecular Systems Biology.

Reviewer #2:

Summary

This manuscript describes the characterization and optimization of an orthogonal translation system (OTS) in E. coli. The authors found that varying the expression levels and/or copy numbers of orthogonal aaRS enzymes and tRNAs affects protein expression yields and cell growth rates. Based on proteomic analyses, the authors suggest that growth effects may arise from perturbations to host protein levels involved in translation and amino acid biosynthesis. The authors further demonstrate that overexpression of an orthogonal aaRS enzyme leads to proteome-wide misincorporation of the unnatural pSer amino acid. The authors also observe misincorporation of natural amino acids at the pSer codon of the target recombinant protein, and suggest that host aaRS enzymes are at least partially responsible. Based on these findings, the authors improved recombinant protein yield by adjusting OTS component expression levels and introducing a mutation in the orthogonal tRNA to minimize interactions with host aaRS enzymes. The authors suggest that future attempts to successfully implement OTSs should apply similar

analytical approaches to probe interactions between OTSs and host proteins.

Overall, the data and the approach reported in this manuscript will be useful to a broad audience interested in using OTSs for unnatural amino acid incorporation. However, the manuscript itself was extremely difficult to follow and needs extensive editing before publication. The authors are strongly encouraged to carefully review the manuscript, paragraph by paragraph, to address confusing text, figures, and overall logic. Also, some critical experimental comparisons are unclear and may not be sufficient to support the broad claims made by the authors. I think that there is data to support the general claims described in the summary above, and the authors can revise the manuscript without major new experiments.

Major comments

We would like to thank Reviewer # 2 for providing such an extensive commentary. It is clear that they read the manuscript thoroughly and we appreciate that effort and corresponding feedback. To address the concerns below, we have re-made every figure, keeping in mind the concerns discussed below, in addition to an extensive re-write of the manuscript text to reduce jargon and enhance clarity and readability for a more general audience.

1. The structure of many figures is confusing, which severely impedes the readability of the manuscript. For example, in Fig 1, there should be labels for each construct in Fig 1B that match descriptions in the text and in other panels of the figure. In Fig 1C, why are there two distinct horizontal axis labels (top black text, bottom blue text)? Which labels correspond to which row? Is there a missing black label for the final column? Why does "Lambda" appear twice on the right side and what does it mean? What is the scale of the green shading? Do the arrows to panels D and E indicate which row matches the data in the bar graphs? I can take a reasonable guess for each of these questions but I shouldn't have to. Similar issues recur in other figures throughout the manuscript.

Each figure has been substantially revised to improve the clarity, structure, and consistency.

2. The text of the manuscript is confusing. For example, page 6 is very difficult to parse due to jargon and labels that are not clearly explained. This problem is compounded by the confusing structure of Fig 1. As a specific example, on line 166 the authors write: "Both rEcoliXpS and BL21 (DE3) cells harboring OTS variants with p15a origins were viable and generally tolerated increases in tRNA copy number (Figure 1C-D)." This sentence implies that cells with p15a origins are shown in Fig 1D, but the arrow in the figure itself implies that Fig 1D shows cells with ColE1 origins. What cells are being shown in Fig 1D? A small inconsistency like this would normally be a minor comment, but similar issues recur throughout this section and the rest of the manuscript.

Figure 1 has been substantially revised to improve clarity and the associated text has been revised to reduce jargon and enhance accessibility.

3. The manuscript includes many sentences that are vague. For example, on pg 17, line 349, the authors write: "To better understand the implications of orthogonal tRNA introduction to the host cell, we thoroughly characterized the interactions between tRNApSer and host physiology with particular emphasis on translational fidelity." What exactly does "implications" mean? Especially by this point in the manuscript, the authors should be specific about what they are

looking at. What does it mean that they "thoroughly characterized the interactions between tRNApSer and host physiology"? Such a characterization could include an extremely broad range of assays, but the authors then refer specifically to translational fidelity. The authors should precisely and concisely describe what questions they are addressing and how they will address these questions. Similar vague statements recur throughout the manuscript.

Overall, the manuscript text has been extensively revised to reduce vague statements and replace them with clearly stated objectives. This specific comment was addressed through text revision.

4. The logic of several sections of the manuscript is confusing. For example on pg 19, line 410, the authors write: "While de-phosphorylation of pSer may contribute to the Ser levels, the tRNA-dependent increase in the relative ratio of Ser:pSer points towards a specific interaction between pSerRS and tRNApSer which results in Ser misaminoacylation." What exactly do the authors mean by "a specific interaction"? Written this way, the authors are implying that the increase in the relative ratio is positive evidence for a model where pSerRS is responsible for misacylation. At best, this claim is not obvious (see below, points 5-8). Similar confusing logic recurs throughout the manuscript.

Substantial effort has been made to clearly present the logic and motivations for the study by remaking figures and rearranging figure data into a more logical order. Additionally, models of OTS toxicity have been added to both the figures and the manuscript text to clearly define the opportunities for OTS mediated reductions in translational fidelity, specifically mentioned in this comment.

5. To address the confusing logic noted above, the authors should explicitly describe models and corresponding predictions. The authors consider three alternative models to explain how Ser is misincorporated at the pSer codon, although the authors do not clearly describe these models in the text. My attempt to reconstruct the logic follows. Model 1: pSer is dephosphorylated after aminoacylation, either in the aminoacylated tRNA or the translated protein. Model 2: Native SerRS misacylates tRNApSer with unphosphorylated Ser. Model 3: pSerRS can use unphosphorylated Ser as a substrate to acylate tRNApSer. The authors suggest that model 1 is unlikely because increasing tRNA levels should not increase the Ser:pSer incorporation ratio (lines 410-411). The authors do not clearly explain the logic behind this assertion, and multiple possibilities need to be considered. If dephosphorylation occurs after translation, then the Ser:pSer ratio should be unaffected by tRNA levels. If dephosphorylation occurs on the aminoacylated tRNA, then the effect on the Ser:pSer ratio depends on whether the phosphatase is saturated or not. If the phosphatase is saturated, the Ser:pSer ratio should decrease as the tRNA level increases because there will be more aminoacyl-pSer but the dephosphorylation rate does not change. If the phosphatase is not saturated, the Ser:pSer ratio will stay the same because the phosphatase will produce the same fraction of aminoacyl-Ser to aminoacyl-pSer. The observed Ser:pSer ratio does increase with increasing tRNA (Fig 4C), so the authors rule out model 1. It is critical that the authors supply a clear explanation of the logic, which appears to be much more complicated than implied by the brief description on lines 410-411.

As mentioned above in (4), models of OTS toxicity have been added to both the figures and the manuscript text to clearly define the opportunities for OTS mediated reductions in translational fidelity. The “mode” designation has been replaced with mechanistic descriptions.

With respect to this specific comment from the reviewer, it is likely that "Model 1" to contributes to some of the Ser observed in the reporter protein analysis. However, "Model 2" is less likely because tRNA^{pSer} lacks the major pre-requisite identity elements, namely the extended variable loop region, required for SerRS recognition. This point has been addressed in the manuscript and further illustrated in Figure 4. More importantly, if SerRS was misaminoacylating Ser onto tRNA^{pSer}, we would have observed substantial Ser incorporation during the tRNA-only expression experiments (Fig 4E & 4F). However, we did not detect any Ser incorporation in these experiments. Ser incorporation was only observed in the presence of both tRNA^{pSer} and pSerRS, and thus making "Model 2"-SerRS mischarging not the source of Ser misincorporation but rather the data support the mischarging of Ser by pSerRS as the source of small amounts of Ser observed.

As the reviewer points out, phosphatase activity alone cannot account for the tRNA-dependent proportional increase in Ser:pSer incorporation ratio, which should result in either a decrease or stabilized ratio dependent on phosphatase activity and concentration. This point has been added to the manuscript text during discussion of the results in Figure 4 (Lines 609-624). Additionally, if phosphatase activity in the host was indiscriminate and prolific, we would likely not have observed such a dramatic improvement in the yield and purity of pSer-protein reporters when implementing the optimized tRNA^{pSer}. Phosphatase activity on the pSer-protein should be agnostic to these changes in OTS architecture.

In the context of these experimental results, "Model 3" as outlined by the reviewer is the most plausible explanation. In previous work (Hauenstein *et al.*, 2008), pSerRS was demonstrated to recognize Ser as a substrate for aminoacylation and was able to produce 8-10% of the maximum aminoacyl-tRNA yield relative to cognate substrate pSer *in vitro*. This observation - coupled with previous observations that the diffusion rate of pSer-tRNA^{pSer} from pSerRS is the rate limiting step in aminoacylation – presents a scenario in which Ser-tRNA^{pSer} may be produced, albeit less efficiently, by pSerRS but may more readily diffuse from the active site. This could effectively increase the turnover rate for misacylated tRNA relative to pSer-tRNA^{pSer}.

6. Model 2 is that native SerRS misacylates tRNApSer with unphosphorylated Ser. As the authors note earlier with respect to Gly/Thr misincorporation (line 398), increasing tRNA levels could plausibly lead to higher levels of misacylation by a host aaRS enzyme. However, the authors note that "Ser misincorporation required both orthogonal aaRS and tRNA expression and was absent in the isolation of either component" (line 402), suggesting that host aaRS enzymes are not responsible for Ser misincorporation. The authors also note that based on limited homology between tRNASer and tRNApSer, the host SerRS enzyme is unlikely to act on tRNApSer. At this point in the manuscript, this logic seems plausible, leading the authors to conclude that Model 3, the only remaining model, is correct. However, Model 3 does not appear to be consistent with the data (see below).

7. Model 3 is that pSerRS can use unphosphorylated Ser as a substrate to acylate tRNApSer. The authors should make this statement clearly. They instead use vague language about "a specific interaction between pSerRS and tRNApSer which results in Ser misaminoacylation" (lines 411-412). More importantly, it is not immediately obvious why this model predicts that increases in tRNA levels should increase the Ser:pSer ratio. My expectation is that the Ser:pSer ratio would not be affected by increasing tRNA levels because pSerRS will most likely produce the same ratio of Ser misacylation to correct pSer acylation regardless of tRNA concentration.

Can the authors clarify this point? It is certainly possible that my logic is incorrect here, but the manuscript lacks a clear explanation that would allow the reader to evaluate this issue.

The manuscript has been revised to clearly state the mechanism of "Model 3". In addition to the explanation provided in response to reviewer comment #5 (above), the increase in Ser:pSer ratio can be further explained by a scenario in which the concentration pSer is limited in the amino acid pool compared to Ser, which is the case during growth in amino acid replete medium. Therefore, as you increase the amount of $tRNA^{pSer}$ available for aminoacylation, pSer is likely to be depleted before all the tRNA is aminoacylated, leaving Ser as a near-cognate substrate available for misacylation.

8. Data presented later in the manuscript is more consistent with Model 2 than Model 3. On line 440 the authors note that mutations to tRNA-pSer result in a decrease in Ser misincorporation. They write: "At the same time, a large decrease in Ser:pSer relative incorporation rates were also observed, suggesting that the sequence modification made to the orthogonal tRNA may have reduced Ser misaminoacylation (Figure 4E)." As noted by the authors in the context of Gly/Thr misincorporation (lines 428-434), this observation is most consistent with misincorporation due to host aaRS action (Model 2) because mutations in the tRNA could perturb misacylation by a host aaRS without necessarily affecting pSer incorporation by pSerRS. It is not obvious why the Ser:pSer ratio would decrease if Model 3 were correct. Once again, multiple possibilities need to be considered. If pSerRS residues involved in tRNA binding are not thermodynamically coupled to active site residues involved in catalysis, then the simplest expectation is that mutations in the tRNA will have the same effect on acylation with Ser or pSer, leaving the Ser:pSer ratio unchanged. Alternatively, it is possible that tRNA binding and catalysis are thermodynamically linked, so that mutations to the tRNA could have different effects on Ser vs. pSer acylation. Such effects could increase or decrease the Ser:pSer ratio, and it is not clear that there is a strong expectation either way. At minimum the authors must explicitly lay out the alternative models and corresponding expectations.

The manuscript has been revised to clearly state and explain the relevant alternatives and rationale behind the mechanism presented in "Model 3" (Lines 599-624). Based on the data in the present study as well as prior work, misacylation as a result of "Model 2" is highly unlikely. If "Model 2" was the source of Ser misincorporation, we would have observed Ser incorporation during our tRNA-only expression experiments (Fig 4E & 4F).

As mentioned in response to comment #5 , the affinity of pSerRS for $tRNA^{pSer}$ is altered pre- and post-aminoacylation and it is true that alteration of the tRNA sequence may generally affect the affinity of an aaRS:tRNA pair. This could be due to alteration of acceptor stem positioning in the active site relative to ATP or the amino acid. The decrease we observed in the relative ratio of Ser misincorporation following the mutation made to mitigate misacylation was relatively modest compared to the reductions observed for Gly and Thr. Although no changes were made to the pSerRS recognition elements on $tRNA^{pSer}$ nor to the tRNA binding domain of pSerRS, it is possible that $tRNA^{pSer}$ may no longer be optimally positioned for aminoacylation with Ser. A follow-up structural study would be necessary to confirm this mechanism and would be beyond the aims and scope of this manuscript.

9. Statements about the yield of recombinant pSer-containing protein are central to the manuscript, but the key comparison in the gel in Fig 1D is unclear. What exactly was loaded on the gel? Was it fixed amounts of cells? Was it equal volumes from each culture with different amounts of cells depending on the growth rate? Was it a clarified lysate normalized to the same

amount of total protein in each well? This information will indicate whether we are considering yield in the context of mg protein per liter of culture or fraction of protein relative to total protein. If gel samples were a fixed volume of culture, should the reader care about the decrease in growth rate from 1x to 4x tRNA if the absolute protein yield is apparently increasing?

An explanation of loading conditions and normalization for the relevant figure was added to the figure legend and methods section (Lines 857-858).

10. The importance and significance of Fig 5 is unclear. Was the optimized tRNApSer from Fig 4E used in the experiments in Fig 5? The text is vague but it seems like the optimized guide was not used. If so, Fig 5 and the corresponding sections seem to reiterate findings about expression levels that were detailed in prior sections. I encourage the authors to be as clear as possible about which tRNA is being used in Fig 5, and to consider restructuring the manuscript to place these experiments before the data shown in Fig 4.

To improve clarity surrounding the outcomes of this study, Figure 5 was remade, and additional experiments were performed to directly highlight and quantify the improvements mediated by OTS optimization (see Fig. 5 B, C and D).

Minor comments

1. On line 88, the authors write: "Although the study of the pSerOTS itself has been significant..." It is not clear what the authors mean by "significant".

This comment was addressed during the extensive revisions to the manuscript text.

2. On line 163, the authors refer to the Rop protein. The authors should explain what Rop is and how it affects plasmid copy number. No citation is provided for a general reader to understand what Rop is or what it is expected to do.

This comment was addressed during the extensive revisions to the manuscript text and further explanation was provided for Rop function and relevance.

3. On line 175, the authors write: "In comparison to E. coli lacking an OTS (WT), cells expressing glnS*-pSerRS showed a slight growth defect, while cells expressing TRC*-pSerRS displayed a significant decrease in growth (Figure 1E)." The text describes a comparison to WT but Figure 1E does not show the WT growth rate. It is possible to make comparisons to WT in panel 1C, but the comparison is based on subtle differences in green shading that are not easy to evaluate.

This comment was addressed during the extensive revisions to the manuscript text and through revision of Figure 1, as well as an additional supplementary table (Appendix Table S1).

4. On lines 178-183, the authors refer to comparisons between growth rates shown in Fig 1E that are within or barely outside the error bars. Can the authors clarify what constitutes a significant change in these values?

This comment was addressed during the extensive revisions to the manuscript text and through revision of Figure 1. Data from the previous figure 1E was moved to supplementary Expanded View Figure (Figure EV1C) and the significance was de-

emphasized (Lines 218-226) as this did not change any conclusions of the present study and was more quantitatively assessed in subsequent text (Lines 503-529) and Figure 4A-B.

5. On line 192, the authors write "Interestingly, the tRNA copy number dependent decrease in cellular viability was inversely proportional to the amount of non-pSer containing GFP reporter (Figure 1D). This observation suggests that tRNApSer misaminoacylation may play a role observed decrease in viability." Although the tRNApSer misaminoacylation idea is corroborated later in the manuscript, at this point the logical connection is not obvious. Can the authors rule out an alternative model where high tRNA levels cause burdens on host systems that perturb cell growth? An appropriate control would be to overexpress some unrelated tRNA to see how it affects pSer incorporation.

This comment was addressed during the extensive revisions to the manuscript text and through revision of Figure 4. We present potential models to explain tRNA-dependent toxicity. Based on our observation that tRNA copy number is inversely proportional to cellular toxicity, yet directly proportional to the increase in non-pSer containing reporter protein, we present misacylation by native aaRSs as the primary contributor to tRNA-mediated toxicity. We directly acknowledge that the other models presented may contribute to tRNA-mediated toxicity, with varying extent.

6. Similar questions arise for the effect of pSerRS levels on cell growth (Fig 1D/E). Are these effects specific to pSerRS or would they occur when any generic protein was expressed to a similar level? Do the authors have any growth measurements with generic protein overexpression controls for comparison to the pSerRS strains?

We thank the reviewer for this insightful question, as addressing this concern greatly improved the results of the current study. We performed additional mass spectrometry-based proteomics experiments to directly assess this question by comparing the proteomic response to overexpression of GFP to the proteomic response to the overexpression of pSerRS (presented in Figure 3A-D and Figure EV3). We identified a common core proteomic response to protein overexpression which allowed us to filter pSerRS-independent perturbations of the proteome and identify several putative pSerRS-specific mechanisms of aaRS-mediated toxicity.

7. On line 224, the authors write: "the abundance of pSerRS is likely to challenge the cell by disrupting the binding equilibrium within amino acid and tRNA substrate pools." It is not clear what "equilibrium" means in this context. Ref 39 cited by the authors does a reasonable job of using different language to make the same point more clearly.

This comment was addressed during the extensive revisions to the manuscript text and the specific language was adjusted based on Ref 39, as suggested.

8. What are the units of the x-axis in Fig 1E? Is it normalized to fold-change?

This comment was addressed during the extensive revisions of Figure 1.

9. On line 284, the authors write: "Overall, these data reinforce the notion that fine-tuning OTS component expression based on in-depth proteomic analysis can minimize the impact of an OTS on the host cell." The major comparisons are between just two systems, pSerOTSc and

pSerOTSlambda. These differences are meaningful and useful to delineate, but it seems like a stretch to call a comparison between two systems "fine-tuning".

We provided additional information in the manuscript text that clarify the analysis and comparisons made to other OTSs and OTS component expression experiments. Additional figures were added to illustrate the analysis across more than two systems.

10. Figure 3A is difficult to follow. (1) It would be helpful to include entries for putative pSer peptides in the second and third sets, even if they are below the limit of detection. (2) The alignment of sequences is confusing. The sequences inside the grey balls are barely readable without zooming far in, and it would help if those residues (and particularly the green ball) were lined up with the corresponding sequences below. Alternatively, the authors could remove the grey balls and simply highlight the residue of interest within each text string.

This comment was directly addressed during the extensive revisions of Figure 3. The specific figure component describe was moved to the supplemental material (Appendix Figure S8) and replaced by a figure panel (Figure 3J) with more direct illustration of the primary data.

11. In the MS-READ assay shown in Fig 3A, does pS incorporation occur at other Gly codons in the peptide? Can the authors explain briefly how MS-READ identifies misincorporation at a specific site? This manuscript refers to ref 48, which does not clearly address this question and refers to additional references in its own methods section.

This comment was addressed during the extensive revisions to the manuscript text. A description of MS-READ was included. It is likely that pSer incorporation is present at other codons within the reporter peptide. Due to technical limitations related to the mass resolution during data acquisition and downstream MS data analysis, these alternative misincorporated peptides are likely to be missed or assigned to the flexible codon position since they occur within the same tryptic fragment peptide. Accurate phosphosite localization is an ongoing and active field of study in our lab and others. We continue to explore improvements within the field that will enable direct resolution of this issue and have made steps towards this end through a recent publication:

Gassaway BM, Li J, Rad R, Mintseris J, *Mohler K*, Levy T, Aguiar M, Beausoleil SA, Paulo JA, *Rinehart J*, Huttlin EL, Gygi SP. A multi-purpose, regenerable, proteome-scale, human phosphoserine resource for phosphoproteomics. *Nat Methods.* 2022 Nov;19(11):1371-1375. Epub 2022 Oct 24. PMID: 36280721

12. On line 323, the authors write: "Without phospho-enrichment strategies, we found a total of 33 unique peptides..." How should this number be evaluated? Is 33 higher or lower than one might expect? The Fig 3C legend indicates that proteomes from pSerOTSlambda and lambda aaRS cells were search for misincorporation. The manuscript text is unclear about which cells were the source of the 33 peptides.

This comment was addressed during the extensive revisions to the manuscript text. Normally in E. coli without phosphoenrichment, only a few phosphopeptides are detected in our MS workflow and these peptides typically have low quality scores. The observation of 33 phosphopeptides without enrichment is significant and the source of these peptides was clarified in the text (Lines 463-467).

13. It is not clear how to interpret Fig 3C and the figure legend does not provide sufficient explanation. What are the red lines and how do they tell us about pSer incorporation?

This comment was directly addressed during the extensive revisions of Figure 3. The specific figure component describe was moved to the supplemental material and the description was clarified in the figure legend.

14. On line 325, the authors write: "The misincorporation of pSer at Gly codons is an important example of native proteome damage caused by an orthogonal aaRS and highlights importance of monitoring and tuning aaRS expression during OTS development." If the goal of OTSs is to produce proteins of interest with ncAA incorporation, and that goal can be achieved, why does it matter if there is misincorporation in the host genome? Do the authors expect that pSer misincorporation reduces yield of desired recombinant protein? Are there specific cases where incorporation of ncAAs has failed or produced low yield that can attributed to misincorporation in the host genome?

Additional text was added to the manuscript to highlight this potential mode of cytotoxicity (Lines 442-451). While it is true that most OTSs are deployed to produce recombinant proteins, the fitness of the host strain (as demonstrated in the present work) directly impacts recombinant phosphoprotein yield. Additionally, the observed damage to the host proteome that results from misincorporation is unlikely to be limited to host proteins and thus will also result in misincorporation into the recombinant protein itself during expression. These events would be difficult to assess in many cases due to the stochastic nature of the misincorporation event but would none-the-less be undesirable.

15. On line 344, the authors write: "We noticed that increasing the copy number of the suppressor tRNA while decreasing the levels of pSerRS led to an increase in non-phospho recombinant protein (Figure 1D)." It is difficult to evaluate this statement based on a gel image. Can the gel band intensities be quantified?

This comment was addressed during the extensive revisions to the manuscript text. We specifically indicate that these data are a qualitative preliminary assessment of incorporation fidelity (Line 515) and highlight the use of MS-READ to quantitatively assess these exact misincorporation events this with far greater resolution (briefly described in Lines 455-459 and more extensively in prior work, Ref. Mohler et al, 2017a.)

16. In Fig 4C, middle panel, the "ThrRS/pSerRS" label for tRNA identity elements should be "SerRS/pSerRS".

This comment was directly addressed during the extensive revisions of Figure 4 and is reflected in Figure 4I.

17. On line 442, the authors write: "A Phos-tagTM assay reflected the increase in recombinant protein purity quantified in the MS experiment (Figure 4E)." Fig 4E shows only a single gel lane for the optimized system. If the point is to show an increase, it would be helpful to show this sample side-by-side on the same gel with at least one of the original OTS systems. As noted above, it would also be helpful to explain what exactly is being loaded on the gel so it is clear whether the increase is relative to culture volume or total protein.

This comment was addressed during the extensive revisions to the manuscript text to explain the illustrative use of a single gel lane corresponding to the MS reporter used collect the quantitative misincorporation data and further clarified in figure legend of Figure 4.

18. The wording on lines 464-472 is confusing and it is difficult to parse which cell type is used with each vector

This comment was addressed during the extensive revisions to the manuscript text to clarify the cell type and vector.

19. On lines 514-516, the authors write: "The 6x tRNA OTS, for example, is robustly expressed (Lane 6, lower), but the proportion of phospho-mTOR production (Lane 6, upper) is significantly lower when compared to the 1x tRNA OTS counterpart (Lane1) (Figure 5E)." Is "robustly expressed" referring to mTOR or the OTS components? The text makes it seem that the OTS components have robust expression, but Fig 5E indicates that this point is in reference to mTOR.

This figure panel and corresponding data was removed from the manuscript during the revision process.

20. On line 515-516, the authors refer to Fig 5E, lane 6 but there is no lane 6 in Figure 5E. Do the authors mean lane 5?

This figure panel and corresponding data was removed from the manuscript during the revision process.

21. On line 582, the authors write: "Traditional methods of analyzing tRNA orthogonality fail to address this dynamic property of tRNA recognition and are therefore more susceptible to reductions in OTS fidelity due to cellular perturbation." What does this sentence mean? Most simply, there is a subject-verb problem here. The subject of the sentence appears to be "traditional methods of analyzing tRNA orthogonality". How does it make sense to say that traditional analysis methods more susceptible to reductions in fidelity? Again, issues like this are typically minor but occur so frequently in this manuscript that they severely impact its readability.

This comment was addressed during the extensive revisions to the manuscript text to improve clarity and readability. The specific point clarified here was that tRNA orthogonality is not assessed in the context of selectivity and the effect of changing intracellular orthogonal tRNA abundance within the host tRNA pool is often overlooked (Lines 443-451 and in the Discussion Section Lines 736-739).

22. Typos: line 48 "is often" should be "often", line 76 & 196 "it's" should be "its", missing words in the sentence on lines 194-195.

This comment was addressed during the extensive revisions to the manuscript text.

Reviewer #3:

Summary:

Mohler et al describe a thorough characterization of how an orthogonal translation system (OTS), specifically the pSerRS and tRNApSer system, can affect the viability, proteome, and tRNA pool of E. coli and use their results to improve pSer incorporation through amber suppression. First, they analyze how the expression of pSerRS and tRNApSer in two E. coli strain backgrounds, rEcoli and BL21, affect cell viability and protein expression. Strong overexpression of either OTS component was observed to reduce growth rate and accurate pSer incorporation into proteins, and that the growth rate reduction was more significant in BL21. By analyzing the proteome of rEcoli containing these OTS components, they find that cells where pSerRS is overexpressed upregulate translation-associated proteins, suggesting increased cellular burden. Lower expression of pSerRS from the weaker glnS promoter were not observed to similarly upregulate translation-associated proteins. Next, the authors analyzed the orthogonality of the pSer OTS towards native E. coli aaRSs and tRNAs using a mass-spectrometry based assay, MS-READ. They find that pSerRS misacylates native tRNAs, such as tRNAGly, that native aaRSs misacylate tRNApSer with Gly and Thr, and that pSerRS misacylates tRNApSer with Ser. The authors were able to successfully decrease Gly and Thr misacylation by engineering tRNA identity elements and observed reduced Ser misacylation as well. Finally, the authors optimize pSer incorporation into proteins of interest using their best OTS constructs, resulting in improved yields of phosphoproteins as analyzed by gel.

General remarks:

A systems-level understanding of how orthogonal translation systems interact with native E. coli translation components is of interest to the genetic code expansion community, as it would enable more deliberate engineering of orthogonal translation systems. The key data presented in this work are the characterizations that the authors used to understand these interactions. In particular are the proteomics of total E. coli protein of rEcoli, which provides data on how it must redirect resources towards producing the OTS and the protein of interest, and MS-READ, which is a mass-spectrometry based technique previously developed by the authors to decipher interactions between aaRSs, tRNAs, and amino acids. The advances made in this work result in an OTS that appears to have less cellular burden and higher specificity for pSer incorporation.

However, the proteomics data does not appear to have informed the design of orthogonal translation systems, as it appears to have followed the optimization in Figure 1, and it does not seem to provide novel conclusions, as most of the effects are related to genes linked to protein overexpression, which seems intuitive. This was disappointing. With further analysis, described below, I believe this could be a useful data set for the genetic code expansion community more broadly.

The experiments surrounding tRNA engineering using MS-READ thoroughly analyze the fidelity of their OTS. A follow up experiment, described below, should be conducted to characterize the amino acid specificity of pSerRS. Generally, however, I believe this section is well done and describes a strong method to analyze pSer incorporation fidelity.

Overall, we thank the reviewer for their careful review of the manuscript and suggestions. We substantially revised the manuscript text and figures to improve the clarity, content, and structure. Additional MS experiments and more detailed proteomic analyses were performed to directly address concerns raised regarding the novelty of the conclusions of the present study. We hope that the changes leave the reviewer with less disappointment regarding the proteomic analysis.

Major points:

It is unclear if the proteomics data, particularly for constructs expressing pSerRS, is specific to pSerRS and thus provides insight for engineering organisms with expanded genetic codes, or is indicative of more general cellular consequences due to protein overexpression. A useful experiment would be to compare the proteomics data with data from a rEcoli strain that contains similar constructs as pSerOTSc and Lambda but expresses a "dummy" protein that does not interact with native translation machinery. This would clarify if these proteome changes are indicative of unique interactions between the pSerOTS and native translation machinery rather than the cellular burden of protein overexpression. Put another way, is this a systems effect?

We performed additional mass spectrometry-based proteomics experiments to directly assess this question by comparing the proteomic response to overexpression of GFP to the proteomic response to the overexpression of pSerRS (Figure 3A-D and Figure EV3). We identified a common core proteomic response to protein overexpression which allowed us to filter pSerRS-independent perturbations of the proteome and identify several putative pSerRS-specific mechanisms of aaRS-mediated toxicity.

The section between lines 343 -392 would benefit from a further experiment on the specificity of the pSerRS. One possibility for misincorporation that is not addressed in the current text is the polyspecificity of engineered aaRSs. This could be tested by challenging rEcoli containing pSerRS and its cognate tRNA to make a reporter in the absence of Sep and could be analyzed using MS-READ. Any reporter protein synthesized that is not found in the data analyzing near-cognate UAG suppression would then likely be a result of pSerRS polyspecificity towards other amino acids. This would further support conclusions that native aaRSs are misacylating tRNApSer by showing the specificity of pSerRS.

We, and others, and found that it is nearly impossible to eliminate pSer from the amino acid pool and that pSerRS will successfully scavenge some amount of pSer to use for aminoacylation. However, we do observe some degree of polyspecificity through the incorporation of Ser which is only observed in the presence of both the aaRS and tRNA (see comments to reviewer 2 above). This observation is consistent with prior work demonstrating the ability of pSerRS to recognize Ser as a substrate for aminoacylation and was able to produce 8-10% of the maximum aminoacyl-tRNA yield relative to cognate substrate pSer *in vitro*. Additionally, eliminating misaminoacylation by host GlyRS and ThrRS resulted in Ser being the only remaining significant miscorporation event. We do not believe this Ser is attributable to phosphatase activity and we have added a detailed explanation of this reasoning to the manuscript text.

It is unclear why the tRNAOptpSer and pSerOTSc* that was engineered in Figure 4 did not appear to be used in the final experiments in Figure 5. A discussion clarifying this decision would be appreciated. Even better, the experiments in Figure 5 could be re-done if it will impact the story.

This comment was addressed during the extensive revisions to the manuscript text and through revision of Figure 5 to include a direct comparison of OTS variants and relative improvements.

Please include phosphoprotein yields (g/L), as this is a useful metric that would help guide the choice of OTS for a particular experiment, and help others assess the usefulness of the work for

those that want to make phosphoproteins.

Additional experiments were performed to directly address this question. Quantitative comparison of phosphoprotein yield and purity were made across OTS variants and added to Figure 5.

Minor points

- The source of Sep in these experiments is unclear. Please clarify if Sep is biosynthesized in vivo or if exogenous Sep was supplemented into growth medium.

This comment was addressed during the extensive revisions to the manuscript text. We specifically clarify the source of phosphoserine through the accumulation of pSer (a metabolic intermediate during Ser biosynthesis) following deletion of the phosphatase serB which is responsible for the final conversion of pSer to Ser.

- Names for appropriate strains should be clearly defined early in the text. For example, "Lambda" is used in Figure 1C but is not defined until Line 234.

Each figure and the manuscript text has been substantially revised to improve the clarity and consistency of OTS description.

- Figure 1C could also be improved by clearly defining each condition. For example, the first column in Figure 1C is described both as "No OTS" and "glnS*-aaRS," which is confusing.

This comment was addressed during the extensive revisions to the manuscript text and revisions to Figure 1. This specific section of Figure 1C was moved to Expanded View Figure EV1 and additionally, presented in a supplementary table (Appendix Table S1) to improve clarity.

- A color bar on Figure 1C and a label clearly showing that numbers Figure 2A represent rank would improve readability.

This comment was addressed during the extensive revisions to the manuscript text and Figures.

- The data in Supplementary Figures 3 and 4 would be better served by a table containing the proteins that were significantly up/downregulated.

A file containing these results presented as a user accessible table has been added as a supplementary data file (Appendix Dataset S1 and S2).

- The sentence starting on line 469 ("Lanes 2 and 3...") should reference a figure.

This comment was addressed during the extensive revisions to the manuscript text and Figures.

5th Jun 2023

Manuscript Number: MSB-2021-10591R
Title: System-wide Optimization of an Orthogonal Translation System with Enhanced Biological Tolerance

Dear Jesse,

Thank you for sending us your revised manuscript. We have now heard back from the two reviewers who were asked to evaluate your revised study. As you will see below, both reviewers think that the study has improved as a result of the performed revisions. However, reviewer #2 still lists a few remaining concerns, which we would ask you to address in a minor revision.

We would also ask you to address some remaining editorial issues listed below.

- Our data editors have noticed some unclear or missing information in the figure legends, please see the attached .doc file. Please make all requested text changes using the attached file and *keeping the "track changes" mode* so that we can easily access the edits made.

- Please make sure that the funding information (e.g. grant numbers) is correct both in the submission system and in the manuscript file.

- The Reference Perez-Riverol Y et al (2019) 'The PRIDE database and related tools and resources in 2019: improving support for quantification data' should be corrected: currently 20 authors are listed followed by et al, but our Reference style allows 10 authors followed by et al.

- Please include a callout to Fig. EV2E-F in the main text.

- Appendix Dataset S1-S2 should be renamed to Dataset EV1-EV2 and their callouts in the text should be corrected accordingly. The descriptions of the two Datasets should be removed from the Appendix PDF.

- The first lane of the WB shown in Fig. S9A (P1) is reused in S9B. This needs to be clearly indicated in the figure legend.

- The synopsis image is rather large and detailed and not all labels display well at the final required size. Please resupply the image (it needs to be exactly 550 px wide, and the height ideally < 500 px), ensuring that all labels are legible.

Please resubmit your revised manuscript online, with a covering letter listing amendments and responses to each point raised by the referees. Please resubmit the paper **within one month** and ideally as soon as possible. If we do not receive the revised manuscript within this time period, the file might be closed and any subsequent resubmission would be treated as a new manuscript. Please use the Manuscript Number (above) in all correspondence.

Click on the link below to submit your revised paper.

https://msb.msubmit.net/cgi-bin/main.plex

As a matter of course, please make sure that you have correctly followed the instructions for authors as given on the submission website.

Thank you for submitting this paper to Molecular Systems Biology.

Kind regards,

Maria

Maria Polychronidou, PhD
Senior Editor
Molecular Systems Biology

If you do choose to resubmit, please click on the link below to submit the revision online before 5th Jul 2023.

https://msb.msubmit.net/cgi-bin/main.plex

IMPORTANT:
Please note that corresponding authors are required to supply an ORCID ID for their name upon submission of a revised manuscript (EMBO Press signed a joint statement to encourage ORCID adoption).
(https://www.embopress.org/page/journal/17444292/authorguide#editorialprocess)
Currently, our records indicate that the ORCID for your account is 0000-0003-4839-4005.

Please click the link below to modify this ORCID:
Link Not Available

The system will prompt you to fill in your funding and payment information. This will allow Wiley to send you a quote for the article processing charge (APC) in case of acceptance. This quote takes into account any reduction or fee waivers that you may be eligible for. Authors do not need to pay any fees before their manuscript is accepted and transferred to the publisher.

*** PLEASE NOTE *** As part of the EMBO Press transparent editorial process initiative (see our Editorial at https://dx.doi.org/10.1038/msb.2010.72 , Molecular Systems Biology will publish online a Review Process File to accompany accepted manuscripts. When preparing your letter of response, please be aware that in the event of acceptance, your cover letter/point-by-point document will be included as part of this File, which will be available to the scientific community. More information about this initiative is available in our Instructions to Authors. If you have any questions about this initiative, please contact the editorial office (msb@embo.org).

Reviewer #2:

Summary

The authors have satisfactorily addressed most of the major concerns raised in the initial review with new experiments and revised explanations. The manuscript should be published after the authors consider the comments below.

Major comments

1. The description of alternative models for Ser misincorporation is improved (lines 559-575), but key points still need to be clarified. First, the language the authors use to explain steady-state outcomes from phosphatase activity is confusing at best. On line 564, the authors write: "In the first scenario, where phosphatase activity exceeds the rate of phosphoprotein production, we would again expect to see a constant ratio of Ser:pSer dictated by the steady-state catalytic activity of the phosphatase." This statement does not make sense because phosphatase rate depends on the substrate concentration and whether the enzyme is saturated. If the phosphatase rate exceeds the rate of phosphoprotein production, then the Ser:pSer ratio will increase (i.e. pSer levels decrease) until the system reaches a steady state where the flux in to the pSer state equals the flux out. To relate this analysis to the observed data, the authors should explain how the steady state Ser:pSer ratio changes with increasing tRNA-pSer expression (Figure 4L). The statement starting on line 564 does not explain why the Ser:pSer ratio should be constant with increasing tRNA-pSer expression.

2. A second critical point is that the revised manuscript does not include an explanation for why Ser incorporation caused by pSerRS-mediated Ser misactivation (line 574) would lead to the observed increase in Ser:pSer ratio with tRNA-pSer expression. The authors did make a plausible case in their response to the reviews (pSer depleted first), and it would be helpful to make this point in the revised manuscript.

Minor comments

1. Line 166: "Additionally, the average cell size (and independent measure of stress) reduced nearly 3-fold (from 93.2 to 31.1, Figure 1C)," As written this statement is confusing because the numbers lack units. Also presumably "and" should be "an" here.

2. Line 379: "we found that the enzymes responsible for degrading AMP (Amn) and pyrophosphate (Ppa) are up-regulated during high-level pSerRS expression (TRC*-pSerRS and [λ]) but not during low-level expression from [C1] (Figure 3F)." Can the authors also compare these enzyme levels for high-level pSerRS expression vs. high level GFP expression? The comparisons to high-level GFP expression in Figure 3A-D were informative and it would be helpful to continue these comparisons throughout the analysis.

3. Figure 3I: What is "pSerRS-GFP"? Based on the manuscript text (line 395) presumably this label should be "TRC*-GFP". Also note that the helpful comparison to TRC*-GFP in Figure 3I highlights the lack of the same comparison in Figure 3F.

4. Line 518: "When matched to host fitness and OTS fidelity across the same OTS series, these data indicate that the host

fitness and OTS fidelity reductions that we observe in Figure 4A-B result from tRNApSer misrecognition (and misaminoacylation) by the host Gly- and ThrRS." Can the authors elaborate on how they reached the conclusion about host fitness? The conclusion about OTS fidelity seems clear, but to make conclusions about host fitness it seems like the authors should have a comparison to overexpression of another tRNA or a mutant tRNA that is defective for aminoacylation to distinguish effects from expression burden of extra tRNA or other effects not related to misrecognition by Gly- and ThrRS. These experiments are not necessary for publication. The authors could either clarify their reasoning or modify the conclusion.

Reviewer #3:

The authors have addressed my concerns. It is a nice piece and is meaningful for the field.

Point-by-Point Response to Reviewer Comments (2nd Revision)
Our responses in Blue
Original reviewer comments in black

Editorial Revisions:

We would also ask you to address some remaining editorial issues listed below.

- Our data editors have noticed some unclear or missing information in the figure legends, please see the attached .doc file. Please make all requested text changes using the attached file and *keeping the "track changes" mode* so that we can easily access the edits made.

All requested text changes have been completed in the manuscript file with "track changes" mode enabled.

- Please make sure that the funding information (e.g. grant numbers) is correct both in the submission system and in the manuscript file.

We have provided updated funding information for the manuscript and the online submission system.

- The Reference Perez-Riverol Y et al (2019) 'The PRIDE database and related tools and resources in 2019: improving support for quantification data' should be corrected: currently 20 authors are listed followed by et al, but our Reference style allows 10 authors followed by et al.

This reference format has been corrected to reduce the number of listed authors to 10.

- Please include a callout to Fig. EV2E-F in the main text.

The callout for Fig. EV2E-F has been introduced into the main text.

- Appendix Dataset S1-S2 should be renamed to Dataset EV1-EV2 and their callouts in the text should be corrected accordingly. The descriptions of the two Datasets should be removed from the Appendix PDF.

Appendix Dataset S1-S2 have been renamed and their callouts have been adjusted. The dataset description was removed from the PDF.

- The first lane of the WB shown in Fig. S9A (P1) is reused in S9B. This needs to be clearly indicated in the figure legend.

The figure legend for Fig. S9 was modified to directly state the re-use of a portion of the immunoblot image from S9A in S9B.

- The synopsis image is rather large and detailed and not all labels display well at the final required size. Please resupply the image (it needs to be exactly 550 px wide, and the height ideally < 500 px), ensuring that all labels are legible.

The synopsis image has been edited to ensure clarity and readability at the required size.

Reviewer #2:

We would like to thank Reviewer # 2 for providing additional feedback. To address the concerns below, we have expanded explanation in the manuscript text and modified figure 3 to add the requested data.

Major comments

1. The description of alternative models for Ser misincorporation is improved (lines 559-575), but key points still need to be clarified. First, the language the authors use to explain steady-state outcomes from phosphatase activity is confusing at best. On line 564, the authors write: "In the first scenario, where phosphatase activity exceeds the rate of phosphoprotein production, we would again expect to see a constant ratio of Ser:pSer dictated by the steady-state catalytic activity of the phosphatase." This statement does not make sense because phosphatase rate depends on the substrate concentration and whether the enzyme is saturated. If the phosphatase rate exceeds the rate of phosphoprotein production, then the Ser:pSer ratio will increase (i.e. pSer levels decrease) until the system reaches a steady state where the flux in to the pSer state equals the flux out. To relate this analysis to the observed data, the authors should explain how the steady state Ser:pSer ratio changes with increasing tRNA-pSer expression (Figure 4L). The statement starting on line 564 does not explain why the Ser:pSer ratio should be constant with increasing tRNA-pSer expression.

Additional text has been added to the manuscript to address reviewer feedback. In this system, we know that $tRNA^{pSer}$ is not a rate-limiting substrate for aminoacylation by pSerRS because increasing tRNA expression leads to an increase in misacylated $tRNA^{pSer}$ rather than a direct increase in phosphoprotein yield. Thus, for a fixed rate of phosphoprotein production (independent of tRNA concentration) under phosphatase saturating conditions, the steady-state equilibrium of Ser:pSer would be dictated by the rate of phosphoprotein production and the rate of dephosphorylation by the phosphatase which should remain constant even as the intracellular concentration tRNA is increased.

Line 531-536: "In the first scenario under phosphatase saturating conditions, where phosphatase activity exceeds the rate of phosphoprotein production, we would expect to see a fixed steady-state ratio of Ser:pSer dictated by the equilibrium between phosphoprotein production and the catalytic activity of the phosphatase. Under these conditions, the steady-state equilibrium should not be tRNA-dependent unless the rate of phosphoprotein production is enhanced by an increase in tRNA such that it exceeds the catalytic capacity of the phosphatase."

2. A second critical point is that the revised manuscript does not include an explanation for why Ser incorporation caused by pSerRS-mediated Ser misactivation (line 574) would lead to the observed increase in Ser:pSer ratio with tRNA-pSer expression. The authors did make a plausible case in their response to the reviews (pSer depleted first), and it would be helpful to make this point in the revised manuscript.

Additional text has been added to the manuscript to address reviewer feedback.

Line 545-550: "In this context, Ser misactivation would be enhanced if the concentration pSer was limited in the amino acid pool relative to the concentration of Ser. This scenario would affect the selectivity of pSerRS such that any increase in the amount of tRNApSer

available for aminoacylation, following pSer pool depletion, is likely to result in misacylation with lower-specificity near-cognate amino acid substrates like Ser and an increase in the relative ratio of Ser:pSer that is proportional to tRNApSer expression."

Minor comments

1. Line 166: "Additionally, the average cell size (and independent measure of stress) reduced nearly 3-fold (from 93.2 to 31.1, Figure 1C)," As written this statement is confusing because the numbers lack units. Also presumably "and" should be "an" here.

The manuscript text has been edited to clarify units and correct the typographical error.

2. Line 379: "we found that the enzymes responsible for degrading AMP (Amn) and pyrophosphate (Ppa) are up-regulated during high-level pSerRS expression (TRC*-pSerRS and [λ]) but not during low-level expression from [C1] (Figure 3F)." Can the authors also compare these enzyme levels for high-level pSerRS expression vs. high level GFP expression? The comparisons to high-level GFP expression in Figure 3A-D were informative and it would be helpful to continue these comparisons throughout the analysis.

Figure 3F-G has been modified to include data from the proteomic comparison made in Figure 3D (pSerRS – GFP). The manuscript text and figure legend has been edited to clarify that (pSerRS – GFP) represents proteomic perturbations within the high-level pSerRS expression proteome that are significantly different from the proteomes of cells expressing high levels of GFP.

3. Figure 3I: What is "pSerRS-GFP"? Based on the manuscript text (line 395) presumably this label should be "TRC*-GFP". Also note that the helpful comparison to TRC*-GFP in Figure 3I highlights the lack of the same comparison in Figure 3F.

Alongside revisions to address comment 2 (above), Figure 3I has been modified to include data from the proteomic comparison made in Figure 3D (pSerRS – GFP). The manuscript text and figure legend has been edited to clarify that (pSerRS – GFP) represents proteomic perturbations within the high-level pSerRS expression proteome that are significantly different from the proteomes of cells expressing high levels of GFP.

4. Line 518: "When matched to host fitness and OTS fidelity across the same OTS series, these data indicate that the host fitness and OTS fidelity reductions that we observe in Figure 4A-B result from tRNApSer misrecognition (and misaminoacylation) by the host Gly- and ThrRS." Can the authors elaborate on how they reached the conclusion about host fitness? The conclusion about OTS fidelity seems clear, but to make conclusions about host fitness it seems like the authors should have a comparison to overexpression of another tRNA or a mutant tRNA that is defective for aminoacylation to distinguish effects from expression burden of extra tRNA or other effects not related to misrecognition by Gly- and ThrRS. These experiments are not necessary for publication. The authors could either clarify their reasoning or modify the conclusion.

To address reviewer feedback, additional text has been added to the manuscript to specifically state that additional experiments would be required to reinforce conclusions about host fitness

Line 484-488: "…these data indicate that the host fitness and OTS fidelity reductions that

we observe in Figure 4A-B may result from tRNApSer misrecognition (and misaminoacylation) by the host Gly- and ThrRS. However, additional experiments involving a broader range of tRNA mutants will be required to concretely establish this causal relationship."

Reviewer #3:

The authors have addressed my concerns. It is a nice piece and is meaningful for the field.

3rd Jul 2023

Manuscript Number: MSB-2021-10591RR
Title: System-wide Optimization of an Orthogonal Translation System with Enhanced Biological Tolerance

Dear Jesse,

Thank you for sending us your revised manuscript. We are now satisfied with the performed revisions and I am glad to inform you that we can accept your manuscript for publication, once the remaining editorial points listed below have been addressed.

- The References in the Appendix should be formatted according to the Molecular Systems Biology reference style i.e. ordered alphabetically and listing the first 10 authors followed by et al.

Please resubmit your revised manuscript online **within two weeks** and ideally as soon as possible. If we do not receive the revised manuscript within this time period, the file might be closed and any subsequent resubmission would be treated as a new manuscript. Please use the Manuscript Number (above) in all correspondence.

Click on the link below to submit your revised paper.

https://msb.msubmit.net/cgi-bin/main.plex

Thank you for submitting this paper to Molecular Systems Biology.

Kind regards,

Maria

Maria Polychronidou, PhD
Senior Editor
Molecular Systems Biology

If you do choose to resubmit, please click on the link below to submit the revision online before 2nd Aug 2023.

https://msb.msubmit.net/cgi-bin/main.plex

The system will prompt you to fill in your funding and payment information. This will allow Wiley to send you a quote for the article processing charge (APC) in case of acceptance. This quote takes into account any reduction or fee waivers that you may be eligible for. Authors do not need to pay any fees before their manuscript is accepted and transferred to the publisher.

*** PLEASE NOTE *** As part of the EMBO Press transparent editorial process initiative (see our Editorial at https://dx.doi.org/10.1038/msb.2010.72 , Molecular Systems Biology will publish online a Review Process File to accompany accepted manuscripts. When preparing your letter of response, please be aware that in the event of acceptance, your cover letter/point-by-point document will be included as part of this File, which will be available to the scientific community. More information about this initiative is available in our Instructions to Authors. If you have any questions about this initiative, please contact the editorial office (msb@embo.org).

All editorial and formatting issues were resolved by the authors.

5th Jul 2023

Manuscript number: MSB-2021-10591RRR
Title: System-wide Optimization of an Orthogonal Translation System with Enhanced Biological Tolerance

Dear Jesse,

Thank you again for sending us your revised manuscript. We are now satisfied with the modifications made and I am pleased to inform you that your paper has been accepted for publication.

*** PLEASE NOTE *** As part of the EMBO Publications transparent editorial process initiative (see our Editorial at https://dx.doi.org/10.1038/msb.2010.72), Molecular Systems Biology publishes online a Review Process File with each accepted manuscripts. This file will be published in conjunction with your paper and will include the anonymous referee reports, your point- by-point response and all pertinent correspondence relating to the manuscript. If you do NOT want this File to be published, please inform the editorial office at msb@embo.org within 14 days upon receipt of the present letter.

Should you be planning a Press Release on your article, please get in contact with msb@wiley.com as early as possible, in order to coordinate publication and release dates.

LICENSE AND PAYMENT:
All articles published in Molecular Systems Biology are fully open access: immediately and freely available to read, download and share.

Molecular Systems Biology charges an article processing charge (APC) to cover the publication costs. You, as the corresponding author for this manuscript, should have already received a quote with the article processing fee separately. Please let us know in case this quote has not been received.

Once your article is at Wiley for editorial production you will receive an email from Wiley's Author Services system, which will ask you to log in and will present you with the publication license form for completion. Within the same system the publication fee can be paid by credit card, an invoice or pro forma can be requested.

Payment of the publication charge and the signed Open Access Agreement form must be received before the article can be published online.

Molecular Systems Biology articles are published under the Creative Commons licence CC BY, which facilitates the sharing of scientific information by reducing legal barriers, while mandating attribution of the source in accordance to standard scholarly practice.

Proofs will be forwarded to you within the next 2-3 weeks.

Thank you very much for submitting your work to Molecular Systems Biology.

Kind regards,

Maria

Maria Polychronidou, PhD
Senior Editor
Molecular Systems Biology

EMBO Press Author Checklist

Corresponding Author Name: Jesse Rinehart
Journal Submitted to: Molecular Systems Biology
Manuscript Number: MSB-2021-10591

USEFUL LINKS FOR COMPLETING THIS FORM
The EMBO Journal - Author Guidelines
EMBO Reports - Author Guidelines
Molecular Systems Biology - Author Guidelines
EMBO Molecular Medicine - Author Guidelines

Reporting Checklist for Life Science Articles (updated January
This checklist is adapted from Materials Design Analysis Reporting (MDAR) Checklist for Authors. MDAR establishes a minimum set of requirements in transparent reporting in the life sciences (see Statement of Task: 10.31222/osf.io/9sm4x). Please follow the journal's guidelines in preparing your
Please note that a copy of this checklist will be published alongside your article.

Abridged guidelines for figures
1. Data
The data shown in figures should satisfy the following conditions:
- the data were obtained and processed according to the field's best practice and are presented to reflect the results of the experiments in an accurate and unbiased manner.
- ideally, figure panels should include only measurements that are directly comparable to each other and obtained with the same assay.
- plots include clearly labeled error bars for independent experiments and sample sizes. Unless justified, error bars should not be shown for technical
- if n<5, the individual data points from each experiment should be plotted. Any statistical test employed should be justified.
- Source Data should be included to report the data underlying figures according to the guidelines set out in the authorship guidelines on Data

2. Captions
Each figure caption should contain the following information, for each panel where they are relevant:
- a specification of the experimental system investigated (eg cell line, species name).
- the assay(s) and method(s) used to carry out the reported observations and measurements.
- an explicit mention of the biological and chemical entity(ies) that are being measured.
- an explicit mention of the biological and chemical entity(ies) that are altered/varied/perturbed in a controlled manner.
- the exact sample size (n) for each experimental group/condition, given as a number, not a range;
- a description of the sample collection allowing the reader to understand whether the samples represent technical or biological replicates (including how many animals, litters, cultures, etc.).
- a statement of how many times the experiment shown was independently replicated in the laboratory.
- definitions of statistical methods and measures:
  - common tests, such as t-test (please specify whether paired vs. unpaired), simple χ2 tests, Wilcoxon and Mann-Whitney tests, can be unambiguously identified by name only, but more complex techniques should be described in the methods section;
  - are tests one-sided or two-sided?
  - are there adjustments for multiple comparisons?
  - exact statistical test results, e.g., P values = x but not P values < x;
  - definition of 'center values' as median or average;
  - definition of error bars as s.d. or s.e.m.

Please complete ALL of the questions below.
Select "Not Applicable" only when the requested information is not relevant for your study.

Materials

Newly Created Materials	Information included in the manuscript?	In which section is the information available? (Reagents and Tools Table, Materials and Methods, Figures, Data Availability Section)
New materials and reagents need to be available; do any restrictions apply?	Yes	Materials and Methods: The optimized pSerOTS [C1*] andstrain are available via Addgene (pSerOTS-C1*, #188537; rEcoliXpS, #192872).

Antibodies	Information included in the manuscript?	In which section is the information available? (Reagents and Tools Table, Materials and Methods, Figures, Data Availability Section)
For **antibodies** provide the following information:<br>- Commercial antibodies: RRID (if possible) or supplier name, catalogue number and or/clone number<br>- Non-commercial: RRID or citation	Not Applicable	N/A

DNA and RNA sequences	Information included in the manuscript?	In which section is the information available? (Reagents and Tools Table, Materials and Methods, Figures, Data Availability Section)
**Short novel DNA or RNA including primers, probes**: provide the sequences.	Not Applicable	N/A

Cell materials	Information included in the manuscript?	In which section is the information available? (Reagents and Tools Table, Materials and Methods, Figures, Data Availability Section)
**Cell lines:** Provide species information, strain. Provide accession number in repository **OR** supplier name, catalog number, clone number, and/**OR** RRID.	Not Applicable	N/A
**Primary cultures:** Provide species, strain, sex of origin, genetic modification status.	Not Applicable	N/A
Report if the cell lines were recently **authenticated** (e.g., by STR profiling) and tested for mycoplasma contamination.	Not Applicable	N/A

Experimental animals	Information included in the manuscript?	In which section is the information available? (Reagents and Tools Table, Materials and Methods, Figures, Data Availability Section)
**Laboratory animals or Model organisms:** Provide species, strain, sex, age, genetic modification status. Provide accession number in repository **OR** supplier name, catalog number, clone number, **OR** RRID.	Not Applicable	N/A
**Animal observed in or captured from the field:** Provide species, sex, and age where possible.	Not Applicable	N/A
Please detail **housing and husbandry conditions**.	Not Applicable	N/A

Plants and microbes	Information included in the manuscript?	In which section is the information available? (Reagents and Tools Table, Materials and Methods, Figures, Data Availability Section)
**Plants:** provide species and strain, ecotype and cultivar where relevant, unique accession number if available, and source (including location for collected wild specimens).	Not Applicable	N/A
**Microbes:** provide species and strain, unique accession number if available, and source.	Yes	Materials and Methods

Human research participants	Information included in the manuscript?	In which section is the information available? (Reagents and Tools Table, Materials and Methods, Figures, Data Availability Section)
If collected and within the bounds of privacy constraints report on age, sex and gender or ethnicity for all study participants.	Not Applicable	N/A

Core facilities	Information included in the manuscript?	In which section is the information available? (Reagents and Tools Table, Materials and Methods, Figures, Data Availability Section)
If your work benefited from core facilities, was their service mentioned in the acknowledgments section?	Yes	Materials and Methods

Design

Study protocol	Information included in the manuscript?	In which section is the information available? (Reagents and Tools Table, Materials and Methods, Figures, Data Availability Section)
If study protocol has been **pre-registered, provide DOI in the manuscript**. For clinical trials, provide the trial registration number **OR** cite DOI.	Not Applicable	N/A
Report the **clinical trial registration number** (at ClinicalTrials.gov or equivalent), where applicable.	Not Applicable	N/A

Laboratory protocol	Information included in the manuscript?	In which section is the information available? (Reagents and Tools Table, Materials and Methods, Figures, Data Availability Section)
Provide DOI OR other citation details if **external detailed step-by-step protocols** are available.	Not Applicable	N/A

Experimental study design and statistics	Information included in the manuscript?	In which section is the information available? (Reagents and Tools Table, Materials and Methods, Figures, Data Availability Section)
Include a statement about **sample size** estimate even if no statistical methods were used.	Yes	Figures: Unless otherwise noted, n = 3 independent replicates. Direct sample size calculations were not relevant for this study.
Were any steps taken to minimize the effects of subjective bias when allocating animals/samples to treatment (e.g. **randomization procedure**)? If yes, have they been described?	Not Applicable	Randomization procedures were not applicable to this study.
Include a statement about **blinding** even if no blinding was done.	Not Applicable	Blinding was not applicable to this study.
Describe **inclusion/exclusion criteria** if samples or animals were excluded from the analysis. Were the criteria pre-established?<br><br>If sample or data points were omitted from analysis, report if this was due to attrition or intentional exclusion and provide justification.	Not Applicable	Inclusion/exclusion criteria were not established for this study and no samples were omitted from individual experiments.
For every figure, are **statistical tests** justified as appropriate? Do the data meet the assumptions of the tests (e.g., normal distribution)? Describe any methods used to assess it. Is there an estimate of variation within each group of data? Is the variance similar between the groups that are being statistically compared?	Yes	Figures and Materials and Methods: Statistical analyses described throughout the manuscript were justified according to normal conventions.

Sample definition and in-laboratory replication	Information included in the manuscript?	In which section is the information available? (Reagents and Tools Table, Materials and Methods, Figures, Data Availability Section)
In the figure legends: state number of times the experiment was **replicated** in laboratory.	Yes	Figures
In the figure legends: define whether data describe **technical or biological replicates**.	Yes	Figures

Ethics

Ethics	Information included in the manuscript?	In which section is the information available? (Reagents and Tools Table, Materials and Methods, Figures, Data Availability Section)
Studies involving **human participants**: State details of **authority granting ethics approval** (IRB or equivalent committee(s), provide reference number for approval.	Not Applicable	N/A
Studies involving **human participants**: Include a statement confirming that **informed consent** was obtained from all subjects and that the experiments conformed to the principles set out in the WMA Declaration of Helsinki and the Department of Health and Human Services Belmont Report.	Not Applicable	N/A
Studies involving **human participants:** For publication of **patient photos**, include a statement confirming that consent to publish was obtained.	Not Applicable	N/A
Studies involving experimental **animals**: State details of **authority granting ethics approval** (IRB or equivalent committee(s), provide reference number for approval. Include a statement of compliance with ethical regulations.	Not Applicable	N/A
Studies involving **specimen and field samples:** State if relevant **permits** obtained, provide details of authority approving study; if none were required, explain why.	Not Applicable	N/A

Dual Use Research of Concern (DURC)	Information included in the manuscript?	In which section is the information available? (Reagents and Tools Table, Materials and Methods, Figures, Data Availability Section)
Could your study fall under dual use research restrictions? Please check biosecurity documents and list of **select agents and toxins** (CDC): https://www.selectagents.gov/sat/list.htm	Not Applicable	N/A
If you used a select agent, is the security level of the lab appropriate and reported in the manuscript?	Not Applicable	N/A
If a study is subject to dual use research of concern regulations, is the name of the **authority granting approval and reference number** for the regulatory approval provided in the manuscript?	Not Applicable	N/A

Reporting

The MDAR framework recommends adoption of discipline-specific guidelines, established and endorsed through community initiatives. Journals have their own policy about requiring specific guidelines and recommendations to complement MDAR.

Adherence to community standards	Information included in the manuscript?	In which section is the information available? (Reagents and Tools Table, Materials and Methods, Figures, Data Availability Section)
State if relevant guidelines or checklists (e.g., **ICMJE, MIBBI, ARRIVE, PRISMA**) have been followed or provided.	Not Applicable	N/A
For **tumor marker prognostic studies,** we recommend that you follow the **REMARK** reporting guidelines (see link list at top right). See author guidelines, under 'Reporting Guidelines'. Please confirm you have followed these guidelines.	Not Applicable	N/A
For **phase II and III randomized controlled trials**, please refer to the **CONSORT** flow diagram (see link list at top right) and submit the CONSORT checklist (see link list at top right) with your submission. See author guidelines, under 'Reporting Guidelines'. Please confirm you have submitted this list.	Not Applicable	N/A

Data Availability

Data availability	Information included in the manuscript?	In which section is the information available? (Reagents and Tools Table, Materials and Methods, Figures, Data Availability Section)
Have **primary datasets** been deposited according to the journal's guidelines (see 'Data Deposition' section) and the respective accession numbers provided in the Data Availability Section?	Yes	Data Availability Section
Were **human clinical and genomic datasets** deposited in a public access-controlled repository in accordance to ethical obligations to the patients and to the applicable consent agreement?	Not Applicable	N/A
Are **computational models** that are central and integral to a study available without restrictions in a machine-readable form? Were the relevant accession numbers or links provided?	Not Applicable	N/A
If publicly available data were reused, provide the respective **data citations in the reference list**.	Not Applicable	N/A
